# Controlled modification of biomolecules by ultrashort laser pulses in polar liquids

**DOI:** 10.1038/s41598-017-05761-8

**Published:** 2017-07-17

**Authors:** Vitaly Gruzdev, Dmitry Korkin, Brian P. Mooney, Jesper F. Havelund, Ian Max Møller, Jay J. Thelen

**Affiliations:** 1 Department of Mechanical and Aerospace Engineering, University of Missouri, Columbia, MO 65211 USA; 2 Department of Computer Science, Bioinformatics and Computational Biology Program, Worcester Polytechnic Institute, Worcester, MA 01609 USA; 3 Charles W Gehrke Proteomics Center, University of Missouri, Columbia, MO 65211 USA; 4 Department of Biochemistry, University of Missouri, Columbia, MO 65211 USA; 5 Department of Molecular Biology and Genetics, Aarhus University, Forsøgsvej 1, DK-4200 Slagelse, Denmark; 6 Department of Biochemistry and Molecular Biology, University of Southern Denmark, Campusvej 55, DK-5200 Odense M, Denmark; 7 Christopher S. Bond Life Sciences Center, University of Missouri, Columbia, MO 65211 USA

## Abstract

Targeted chemical modification of peptides and proteins by laser pulses in a biologically relevant environment, *i*.*e*. aqueous solvent at room temperature, allows for accurate control of biological processes. However, the traditional laser methods of control of chemical reactions are applicable only to a small class of photosensitive biomolecules because of strong and ultrafast perturbations from biomolecule-solvent interactions. Here, we report excitation of harmonics of vibration modes of solvent molecules by femtosecond laser pulses to produce controlled chemical modifications of non-photosensitive peptides and proteins in polar liquids under room conditions. The principal modifications included lysine formylation and methionine sulfoxidation both of which occur with nearly 100% yield under atmospheric conditions. That modification occurred only if the laser irradiance exceeded certain threshold level. The threshold, type, and extent of the modifications were completely controlled by solvent composition, laser wavelength, and peak irradiance of ultrashort laser pulses. This approach is expected to assist in establishing rigorous control over a broad class of biological processes in cells and tissues at the molecular level.

## Introduction

Femtosecond laser pulses are capable of precisely controlling the pathway and final products of numerous chemical modifications of organic and inorganic molecules^1–4^. Both the frequency-resolved scheme^1^ that utilizes quantum interference between different reaction pathways and the time-resolved method^2^ that employs time-dependent motion of electron wave packets require minimizing all perturbations to the molecules under modification to keep the involved electron states coherently coupled. The ultrafast laser method of control was successfully transferred from a traditional case of compact molecules modified in the gas phase^1–6^ to biomolecules held in biologically natural environments, *e*.*g*., liquids^7–9^. Demonstrations of the laser control include modification of the rate of cis-trans transitions of rhodopsin^7^, variations of the rate of excitation energy transfer in photochemical reactions^8^, and modulation of energy-harvesting efficiency of photosynthesis^9^. Those results inspired consideration of the feasibility of laser control over biological processes at the molecular level^10, 11^. However, the success of the reported experiments is mainly be attributed to the ideal photochemical properties of the employed photosensitive biomolecules^7–11^. Establishing control over a broader class of proteins or peptides is highly challenging because strong interactions of the non-photosensitive biomolecules with dense room-temperature environment (*e*.*g*., liquids) destroy coherence of laser-induced electron excitations before they drive a biomolecule to the required final states or products^12^. Suppression or control of that de-coherence effect by laser pulses has been reliably performed only in the gas phase at low density of the perturbing environment^13^.

**Figure 1:**
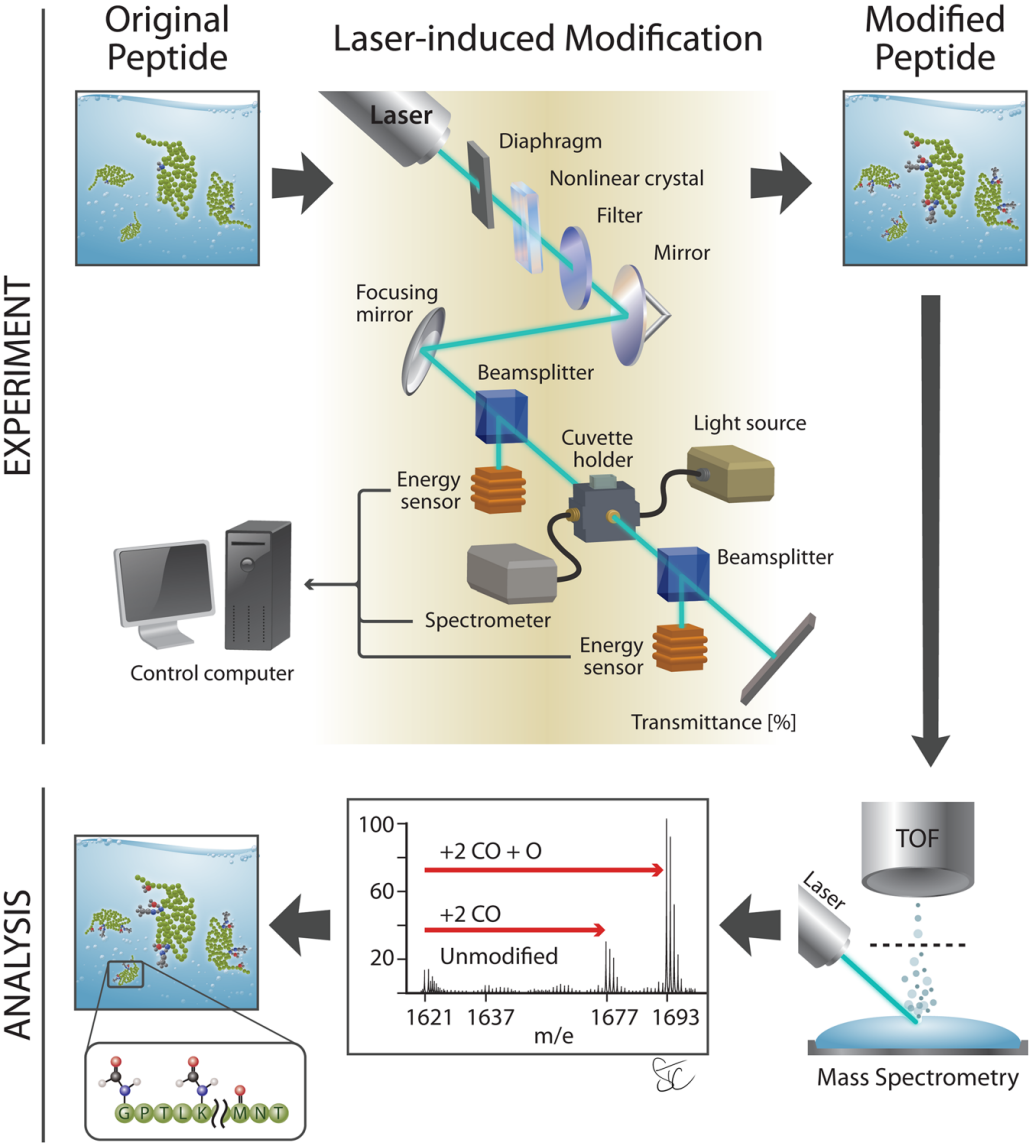
Experimental workflow for laser-induced modification of peptides and proteins in a liquid. Pulses generated by femtosecond laser were frequency-doubled in nonlinear crystal and forwarded to a quartz cuvette to modify liquid samples. The modifications were then detected by on-site optical transmittance spectroscopy. Peptide or protein molecules of the modified samples were analyzed by high-resolution mass spectrometry for detailed characterization of the modifications. Illustrated by Stacy Turpin Cheavens, MS, CMI © Copyright 2016 by The Curators of the University of Missouri, a public corporation.

Interactions between a biomolecule and the surrounding environment are inevitable for all physiological and biological processes, the majority of which take place in polar liquids. We propose a method that builds a foundation for non-coherent control of biological functions by permanent laser-induced chemical modifications that alter the properties of a broad class of biomolecules. This laser-biochemistry approach to controlling bioprocesses is based on the fact that a protein function is essentially dictated by the side chains of its amino acid residues. As proof of concept, we performed controlled chemical modifications of side chains of several peptides and proteins by laser pulses in room-temperature liquids containing oxygen from atmospheric-pressure air (Fig. 1). We hypothesize that the observed modifications are achieved by ultrafast laser excitation of harmonics of vibrational modes of polar molecules of a solvent or by electronic excitation via two-photon absorption. Both excitation processes are followed by intra- and inter-molecular energy transfer. This leads to ionization, possible dissociation of solvent molecules, and laser-assisted modification of specific polarized residue side-chains by the modified solvent. Extended experiments performed to identify the mechanisms of the biomolecule modification demonstrate that the modification output is completely controlled by laser parameters (wavelength and peak intensity of ultrashort pulses), peptide sequence, and composition of the liquids and ambient atmosphere.

## Major results

### Experiments on a test peptide

The first proof-of-concept experiments were performed with a synthetic peptide homologous to spinach nitrate reductase (GPTLKRTASTPFMNT-amide) containing a total of eleven different amino acid residues including one aromatic (Phe), two positively charged (Lys and Arg), and six polar (Ser, Thr, and Asn) residues. The peptide was dissolved in several polar liquids: de-ionized water; pure methanol; acetonitrile (ACN); methanol:water (98:2% v/v) mixture; and ACN:water (98:2% v/v) mixture. Pre-modification studies of optical-transmittance spectra of the solvents and low concentration (50 μM) solutions of the peptide in the liquids revealed shallow absorption bands located between 365 and 390 nm (see Supplementary Fig. S8). Those bands are produced by high-order vibration harmonics of the solvent molecules (e. g., refs 14–16 for water and refs 17–19 for methanol; also see Section 6 of Supplementary Information). For example, for water, they are the 8^th^ harmonic of O-H stretching mode combined with the scissor mode^16^. For methanol, the shallow absorption band can be produced by the 8^th^ harmonic of O-H stretching mode combined with either the second harmonic of CH_3_ rocking mode or second harmonic of C-O stretching mode.

Small samples (1 mL) of the peptide solutions and peptide-free solvents were irradiated with ultrashort (150 fs) laser pulses. The central wavelength of laser pulses was 386 nm to fit the long-wavelength side of the shallow absorption bands (Supplementary Fig. S8). The optical transmittance spectra were acquired by means of differential spectroscopy during and immediately after irradiation.

Nonlinear effects observed experimentally (see Supplementary Information, Sections 3 and 5), signaled that the solvents intensively interacted with laser pulses. Water was the only solvent for which its optical-transmittance spectrum was not altered by laser pulses, allowing direct detection of laser-stimulated modification of the peptide by optical spectroscopy (Supplementary Fig. S9). In contrast, after treatment with 120,000–360,000 laser pulses, the optical spectra of acetonitrile:water (98:2% v/v), methanol, methanol:water (98:2% v/v), and pure acetonitrile revealed strong changes in the far ultraviolet range of the optical spectrum (Supplementary Figs S9 and S10) that significantly exceeded the signal from the modified peptide (Supplementary Figs S9 through S15) and pointed to substantial modification of the solvents by laser pulses. Another signature of laser-solvent interactions was a replica of laser spectrum in differential transmittance spectra observed during laser treatment in each tested liquid except water (Supplementary Fig. S5). This may result from the scattering of laser light by micro-bubbles within the laser beam formed due to laser-stimulated decomposition of the solvents. The observed modification of methanol, accompanied by micro-bubble generation, and the stability of water are both consistent with previously published results on femtosecond-pulse laser modification of those liquids^20–22^. In particular, the laser intensity required to produce micro-bubbles in water was about 10^13^ W/cm^2^ ^20, 21^, which was not attainable in our experiments. We note that data on laser-induced ultrafast decomposition, micro-bubbles, and other nonlinear effects of ultrafast laser-acetonitrile interactions were not available in previous publications. In particular, modification of acetonitrile by laser pulses resulted in specific changes in the aroma of solvents with ACN content after laser treatment that was not mentioned in any previous publication.

**Figure 2:**
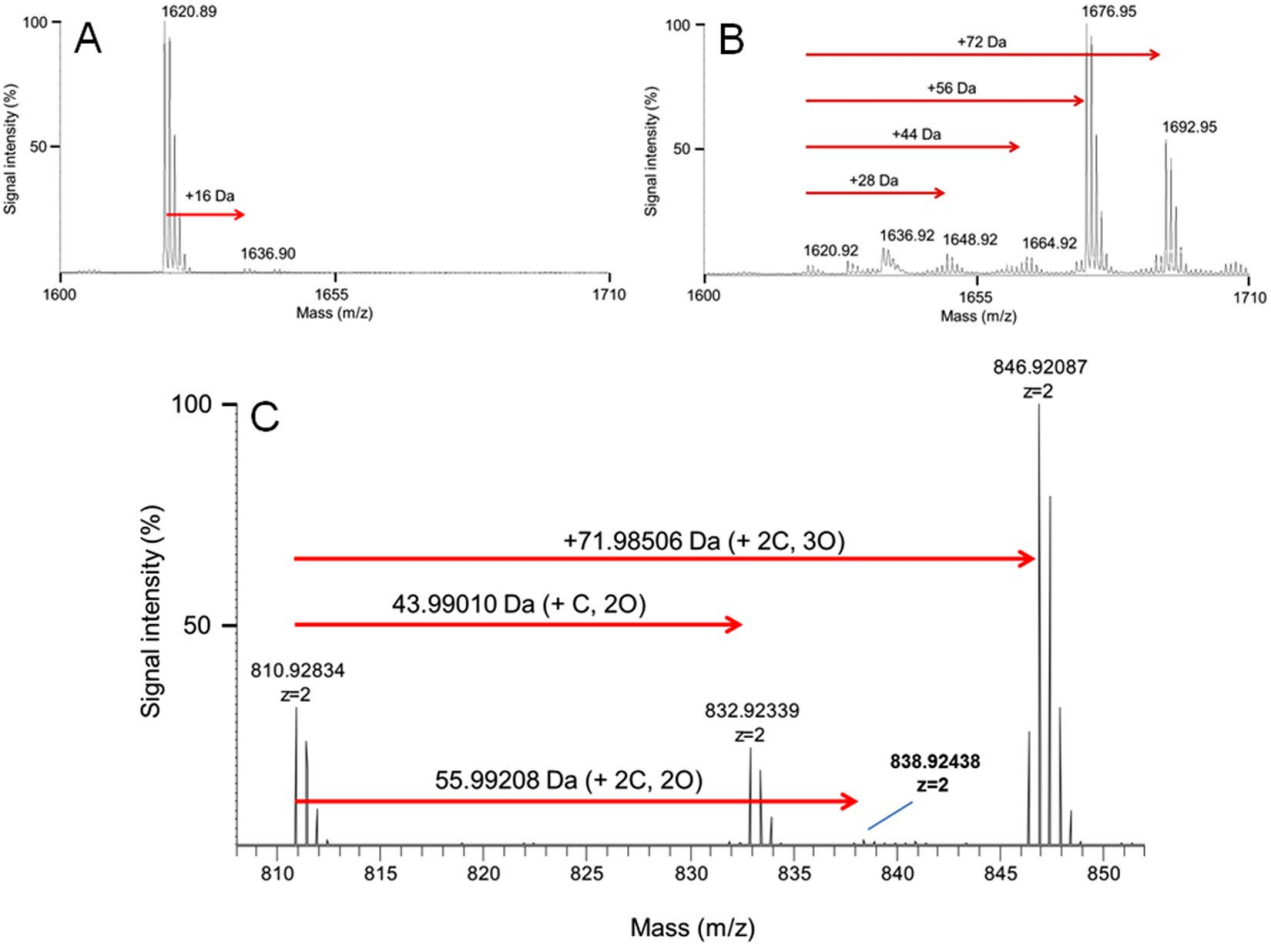
Mass spectra of laser-induced modifications of the GPTLKRTASTPFMNT-amide peptide in acetonitrile. (**A**) Control: MALDI-TOF mass spectrum (MS) of the peptide not subjected to laser irradiation. (**B**) Irradiated: MALDI-TOF MS of the peptide irradiated with 360,000 laser pulses at wavelength 386 nm with peak intensity 2.49 × 10^11^ W cm^−2^ (corresponding pulse energy is 293.9 ± 6.6 μJ; laser fluence 37.42 mJ cm^−2^). (**C**) High resolution ESI-Orbitrap MS spectrum of the irradiated test peptide. The signal at 810.92834 m/z is the unmodified peptide, which was spiked in to give a reference peak. The elemental composition of the Δmass between peaks was calculated using Elemental Composition Calculator v1.0 (http://mods.rna.albany.edu/masspec/Elcomp, Jeff Rozenski) with the following masses: (**C**) 12.000, H: 1.00782, N: 14.00307, O: 15.99492, S: 31.97200. The elemental compositions shown were the only possible combination below a mass deviation of 50 ppm from the theoretical value. In all cases, the peptide was resuspended in ACN:water (98:2; % v/v).

Following the commonly accepted concept^20–22^ we attribute the generation of micro-bubbles to the process of solvent ionization followed by possible chemical modification by the laser pulses. It had a threshold-type nonlinear dependence on laser intensity/fluence: for each solvent, the modification took place only at laser intensities exceeding a certain level (referred to as threshold of solvent modification) characteristic for each liquid. For example, the threshold intensity was about 13.7 MW/cm^2^ (corresponding fluence was 2.05 μJ/cm^2^ with a laser-spot diameter of 6.5 mm) in ACN:water (98:2% v/v) mixture. Since that threshold was almost two orders of magnitude lower than the threshold of peptide modification (see below), the nonlinear laser-solvent interactions dominated the optical response of the peptide solutions treated by laser pulses in this study. Therefore, the optical transmittance spectra did not give adequate information about the nature of the modifications on the peptide in those liquids, We used matrix-assisted laser desorption/ionization, time-of-flight mass spectrometry (MALDI-TOF MS) to identify the laser-induced effects on the peptide. For each MALDI-TOF MS analysis, non-irradiated peptide was used to confirm that the MALDI laser itself did not cause peptide modification (Figs 2A and S7 and S17 through S22).

MALDI-TOF MS revealed chemical modification of nearly all of the dissolved peptide in most tested solvents including ACN:water, ACN, methanol, methanol:water, and water (Fig. 2; also Supplementary Figs S7 and S17 through S22). In ACN:water (98:2% v/v), laser irradiation caused mass shifts of +28, +44, +56, and +72 Da from the native mass (Fig. 2B). Using high-resolution, high-mass-accuracy Orbitrap mass spectrometry, these mass shifts were attributed to single and double formylation (+CO = 27.98 ± 0.04 Da and +2 × CO = 56.01 ± 0.03 Da, respectively), single oxidation (+O = 16.08 ± 0.04 Da), and combinations thereof, for example, double formylation plus single oxidation (+71.98 ± 0.03 Da) (Fig. 2C). Although the relative amounts of those forms varied slightly among replicates, the conversion from unmodified to formylated forms (2CO plus 2CO+ MetOx) was essentially complete (97.6%; n = 6). The sites of modification were confirmed by *de novo* peptide sequencing using both MALDI TOF-TOF CID and Orbitrap HCD MS/MS (Supplementary Figs S23 and S24). Formylation always involved one or both of the primary amine groups (N-terminal Gly and internal Lys) while the oxidation took place exclusively at the side chain of Met (Supplementary Figs S23 and S24 and Supplementary Tables S6 and S7). Formylation only occurred in solvents containing acetonitrile. In contrast, Met oxidation was the major observed modification of the peptide in water, pure methanol, and in methanol:water (98:2% v/v) (Supplementary Figs S17 through S19). Laser pulses did not stimulate other detectable modifications, or peptide fragmentation, in any of the experiments.

### Dependence of the modification on laser parameters

**Figure 3:**
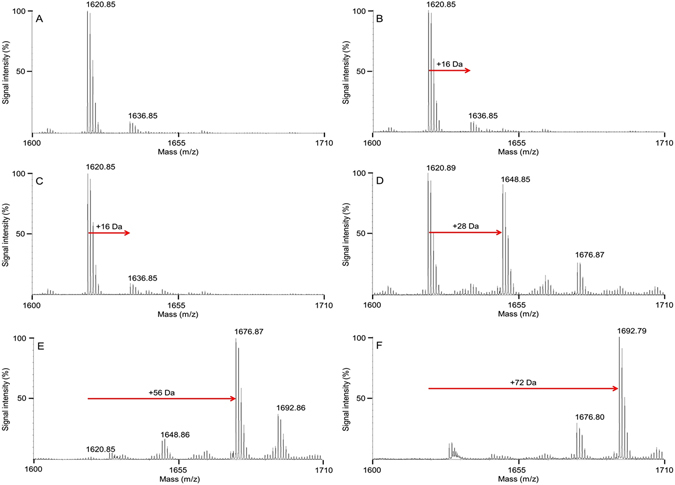
Mass spectra of the test peptide irradiated with 360,000 laser pulses at wavelength 386 nm in ACN:water (98:2% v/v). (**A**) Control (non-irradiated) peptide, (**B**) laser intensity 1.91 × 10^8^ W cm^−2^ (average pulse energy 9.5 μJ; fluence 28.7 μJ cm^−2^), (**C**) intensity 3.66 × 10^8^ W cm^−2^ (average pulse energy 18.2 μJ; fluence 54.9 μJ cm^−2^), (**D**) intensity 1.14 × 10^9^ W cm^−2^ (average pulse energy 56.8 μJ; fluence 171.3 μJ cm^−2^); (**E**) intensity 2.58 × 10^9^ W cm^−2^ (average pulse energy 128.5 μJ; fluence 387.1 μJ cm^−2^), and (**F**) intensity 2.49 × 10^11^ W cm^−2^ (average pulse energy 293.9 μJ; fluence 37.4 mJ cm^−2^). Modifications, including change in mass, are indicated with red arrows.

Reduction of laser wavelength to 257 nm did not produce significant impact on the modification results (Supplementary Figs S17 and S21). The mass spectra of the peptide modified by 772 nm laser pulses contained multiple higher m/z peaks, above the typical +28 Da and +56 Da (CO and 2xCO) modified peaks (see Supplementary Fig. S35). This suggests modification by 772 nm laser pulses is un-controlled, leading to multiple higher-order modifications. Variations of laser-pulse duration from 100 fs to 200 fs also did not significantly change the peptide modification observed at pulse width 150 fs. However, laser intensity significantly affected the type and relative amounts of the peptide modifications observed in pure ACN and in the ACN:water mixture at a fixed laser wavelength 386 nm (Fig. 3; also Supplementary Figs S20 and S21). Those modifications exhibited a threshold-type dependence on intensity with negligible amounts of a +28 Da modification at intensity below 1 GW/cm^2^ (Fig. 3B and C). The first modification observed at intensity above the threshold was +28 Da; a 1:1 signal intensity (unmodified:modified) suggested a 50% conversion rate (Fig. 3D). With increased laser intensity, almost 100% of the peptide was modified to a + 56 Da form (Fig. 3E). A minor amount of a +72 Da form was also observed and the modification at +28 Da was diminished (Fig. 3E). Finally, at laser intensities above 10^11^ W/cm^2^, the major peak was a +72 Da modified peptide and the amount of the +56 Da form was strongly reduced (Fig. 3F). Since the threshold of the peptide modifications was almost two orders of magnitude higher than the threshold of micro-bubble generation and solvent modification by the laser pulses, it was reasonable to expect that the modified solvent contributed to the peptide modification.

### Mechanism of the peptide formylation

The specific formylation (net addition of CO) of primary amines in ACN-water mixtures by laser irradiation was a surprise given that neither ACN (CH_3_CN) nor water contain a formyl group. Since no peptide formylation was observed in methanol and water, participation of at least one carbon of acetonitrile in formylation was a reasonable assumption. Generation of micro-bubbles by laser pulses in ACN:water (98:2% v/v) suggested a possible mechanism for peptide modification: laser-induced decomposition of the solvent followed by laser-stimulated peptide interaction with products of the decomposition. The origin of carbon and oxygen in the formyl group was further tested using 2-^13^C-labelled ACN (^13^CH_3_CN) and ^18^O-water (H_2_^18^O) as diluents (Supplementary Figs S26 through S29). Laser irradiation of the peptide in ^13^CH_3_CN:H_2_^18^O (98:2; % v/v) showed incorporation of ^13^C-labelled formyl groups, while no ^18^O incorporation was detected (Supplementary Figs S27 and S28). Additionally, peptide irradiated in ACN:H_2_^18^O (98:2; % v/v), MeOH:H_2_^18^O, or pure H_2_^18^O showed no ^18^O incorporation into formyl groups (Supplementary Figs S26 and S29). In pure ACN, double formylation plus Met-oxidation (+72 Da) was the dominant laser-induced modification (Supplementary Fig. S21). This fact and data obtained using isotope-labeled solvents led us to consider dissolved oxygen (O_2_) in the laser-treated liquids as a source of oxygen for the formyl groups because all experiments were conducted under atmospheric conditions. To test this hypothesis, peptide solutions in ACN:water mixture (90:10% v/v) were sparged with argon gas prior to laser treatment. Under control conditions (*i*.*e*., without sparging), formylation and Met-oxidation occurred (Supplementary Fig. S30A). However, following argon sparging, formylation was eliminated (Supplementary Fig. S30B). Combining all these data, we concluded that the beta-carbon of acetonitrile decomposed by laser pulses and solvent-dissolved oxygen were the sources of carbon and oxygen, respectively, for the laser-induced peptide formylation.

The test peptide contained both primary (N-terminus, Lys) and secondary (Arg) amines. However, no formylation of Arg was observed indicating that a positively charged side chain is not sufficient to ensure laser-stimulated formylation. The nature of Lys modification was tested by laser treatment of hydrated Lys under the same conditions as the test peptide. No formylation of free Lys was detected by mass spectrometry (Supplementary Fig. S31) indicating that only polymeric lysine (*i*.*e*., within a peptide or protein) was capable of accepting the formyl groups.

### Experiments on other peptides

**Figure 4:**
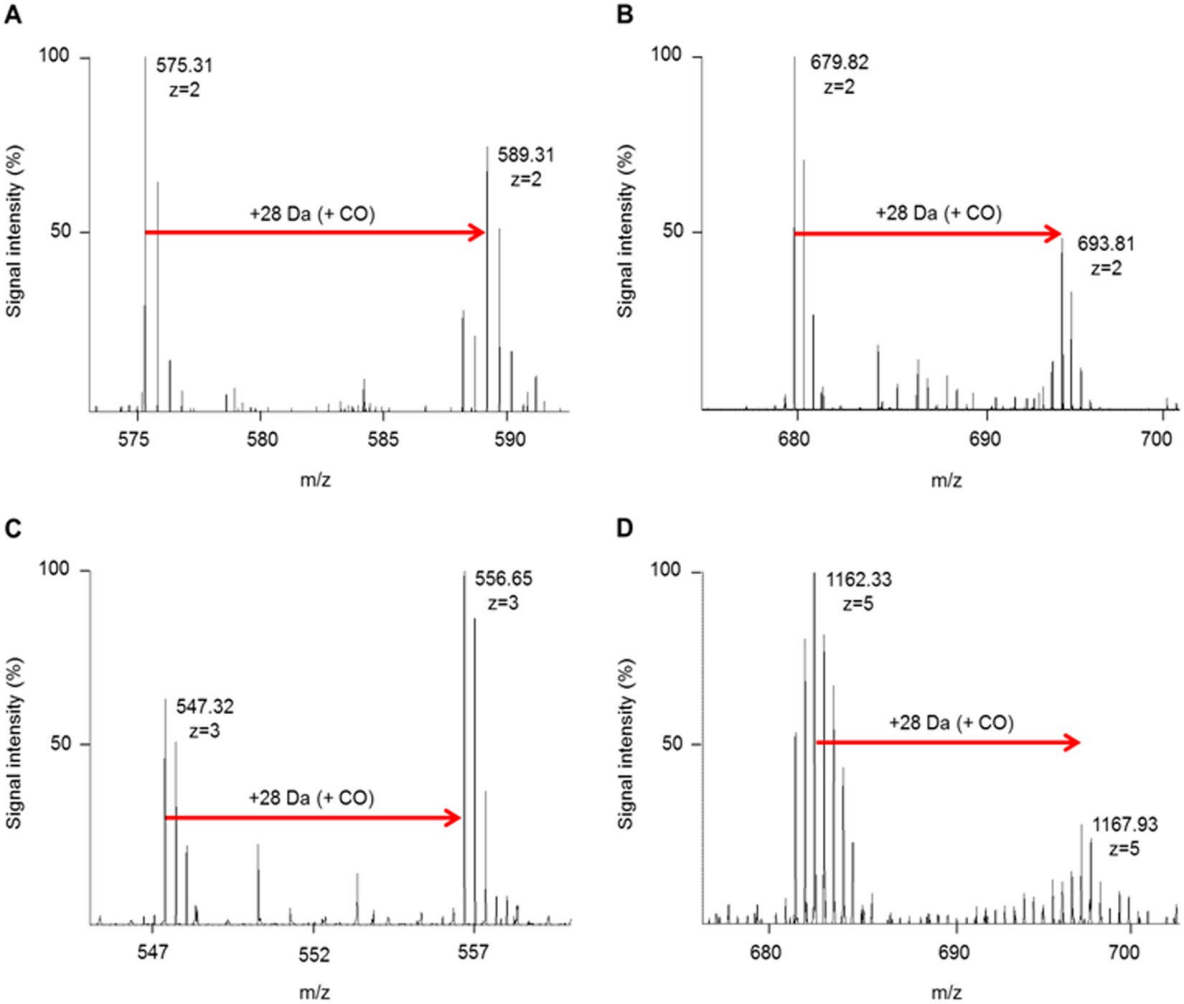
Laser-induced modification of human serum albumin (HSA) peptide standard and insulin. Orbitrap ESI-FTMS mass spectra of laser-induced formylation of HSA standard peptides (**A**) LVNEVTEFAK, (**B**) AVMoxDDFAAFVEK, and (**C**) KVPQVSTPTLVEVSR. Laser-induced formylation of human insulin (**D**) was also observed. The unmodified forms of the peptides and insulin correspond to the first labeled peak in each mass spectrum. The charge state of each peptide or protein observed by electrospray ionization is indicated below the m/z value. The modified forms have a net addition of CO (+28 Da). In all cases, modification was conducted in ACN:water (98:2; % v/v), at room temperature in air by 300,000 laser pulses at wavelength 386 nm with average pulse energy 269.5 μJ at intensity 5.19 × 10^10^ W cm^−2^.

After demonstrating the feasibility of controlled laser-induced peptide modification in liquids in ambient air, we tested a seven-peptide standard, derived from human serum albumin (HSA) diluted in ACN:water (98:2% v/v) under the same laser irradiation as the test peptide (Fig. 4; also Supplementary Fig. S32). To confirm that formylation was not an artifact of the MALDI technique, data for the seven peptide HSA mix were acquired by static nano-electrospray (nESI) on an LTQ Orbitrap XL mass spectrometer. In contrast to MALDI-TOF MS data, in nESI most peptides are ionized with a charge-state greater than one. Five peptides were present as 2+ charge-state ions and one showed prominent 3+ ions. All seven peptides exhibited laser-induced formylation. The results show that single formylation on primary amines was the predominant chemical modification produced by irradiation with femtosecond laser pulses at 386 nm (Fig. 4; also Supplementary Fig. S32). There was evidence for peptides with double formylation, but those species were present at much lower levels relative to the singly formylated species. Only one of the seven peptides contained a Met residue (AVMDDFAAFVEK). After treatment, this peptide was present exclusively as the Met-Ox or the Met-Ox+ formylation forms, in approximately a 2:1 ratio (Supplementary Fig. S32). The only peptide without a Lys residue (YLYEIAR) also showed formylation. However, the data for this peptide are equivocal due to low signal intensity for both the unmodified and modified forms (Supplementary Fig. S32).

### Experiments on insulin

We finally extended the femtosecond laser modification method to treatment of an intact protein – mature human insulin. Orbitrap nESI data demonstrated that a single formylation event was induced on insulin (Fig. 4D; see also Supplementary Fig. S33) confirming that proteins are also amenable to laser-induced formylation. In contrast to the data for a single peptide or seven-peptide mix, the stoichiometry of formylation of insulin was only about 25%, suggesting that additional tuning of the laser parameters (wavelength, duration, or intensity) might be required for intact proteins.

## Discussion

The data presented herein demonstrate that femtosecond laser pulses can produce formylation specifically on primary amines of peptides and proteins. The most remarkable feature of this process is the nonlinear threshold-type dependence of peptide-modification outcome on laser intensity (Fig. 3) with the capability to control the type of modification by tuning the laser intensity above the threshold. However, the ultrashort duration of laser pulses and proper wavelength play a crucial role in attaining that level of control on the chemical modification of the peptides. From the point of view of the fundamental mechanism, we consider a modification of a quantum system by laser radiation. A Schrödinger equation for this kind of interaction must include time-dependent electric field of laser radiation or corresponding vector potential as a perturbing term. In turn, the electric field of radiation is directly related to intensity (since intensity is directly proportional to squared electric field) rather than to total energy or fluence of radiation pulse. Since intensity of laser pulses was quite high in the experiments, the radiation can be treated classically, *i*. *e*., like a wave with certain amplitude and phase. In this connection, it is more correct to discuss the influence of laser intensity on peptide modification rather than influence of the total number of absorbed photons or total pulse energy. Therefore, the microscopic mechanisms of the reported modification should be discussed in terms of electron excitation to certain energy levels followed by relaxation and corresponding energy transfer.

We first consider the cases of water and methanol since those liquids have received the most detailed characterization in previous studies^23–30^. Close proximity of the laser spectrum (at wavelength 386 nm) to the 8^th^ harmonic of OH stretching mode coupled to the scissor mode in water and to the CO stretching or CH_3_ rocking mode in methanol suggests that laser photons could be absorbed by those vibration modes of the solvent molecules. In both cases, the strong vibration excitation can destroy the excited molecules by bond breaking since energy of the 8^th^ OH stretching harmonic is close to the level of molecule dissociation. However, the OH vibration excitation can relax to lower energy levels by intra-molecular vibrational-energy redistribution (IVR) and transfer the absorbed energy to the vibration modes coupled to the OH stretch^17, 21–30^. As the next step, the vibration states can further relax to the ground state of the molecules. The energy released during that relaxation can be transferred to surrounding molecules via inter-molecular energy transfer associated with perturbation and/or re-arrangement of hydrogen bonds^17, 24–28^. Domination of either of those two paths (i. e., molecule dissociation or multi-step relaxation) is essentially controlled by a ratio of pulse duration to the characteristic time of vibration-mode relaxation. The relaxation time of the lowest excited state of the OH stretch is about 250 fs for water^23–25^ and about 750 fs for methanol^28, 29^. However, recent experimental data^26, 27, 30^ suggest that the relaxation of higher OH stretching harmonics can be significantly faster (50–100 fs for water and 450 fs for methanol). Since micro-bubbling was not produced in water, a reasonable assumption is that water was not modified (i. e., decomposed or ionized) at any remarkable degree^20, 21^, and absorption of laser photons was rapidly followed by relaxation of the OH stretching states and IVR that resulted in energy transfer to the vibration modes coupled to the OH stretch. The sub-100-fs relaxation time of the OH-stretch harmonics^26, 27^ is shorter than pulse duration and is also supportive for this assumption. Moreover, the rapid multi-step relaxation path is in agreement with the experimental data on peptide modification in isotope labelled water and does not contradict the conclusion about the contribution of dissolved oxygen in the modification.

In contrast to water, the relaxation time of the OH-stretch harmonics substantially exceeds pulse duration in methanol^28–30^. Therefore, the OH vibrations of methanol molecules can be strongly excited within the duration of a single laser pulse, and corresponding energy levels can be significantly populated by the laser pulse. Since the energy of the 8^th^ harmonic is close to the energy of bond breaking, this excitation creates a situation favorable for breaking the O-H bonding before any remarkable relaxation by energy transfer to other vibration modes. Potentially, this process can produce CH_3_O and H fragments in the liquid, however, the former fragment would produce formylation of the peptide in methanol which was not detected in our experiments. Other possible modification paths can include breaking of the CO bonding due to partial energy transfer from the OH stretch to harmonics of the CO stretch^28–30^. It is remarkable that excitation of the OH stretch followed by energy transfer to the CO stretch is favorable for dissociation of methanol molecules into CH_3_ and OH fragments^17, 22^. The active OH fragment of the dissociated methanol molecule could be attached to a positively charged peptide side-chain to produce the reported Met oxidation of the peptide in methanol.

The nonlinear dependence of the micro-bubble generation on laser intensity suggests participation of a nonlinear mechanism of either energy absorption or energy transfer. Several options can be considered for the mechanisms of that nonlinearity. First, some minimum population of the excited vibration energy levels can be required to trigger either ionization or dissociation of the solvent molecules by laser pulses. This process must be sensitive to the total energy of a laser pulse. We checked this option by generation of micro-bubbles by expanding a laser beam from 3 mm to 6.5 mm at constant pulse energy. While the micro-bubbles were visible at 3 mm beam diameter (see Supplementary Fig. S4), they became completely invisible in 6-mm-diameter laser beam. That suppression of the micro-bubbling suggests that the process was driven by intensity rather than by total pulse energy. Another option can be associated with intensity-dependent perturbations of the liquids and inter-molecular energy transfer that stimulate molecule up-transition from the energy of the 8^th^ OH-stretch harmonic to the energy of molecule dissociation. The third option is the two-photon absorption of laser photons so that a single portion of absorbed energy corresponds to wavelength 193 nm at input laser wavelength 386 nm. That wavelength is close to the absorption band produced by electronic excitation^31^. The electron excitation can potentially result in the ionization followed by breakdown of the liquid^20, 21^ and decomposition of the excited solvent molecules. However, electron excitation can also relax via the vibration states or electrons, and the relaxation can take place via the two processes discussed above: dissociation of the solvent molecule or transfer of the absorbed energy to highly-excited vibration modes^17, 25^. The fact of strong micro-bubbling of methanol, acetonitrile, and acetonitrile:water mixtures suggest that the relaxation path can be excluded from the consideration at intensity required for the peptide modification.

In case of acetonitrile and ACN: water mixtures, the situation is less certain. We note that ultrafast laser-induced modification of acetonitrile has not been studied in detail thusfar although significant attention has been paid to ultrafast studies of energy relaxation in water molecules and clusters dissolved in acetonitrile^32–35^. Those studies suggest that the fastest intra-molecular relaxation takes about 170–200 fs in acetonitrile^33, 35^, but excitation of OH stretch of water molecules diluted in ACN can relax within 100 fs^34^. Therefore, the pulse duration utilized in the experiments was favorable for strong excitation of the acetonitrile component of the solvent without affecting the water content of the solvent. This assumption completely agrees with the result of the experiments in isotope-marked water and ACN and suggests that selecting an appropriate duration of the ultrashort pulses and their wavelength can be critical for driving the reported above peptide modifications. However, the lack of published information is not favorable for detailed analysis of involved excitations and forces us to hypothesize about possible mechanisms of the peptide modification in the liquids containing ACN. In this connection, we note that previously reported reversible decomposition of ACN in the presence of metal catalysts^36^ suggested two pathways for the ACN decomposition. Pathway A was associated with hydrolysis based on presence of water:

1 $${{\rm{CH}}}_{3}{\rm{CN}}+2{{\rm{H}}}_{2}{\rm{O}}\leftrightarrow 2{\rm{CO}}+2{{\rm{H}}}_{2}+{{\rm{NH}}}_{3};$$

Pathway B was attributed to a net reaction given by the following equations^13^:

2 $${{\rm{CH}}}_{3}{\rm{CN}}+2{{\rm{H}}}_{2}{\rm{O}}\leftrightarrow 2{\rm{CO}}+2{{\rm{H}}}_{2}+{{\rm{NH}}}_{3};$$

3 $$2{{\rm{CO}}}_{2}+2{{\rm{H}}}_{2}\leftrightarrow 2{\rm{CO}}+2{{\rm{H}}}_{2}{\rm{O}};$$

and the net reaction

4 $${{\rm{CH}}}_{3}{\rm{CN}}+2{{\rm{CO}}}_{2}\leftrightarrow 4{\rm{CO}}+{{\rm{NH}}}_{3}$$

For both pathways, the formation of ammonia explains the specific change in the odor of ACN-based solvents observed after laser treatment. Participation of water molecules in production of formyl groups according to the pathway of equation (1) is not consistent with the absence of ^18^O in formyl groups (Supplementary Figs S26 and S29) and the results obtained in water-free ACN. The pathway described by equations (2–4) assumes participation of dissolved carbon dioxide (from air) that contributes both oxygen and carbon to the formyl groups. A participation of carbon dioxide would provide an explanation for the sparging experiment where formylation was abolished, but Met oxidation was not. However, products of the laser modification of acetonitrile and acetonitrile:water mixture were not available for analysis. FTIR spectroscopy demonstrated no difference between laser-modified and original acetonitrile (Supplementary Fig. S34). This fact could be interpreted in terms of decomposition of ACN into gaseous products that depart from the liquid prior to FTIR analysis. It can also be explained by low concentrations of the modification products, thus eluding detection. Equations (2) through (4) suggest that the compounds participating in the peptide modifications are ACN (it is consumed), CO_2_ (consumed), CO (it is produced, but it is incorporated into the modified peptide, when present), and NH_3_. Assuming that all molecules of the peptide were modified by attaching a single CO group, such a modification would change those compounds by 12.5, 25, 50, and 12.5 micromolar, respectively (since the peptide concentration was kept at 50 micromolar in all experiments). Those concentrations should be compared to the starting concentrations of 19 and 1.1 molar for ACN and water in ACN:water mixture (98:2% v/v). Obviously, laser treatment could produce significantly more solvent-modification products than the minimum amounts required just for the peptide modification. However, in the worst-case scenario, the minimum concentration of the solvent decomposition products can be three to four orders of magnitude lower than the starting concentration of the solvent components. Even if the products have strong absorption in the infrared region, they will not be detected by FTIR.

We also note that two-photon absorption (equivalent wavelength is 193 nm) by acetonitrile cannot be excluded from consideration since it can produce electronic excitation of the molecules^31^. Moreover, the strong nonlinear dependence of the two-photon absorption rate on laser intensity is favorable for justification of the threshold-type dependence of micro-bubble generation on laser intensity. In that case, the modification pathway can be similar to that discussed above for water and methanol: from two-photon electronic excitation either to the ionization and breakdown or to strong vibration excitation followed by decomposition of the excited acetonitrile molecules. Additional research, beyond the scope of this study, would be required to identify what absorption path dominates under the specific laser parameters in those solvents.

Although clarification of a specific mechanism of the reported peptide modification requires extra research efforts, the reported here chemical modification of peptides is relevant for control of biological processes since formylation can block Lys residues, thereby altering the functional properties of targeted proteins^37^. Traditional approaches to formylation typically employ biochemical methods. For example, in living cells, Lys formylation of specific residues in specific proteins requires dedicated enzymes using the coenzyme (vitamin) folate to carry the formyl group^38^. Formylation can also occur on histones as the result of a more random process starting with oxidative DNA damage and proceeding via the formation of formyl phosphate^39^. Additionally, *in vitro* methods of formylation have been developed, but they are slow and/or give low yields^40–44^. Femtosecond laser pulses elicit the highly effective and controlled formylation of specific Lys residues and N-termini in peptides and proteins, and produce formylated biomolecules at almost 100% yield at high rate unreachable for the traditional methods. The higher efficiency of laser-induced chemical modifications, compared to non-laser methods, was recently confirmed for DNA-protein^45^ and peptide-peptide^46^ cross-linking produced by ultrashort UV laser pulses at wavelength 263 nm. The laser cross-linking^45^ was performed in living cells at an intensity of 10^9^–10^10^ W/cm^2^.

Laser-induced formylation of biomolecules belongs to the class of laser-assisted reactions of chemical synthesis^47^ that are rarely reported^45–47^. Nearly 100% modification of primary amines only suggests that corresponding biomolecule-solvent hydrogen bonds are the most effectively converted into internal biomolecule covalent bonding by proper laser excitation of interacting molecules. This result demonstrates that the reported approach can be a universal method to produce a variety of laser-assisted biochemical synthesis reactions in polar liquids. Moreover, our data provide a deeper insight into fundamentals of ultrafast laser-biomolecule interactions in liquids and mechanisms of laser-stimulated biochemical synthesis reactions by transformation of weak inter-molecular bonds into stronger intra-molecular bonds. In summary, treatment of peptides and proteins with femtosecond laser pulses leads to a precise irreversible chemical modification of biomolecules in polar liquids enriched with dissolved air and potentially provides a means for well-defined optical control of biological processes at the molecular level.

## Materials and Methods

### Materials

Experiments were done with the test peptide (sequence: GPTLKRTASTPFMNT-amide, RP-HPLC analysis – peptide is >92% pure; molecular weight 1621 g/mol; origin: Multiple Peptide Systems, San Diego, CA, USA, lot number K39-96//02274-11(16–27)); human insulin (recombinant, expressed in yeast (proprietary host); molecular weight 5807.57 g/mol; purity ≥98% (HPLC); origin: Sigma-Aldrich, Denmark, lot number SLBC1253V; part number: I2643-25MG); HSA peptide standard (peptides of the standard are listed in Supplementary Table S1; origin: Agilent Technologies/Supelco Analytical, USA, lot numbers LB85434 and LB91722, Agilent part number G2455-85001); and lysine monohydrochloride (formula: HO_2_CCH(NH_2_)(CH_2_)_4_NH_2_ · HCl; tissue culture grade; molar mass 146.19 g/mol without HCl and 182.65 with HCl; origin: Fisher Scientific, USA, lot number 037340, Fisher Scientific part number: BP386-100/FL-03-0398). To prepare the solvents, we used de-ionized ultrapure water (obtained right before preparation of each sample by multiple filtering, distillations, and de-ionizing under standard conditions (milliQ, 18 MOhm, <5 ppm total organic carbon)); acetonitrile (abbreviated as ACN below; structural formula CH_3_CN; grade – LC-MS CHROMASOLV >99.9%; manufacturer: Fluka Analytical, country of origin: USA, lot number 75696LMV, part number 34967-4 L); methanol (formula: CH_4_O; grade – CHROMASOLV Plus, for HPLC, >99.8%; manufacturer: Sigma-Aldrich; country of origin: Trinidad, Tobago; lot number SHBB2585V; Sigma-Aldrich part number: 179337-4 L; CAS: 67-56-1); isotope-labeled water-^18^O (molecular weight 20.02 g/mol; 97% atom. H_2_^18^O; manufacturer: Aldrich Chemistry, country of origin: USA, lot number EB1556V, Aldrich part number 329878-250MG, CAS number 14314-42-2); isotope-labeled acetonitrile-2-^13^C (structural formula: ^13^CH_3_CN; manufacturer: Aldrich Chemistry, country of origin: USA, lot number EW1267V, Aldrich part number 277223-250MG, CAS number 1722-09-4). All dry samples were stored in used to prepare the solvents were stored in original dark-glass bottles capped with original screw caps.

### Experimental Design

Laser treatment was done by trains of pulses from Clark-MXR CPA-2010 Ti:sapphire femtosecond laser system with regenerative power amplifier under the following parameters:

- Fundamental wavelength (also see Supplementary Table S2): 772.00 nm;
- Pulse repetition rate: 1,000 pulses per second;
- Polarization: linear, horizontal;
- Pulse full width at the 50% level of peak intensity: 150 fs (by autocorrelator).

Laser wavelength was reduced down to 386 and 257 nm by frequency doubling in a BBO nonlinear crystal (type I SHG; dimensions 7 × 7 × 0.75 mm; P-coating; θ = 30.5 deg.; origin: United Crystals Company, China) and frequency mixing in another BBO crystal (type I THG, dimensions 7 × 7 × 0.5 mm; P-coated; θ = 47.8 deg.; origin: United Crystals Company, China) correspondingly as depicted in Supplementary Fig. S1. The three wavelengths were utilized to check influence of laser parameters on the modification of peptides. Laser parameters are summarized in Supplementary Table S2.

The laser beam was slightly focused by a focusing mirror (focus distance f = 500 mm) with a focal point 5 mm behind the rear surface of the cuvette. Laser-spot diameter varied from 6.5 mm down to 1 mm on the front wall of a quartz cuvette to check intensity influence on peptide modification. All values of pulse energy, fluence, and intensity shown above were evaluated at the front face of a cuvette from directly measured values. However, they can be converted to effective values of intensity and fluence in liquids utilizing transmittance of two interfaces of the front cuvette wall directly measured with sensors S1, S2, and S3 (Supplementary Fig. S3B). At 386 nm, transmittance was 93% for an empty cuvette and 95% for a cuvette with ACN:water mixture (98:2% v/v) for the entire range of laser-pulse energy. For experiments on bio-molecule treatment, a beam splitter installed in front of the cuvette (Supplementary Fig. S1) was a thin (0.15 mm thickness) glass slide that reflected about 11% of incident laser energy to the energy sensor. The beam splitter was calibrated and checked before each experiment. Pulse duration was controlled using beam splitter 1 (Supplementary Fig. S1) that forwarded small amount of laser pulses to an autocorrelator PulseScout (manufacturer: Newport Corp., USA).

Laser-induced modification of each sample was detected by measuring spectrum of optical transmittance (Supplementary Fig. S3A) with a 3-channel UV-IR high-resolution spectrometer (model AvaSpec-3648-USB2-RM, see Supplementary Table S3) and two continuous-wave light sources (models AvaLight-D-S-DUV and AvaLight-Hal-S). The spectral range from 185 nm to 500 nm contained the most prominent information about the modifications at a resolution of 0.05 nm. The beams from laser and the spectroscopy sources were propagated in mutually perpendicular directions in the cuvette (Supplementary Fig. S3A) to minimize the contribution of scattered laser light to transmittance spectra. At wavelength 386 nm, scattered laser light was not observed in any transmittance spectrum at laser intensity below 13.7 MW/cm^2^ (equivalent fluence was 2.05 μJ/cm^2^ at 6.5 mm diameter of input laser spot).

For each experiment, 5 to 6 ml of liquid sample was prepared. Each fresh solution was split into equal portions of 1 ml each and kept under the same environment during the experiments. One of the portions was kept untreated as control for mass spectroscopy. Each portion for laser treatment was placed into clean 4-window fluorescent cuvettes (Starnacell, USA; part number 3-Q-10; 10 mm optical path) made of UV-grade fused silica. The volume of each portion was minimized to provide reliable overlapping of the beam of spectroscopy light source with laser beam without coming into contact with the cuvette walls or the meniscus surface on top of the solution.

Illumination of each sample was done by trains of pulses. Each train contained 60,000 laser pulses and lasted for 60 seconds. After each train of laser pulses, the solution was mixed with a glass needle. For example, exposure to a total of 480,000 laser pulses lasted for 8 min and included 8 mixings by the glass tool. Solution temperature was measured during the experiments by a thermo-couple attached to the bottom of the cuvette and was found to vary by no more than 3 K at the longest exposure. Transmittance spectra were acquired during and after each train of laser pulses by the spectrometer using *the procedure of differential spectroscopy*: an untreated portion of the same solutions was utilized as a reference; prior to laser treatment, transmittance of a sample was normalized to the reference to make a reference line at the 100% level; spectra taken following the normalization procedure depicted deviations of transmittance of the modified solution from the reference level. For all samples, the spectrum deviations detected during the laser treatment strongly increased during the first 45,000–50,000 pulses of a pulse train, then saturated and varied by no more than 1% of the normalized transmittance (the measurement error for the transmittance spectroscopy) for the rest of the pulse train. This fact was interpreted as total modification of the part of the sample within a laser-irradiated volume. The liquid was mixed by a single-use glass tool after each pulse train and prior to acquisition of a post-train transmittance spectrum. Each sample was exposed to as many trains of laser pulses as required to observe the maximum train-to-train modification of the difference transmittance spectrum at the 1% level. Immediately after the laser treatment and acquisition of the final transmittance spectrum, each sample was transferred by single-use glass pipette to a capped glass container and stored at −20 °C.

### Preparation and storage of liquid samples for laser treatment

All liquid samples were prepared at room temperature (21 °C) in air. The concentration of the test peptide and HSA peptides was 50 ± 2 µM for all experiments. The concentration of insulin was about 10 µM due to poor solubility in ACN:water (98:2% v/v). Fresh sample was prepared for each experiment each time. Each sample was kept at laboratory temperature in air starting from preparation until the end of laser treatment. Laser-treated samples were kept at −20 °C until processing for mass spectrometry. For each solvent that contained water, the following protocol of sample preparation was used: small amount of dry powder of a biological material was weighed with an accuracy of ±10 μg and placed into a glass tube; 100–150 μL of water were added to the glass tube with the powder to fully dissolve the powder; required amount of the other component of the solvent (i. e., water, methanol or ACN) was added to obtain proper concentration and volume. For most experiments in water:ACN mixtures, water content was 2% (v/v) versus 98% content of ACN. For tests on variable content of water, the amount of water was varied from 1% (v/v) up to 90%.

### Extra laser effects

Increase of peak intensity above a certain level resulted in generation of micro-bubbles and spectrum broadening. Micro-bubbles indicated laser-induced modification of the solvent and were observable within the laser beam by eye at intensity 2.0 GW/cm^2^ and higher (Supplementary Fig. S4). They produced prominent scattering of laser light in the direction perpendicular to propagation path of laser beam that was acquired by the spectrometer and detected as a replica of the laser spectrum in transmittance spectra acquired during laser treatment (Supplementary Fig. S5A). With reduction of intensity, the bubbles became less visible, and the replica of the laser spectrum reduced. At intensity below 2.0 GW/cm^2^, micro-bubbles became invisible by eye, but they still produced scattering at the level of few percent on top of the transmittance spectrum. The peak of the scattering signal taken at laser wavelength (e. g., at 386 nm) after subtraction of the scattering-free transmittance at that wavelength exhibited a threshold-type dependence on laser intensity and linear dependence on laser intensity above the threshold. That linear scaling allowed estimation of threshold of micro-bubble generation. For example, the threshold was 13.7 MW/cm^2^ for ACN:water (98:2% v/v) at wavelength 386 nm. The threshold of the micro-bubble generation was almost two orders of magnitude lower than the threshold intensity required for initiating the peptide modification (see Fig. 3).

Further evidence for laser modification of solvents was the change in the smell of the liquids with ACN content, from a typical ACN smell to a sharp and striking smell. Solvents containing water, methanol and mixtures thereof did not change their smell after laser treatment.

Spectrum broadening due to self-phase modulation led to enrichment of laser pulse spectrum with extra frequencies^48^ and resulted in an increase of spectrum width of the laser line with increase of laser-pulse energy (Supplementary Figs S5B and S6) and in the formation of a significant long-wavelength shoulder of the spectrum (Supplementary Fig. S6B). The presence of those effects was checked by observation of a spot of white light on the screen installed behind the tested cuvette (see Supplementary Fig. S4B). Those and other ultrafast nonlinear effects were not studied in details since that was beyond the scope of this research effort.

### Matrix-assisted laser desorption/ionization time-of-flight mass spectrometry

Laser irradiated and control peptides were examined using matrix-assisted laser desorption/ionization time-of-flight (MALDI-TOF) mass spectrometry. Peptide solutions were stored at −20 °C following laser irradiation. All samples were diluted 1:5 (v/v) with matrix solution (5 mg/mL α-cyano hydroxycinnamic acid, CHCA, in 60% (v/v) acetonitrile, 0.3% (v/v) trifluoroacetic acid, 10 mM ammonium phosphate, in water). An aliquot (0.5 μL) was spotted onto an ABSciex 192 + 6 stainless steel MALDI target and allowed to co-crystalize at room temperature. Once dry, the target was loaded into an Applied Biosystems 4700 MALDI TOF-TOF mass spectrometer (now Sciex, Framingham, MA, USA). The instrument was operated in positive-ion reflector automatic mode with the following parameters: mass range 1000–2200 m/z, focus mass of 1650 m/z; matrix = CHCA; collect 50 shots/sub-spectrum, accept all sub-spectra, 5000 total shots; random, uniform acquisition pattern; fixed laser intensity of 3800; digitizer bin size 0.5 ns. Spectra were processed using the following parameters: internally re-calibrated using 1620.853 peptide ion, +/− 0.5 m/z, minimum signal/noise of 20. Default calibration (when 1620 ion was missing) was achieved using “plate model and default” external calibration using the 4700 calibration mix (Sciex); peak detection: minimum signal/noise of 20, local noise window 200 m/z, minimum peak width at FWHM of 2.9.

Peptide fragmentation MALDI TOF-TOF MS/MS spectra were acquired using the following parameters: manual peak picking from the MS spectrum using right-click and drag; data acquired in automatic positive-ion 1KV mode with CID gas off; precursor mass defined in Daltons, absolute mass window for peak isolation of −1.0 Da, +1.5 Da; metastable ion suppressor on, with optimized precursor, acquire 4 sub-spectra; CHCA set as matrix; 50 shots/sub-spectrum, accept all sub-spectra, 10000 total shots; random, uniform acquisition pattern; fixed laser intensity of 4500; digitizer bin size 1.0 ns. MS/MS spectra were processed as follows: default calibration was achieved using fragmentation of the angiotensin peptide; peak detection: minimum signal/noise of 5, local noise window of 200 m/z, minimum peak width at FWHM of 2.9. Data were then examined using Data Explorer software V4.6 Build 111.

### Additional mass spectrometry analysis

In addition to MALDI-TOF MS and TOF-TOF MS/MS, an LTQ Orbitrap XL mass spectrometer was used to analyze the peptide, human serum albumin seven peptide mix, and insulin. The LTQ Orbitrap XL mass spectrometer (Thermo Scientific, Waltham, MA, USA) was operated in positive-ion nanoelectrospray mode. Peptides were diluted 1:1 (v/v) with 70% acetonitrile, 1% formic acid and introduced into the mass spectrometer using metal-coated borosilicate capillaries and a nanospray probe (Proxeon, West Palm Beach, FL, USA). Parameters for acquisition in MS mode were as follows: nanospray source, mass range 400–1000 m/z, Fourier-transform MS (Orbitrap), profile data collection, 60,000 resolution, 1 microscan, 50 ms maximum inject time, automatic gain control of 500,000. Peptide fragmentation MS/MS data were collected in HCD (high energy collision induced fragmentation) mode with the following parameters: mass range 100–1500 m/z, profile data collection, 15,000 resolution, 3 microscans, 500 ms maximum inject time, automatic gain control of 50,000. Data were examined using xcalibur V2.1.0.1139.

## Electronic supplementary material

Supplementary Information
