# Supplementary material for: Controlled modification of biomolecules by ultrashort laser pulses in polar liquids
Source: Sci Rep. 2017 Jul 17;7:5550. doi: 10.1038/s41598-017-05761-8 (PMC5514113; doi:10.1038/s41598-017-05761-8)
Supplement: Supplementary file 1 — Supplementary Information [file 41598_2017_5761_MOESM1_ESM.doc]

Supplementary Information for

Controlled modification of biomolecules by ultrashort laser pulses in polar liquids

Vitaly Gruzdev, Dmitry Korkin, Brian P. Mooney, Jesper F. Havelund, Ian Max Møller, Jay J. Thelen

correspondence to: [gruzdevv@missouri.edu](mailto:gruzdevv@missouri.edu) and [thelenj@missouri.edu](mailto:thelenj@missouri.edu)

**This PDF file includes:**

1. **Materials: HSA peptide standard composition 3**

Table S1. Composition of HSA peptide standard

1. **Setup: Laser and spectroscopy setup and parameters: 4**

Table S2. Laser parameters at three wavelengths utilized in the experiments.

Table S3. Parameters of the spectrometer.

Fig. S1. Scheme of the setup for optical-spectroscopy measurements and laser treatment of the biomolecules.

Fig. S2. Spectra of the laser radiation and a transmittance spectrum of cuvettes.

Fig. S3. Extra details of the experimental setup.

1. **Extra laser effects: 9**

Fig. S4. Nonlinear laser effects at high intensity.

Fig. S5. Spectroscopy characterization of the nonlinear laser effects.

Fig. S6. Characterization of laser-spectrum broadening.

1. **Mass spectroscopy: devices, parameters, and procedures: 12**

Supplementary text.

Fig. S7. MS spectra of control and laser-irradiated peptide.

1. **Extra data from the optical spectroscopy: 13**

Supplementary text.

Fig. S8. Optical transmittance spectra of pure solvents and the test peptide before laser treatment.

Fig. S9. Optical transmittance spectra of peptide and ACN:water solvent.

Fig. S10. Modification of optical transmittance spectrum of the ACN:water mixture (98:2% v/v) with different number of laser pulses.

Fig. S11. Modification of optical transmittance spectra of pure methanol with different number of laser pulses.

Fig. S12. Influence of solvent on laser-induced modifications of absorption spectrum of the test peptide.

Fig. S13. Dependence of laser-induced variations of transmittance spectrum of ACN:water mixture (98:2 % v/v) on laser wavelength.

Fig. S14. Simultaneous influence of peak laser intensity and number of laser pulses.

Fig. S15. Modification of absorption band of the test peptide in water.

1. **Evaluation of harmonics of some vibration modes in solvents 25**

Supplementary text

Table S4. Wavelength of harmonics of O-H stretching mode of water

Table S5. Wavelength of harmonics of O-H stretching mode of methanol

Fig. S 16. Absorption spectra of water, methanol, and acetonitrile around 386 nm.

1. **Supplemental data from mass spectrometry: 27**

7.1. Solvent-dependent modification of the test peptide

Fig. S17. Mass spectra of the test peptide treated in water.

Fig. S18. Mass spectra of the test peptide treated in pure methanol.

Fig. S19. Mass spectra of the test peptide treated in a methanol:water mixture.

Fig. S20. Mass spectra of the test peptide treated in ACN:water (98:2 % v/v).

Fig. S21. Mass spectra of the test peptide in pure ACN treated by laser pulses at 386 nm and 257 nm.

7.2. Peptide modification is controlled by varying water content Fig. S22. Modification of the test peptide relative to water content.

7.3. Observed modifications are formylation on primary amines and methionine oxidation Fig. S23. Orbitrap HCD MS/MS spectrum of the +56 Da modified test peptide.

Fig. S24. MALDI TOF MS and TOF-TOF MS/MS spectra of trypsin-digested +56 Da modified test peptide.

Fig. S25. High resolution ESI-Orbitrap FTMS spectrum of the test peptide in ACN:water (98:2 % v/v).

Table S6. Possible elemental composition of mass differences.

Table S7. Atomic groups that are attached to the test peptide by the laser treatment.

7.4. Solvent contributions to formylation and oxidation: sources of carbon and oxygen Fig. S26. Mass spectra of the test peptide treated in isotope-labeled methanol:water.

Fig. S27. Mass spectra of the test peptide treated in isotope-labeled ACN:water.

Fig. S28. High-resolution mass spectrum of the test peptide treated in isotope-labeled ACN:water.

Fig. S29. Mass spectrum of the test peptide modified in a mixture of unlabeled ACN:H_2_^18^O (98:2 % v/v).

7.5. Influence of air on formylation can be suppressed by bubbling argon through solution Fig. S30. Modification of the test peptide following argon sparging.

7.6. Free lysine (Lys) is not modified upon laser irradiation Fig. S31. Mass spectra of laser-irradiated free Lys.

7.7. Laser-induced formylation of human serum albumin (HSA) peptidesFig. S32. Mass spectra of the laser-modified peptides of HSA seven-peptide standard.

7.8. Laser-induced formylation of insulin Fig. S33. Mass spectra of insulin modified in ACN:water (98:2 % v/v).

Fig. S34. FTIR spectra of laser-treated CAN:water mixture (98:2 % v/v).

Fig. S35. Mass spectra of the test peptide treated at different values of laser wavelength.

1. Materials: HSA peptide standard composition

Table S1. Composition of HSA peptide standard

Synthetic peptides #1 and #5 below have carbamidomethylated cysteines (+ 57 Da). For all the peptides, the C-terminus is the free acid and the N-terminus is unmodified (-NH_2_).

| # | HSA peptide sequence and chemical composition | Mol. Weight [g/ mol] | Chemical purity | Tolerance |
| --- | --- | --- | --- | --- |
| 1 | AAFTE**CC**QAADK  C_55_H_86_O_21_N_16_S_2_ | 1371.52 | ≥ 97% | ± 10% |
| 2 | YLYEIAR  C_44_H_66_O_12_N_10_ | 927.05 | ≥ 97% | ± 10% |
| 3 | LVNEVTEFAK  C_52_H_84_O_17_N_12_ | 1149.29 | ≥ 97% | ± 10% |
| 4 | KVPQVSTPTLVEVSR  C_72_H_126_O_23_N_20_ | 1639.92 | ≥ 97% | ± 10% |
| 5 | RP**C**FSALEVDETYVPK  C_85_H_131_O_27_N_21_S | 1911.18 | ≥ 97% | ± 10% |
| 6 | AVMDDFAAFVEK  C_61_H_91_O_19_N_13_S | 1342.54 | ≥ 97% | ± 10% |
| 7 | HPYFYAPELLFFAK  C_90_H_119_O_19_N_17_ | 1743.01 | ≥ 97% | ± 10% |

1. **Setup: Laser and spectroscopy setup and parameters**

Table S2.

Laser parameters at three wavelengths utilized in the experiments.

| Central wavelength, nm | Full width of laser spectrum (at half maximum), nm | Range of variations of average pulse energy, μJ | Standard deviation of pulse energy, % |
| --- | --- | --- | --- |
| 772.00 ± 1.10 | 8.65 ± 0.09 (e.g., Fig. S2A) | 1.0 – 1500.00 | 0.8 |
| 386.00 ± 0.14 | 7.94 ± 0.08 (e.g., Fig. S2B) | 9.51 – 315.80 | 2.0 |
| 257.23 ± 0.12 | 4.18 ± 0.07 | 3.51 – 15.05 | 2.5 |

Table S3.

Parameters of the spectrometer. A spectrometer model AvaSpec-USB2-RM was employed for the transmittance optical spectroscopy.

| Channel number | Wavelength range, nm | Detector type | Integration time, ms | Number of spectra taken for averaging |
| --- | --- | --- | --- | --- |
| 1 (UV) | 185 - 756 | TCD1304 | 7.0 | 500 |
| 2 (VIS) | 596 – 1100 | TCD1304 | 3.7 | 1000 |
| 3 (NIR) | 937 – 2607 | Ham92xx | 3.9 | 1000 |


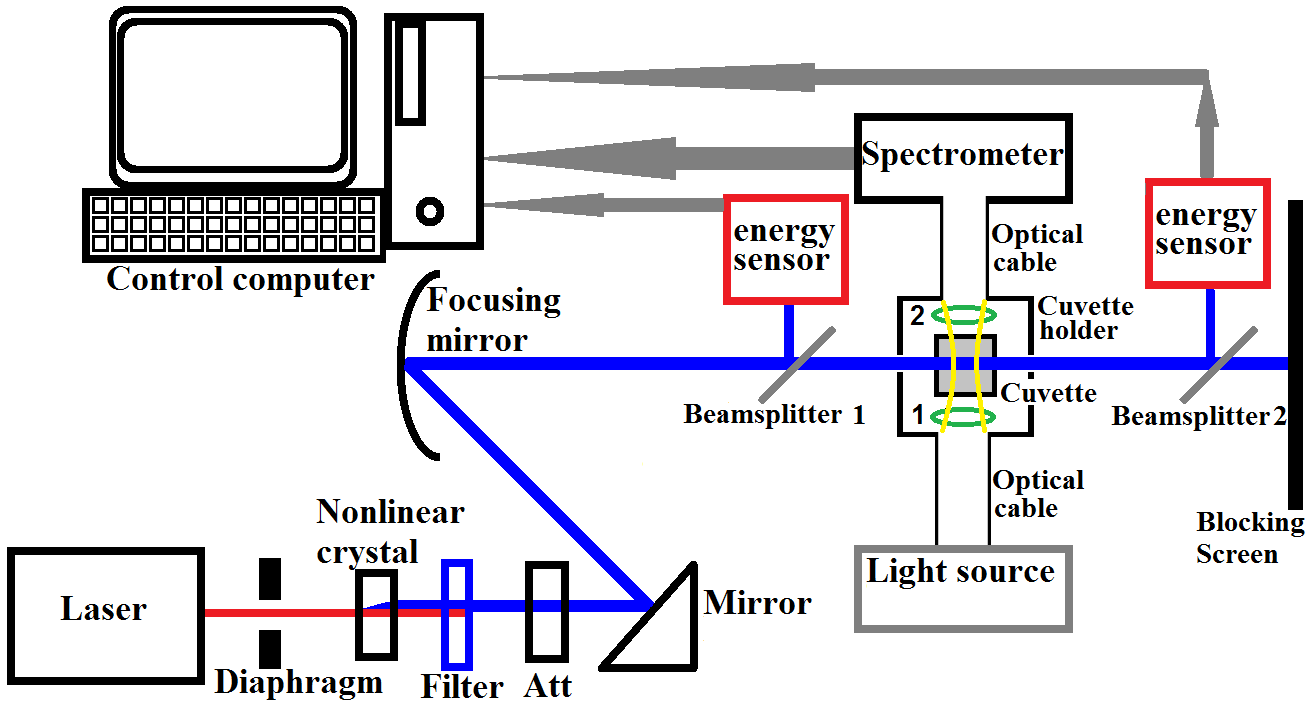


Fig. S1.

Scheme of the setup for optical-spectroscopy measurements and laser treatment of the biomolecules. Laser beam (wavelength 772 nm) passes through a diaphragm, a nonlinear crystal (for frequency conversion), a filter (to block residual light at 772 nm), optical attenuator (Att) to adjust pulse energy, a bending mirror, and is forwarded to a focusing mirror (f = 500 mm) with focal point behind the rear side of cuvette. The focused beam is further forwarded to beam splitter 1 (about 11% of incident energy is reflected towards an energy sensor), cuvette with a liquid, beam splitter 2 (reflects about 11% of incident energy to an energy sensor), and finishes the path at a blocking screen. Light of the spectroscopy sources is delivered by an optical cable and collimated by lens 1 attached to the output end of the cable (Fig. S3). Light transmitted through the cuvette is collected by lens 2 and is coupled to an optical cable attached to a spectrometer. Signals from sensors and the spectrometer are processed by a computer using manufacturer’s software. Pulse width is measured by replacing the energy sensor with an autocorrelator at beam splitter 1.


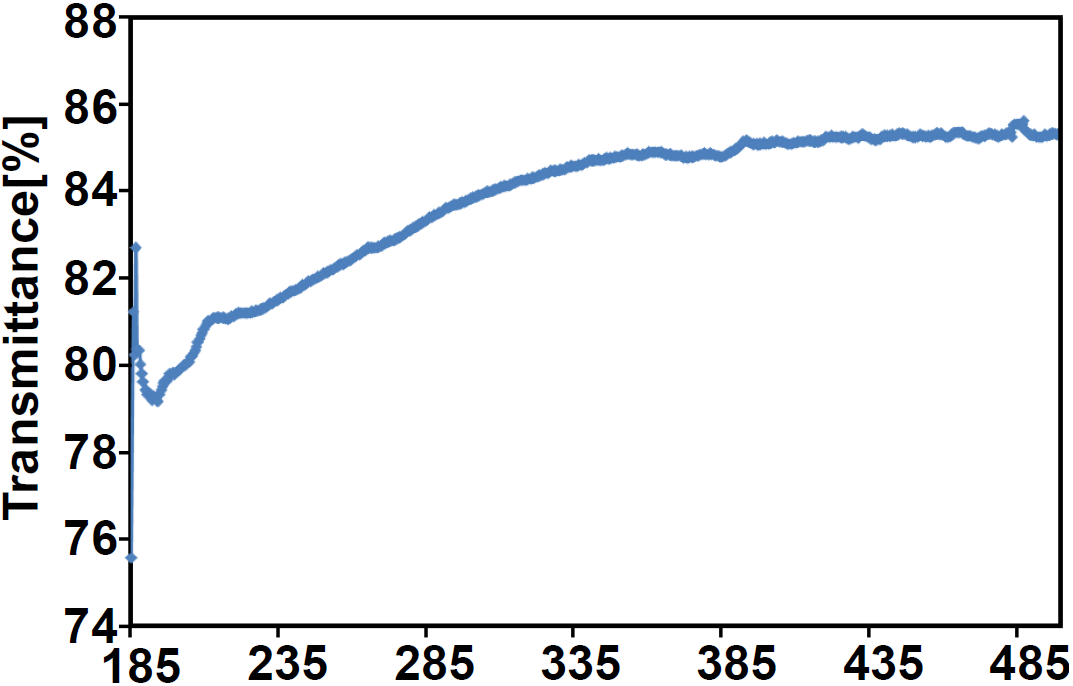


C

Fig. S2.

Spectra of the fundamental and second harmonic of laser radiation. The fundamental harmonic (A, central wavelength 772 nm) and the second harmonic (B, central wavelength 386 nm) of laser radiation utilized for biomolecule modification show different amplitudes of the spectral lines because the fundamental harmonic was detected by VIS channel of the spectrometer while the second harmonic was detected by UV channel. Integration time that controls the signal amplitude was different for those channels. (C) - Representative transmittance spectrum of a quartz cuvette (Starnacell, USA; part number 3-Q-10) utilized in the experiments. This transmittance was measured with air as reference and has not normalized to compensate for the contribution from reflection and wavelength dependence on refraction.


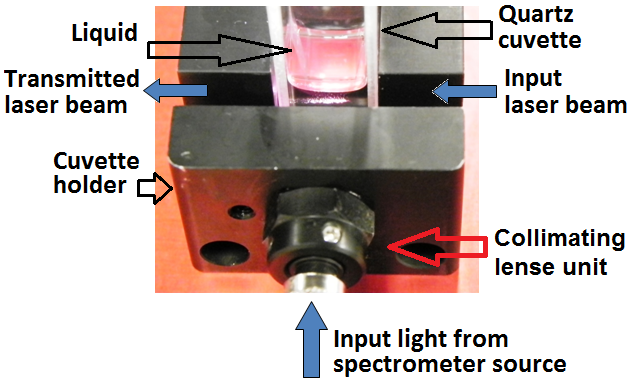
 A


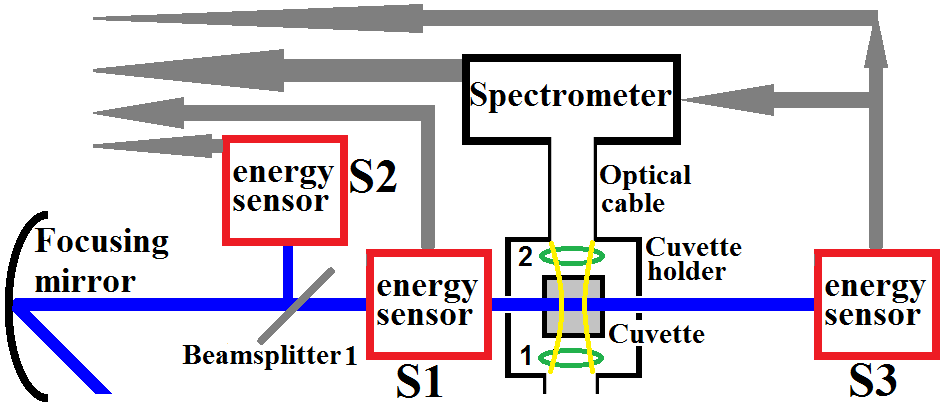
 B

Fig. S3.

Additional details of the experimental setup. (A) A photograph of the cuvette holder without top cover with directions of propagation of light beams from the laser and from the spectrometer light source. (B) Modification of the basic experimental setup (Fig. S1) utilized for measurement of the transmittance at the front cuvette wall at wavelength 386 nm and the amount of spectrum broadening due to nonlinear propagation effects (see Section 3).

1. Extra laser effects

The extra laser effects observed in experiments include various nonlinear interactions of femtosecond laser pulses with liquids utilized as solvents. Those effects were not studied in details because that effort significantly departs from the objectives and scope of this publication. However, some of the effects were briefly characterized from the viewpoint of their influence on the peptide. Of special interest was the generation of micro-bubbles within a laser track; and spectrum broadening. Both effects have threshold-type dependence on laser intensity: they do not appear if laser intensity is below certain level referred to as threshold. Above the threshold, dependence of those effects on laser intensity can be either nonlinear (e.g., width of broadened spectral line – Fig. S5 B) or linear (e. g., height of the bubble-induced scattering peak – Fig. 5 C).


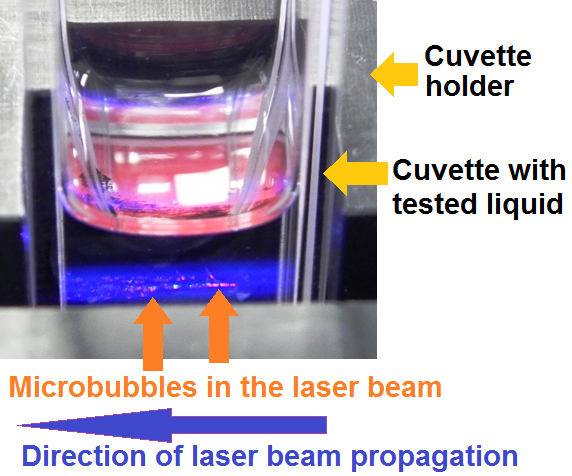

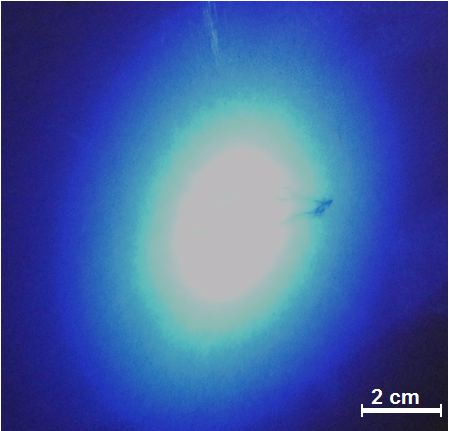


A B

Fig. S4.

Nonlinear laser effects at high intensity. A picture of micro-bubble generation in the ACN:water (98:2% v/v) mixture (A) and transmitted laser spot on a screen installed 30 cm behind the rear side of the cuvette (B) taken during exposure to a train of laser pulses at average pulse-energy 34.5 μJ and laser-spot diameter 3 mm at front wall of the cuvette (fluence 488.1 μJ/cm^2^; peak intensity 3.25 GW/cm^2^). The reduced beam diameter was specifically utilized for this experiment on spectrum broadening. The majority of the experiments on peptide modification were done at laser-beam diameter of about 6 mm. With the total cross section of 10 mm by10 mm of the liquid in a standard 10-mm spectrometer cuvette, the 6-mm-diameter laser beam reached a very significant part of the liquid. Also, the bluish track visible in Figure S4A is due to the nonlinear broadening of pulse spectrum during propagation in the liquids. The broadening results in generation of some violet and blue light that becomes visible to the human eye. However, this means that only the small and most intensive central part of the laser beam becomes visualized due to spectrum broadening. The rest of the laser beam is not visible by eye.


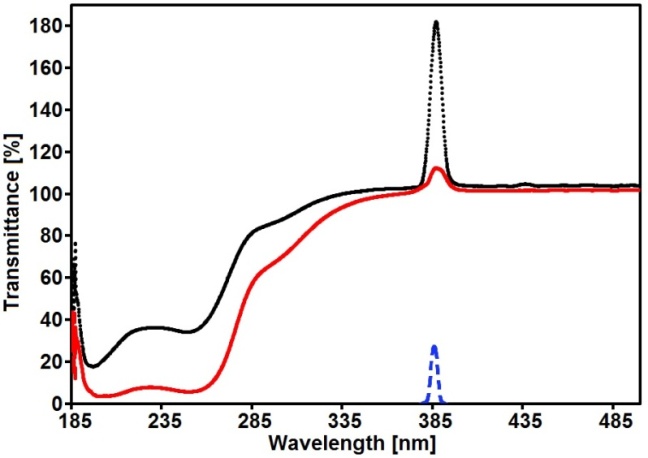

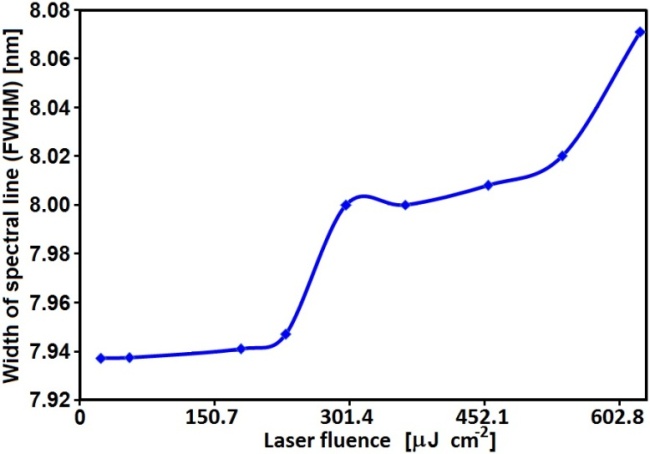


A B


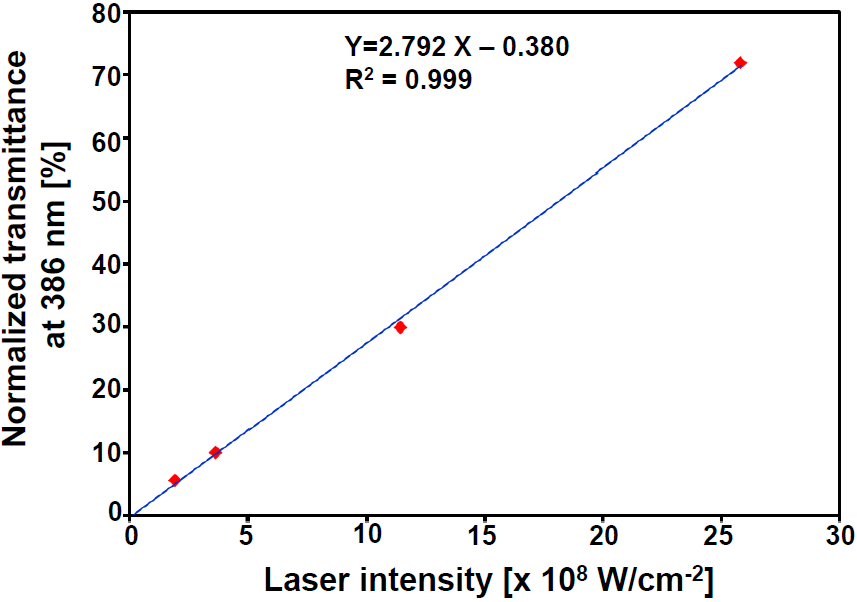


C

Fig. S5

Spectroscopy characterization of the nonlinear laser effects. (A) Transmittance spectra acquired during exposure of ACN:water (98:2 % v/v) mixture to 60000 laser pulses at peak intensity 3.25·10^9^ W/cm^2^ (black curve) and 2.54·10^9^ W/cm^2^ (red curve). The spectrum contains a narrow peak centered at the laser wavelength and associated with scattering laser beam by micro-bubbles generated during liquid decomposition by laser pulses. Blue dashed line depicts spectrum of laser pulses. (B) Dependence of full width at half maximum of transmitted spectrum of laser light on laser fluence (laser-spot diameter was 6.5 mm as measured at the front wall of the cuvette). (C) The peak transmittance at laser wavelength (386 nm) after subtraction of the scattering-free background of 100% (see panel A) plotted as a function of peak laser intensity. Transmittance at wavelength 386 nm was taken for ACN:water (98:2 % v/v). Shown are the values extracted from the transmittance spectra similar to panel A (red squares), trend line (blue solid), trend equation, and interpolation parameter. Threshold of bubble generation is evaluated from the trend equation by assuming *y* = 0.0. We emphasize that panel C represents the peak of the narrow scattering line of transmittance spectra for solvents only, and it is not attributed to the threshold of the test-peptide modification discussed in the main text.


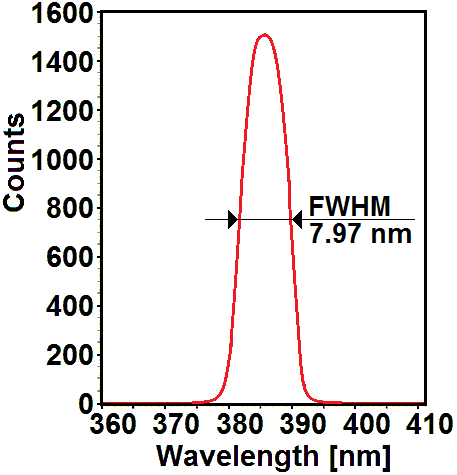

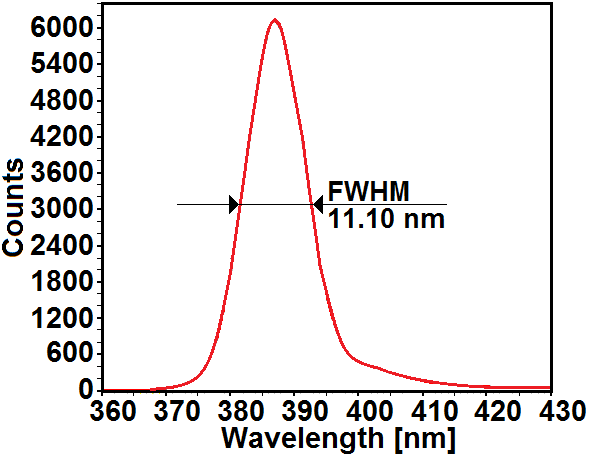


**A B**

Fig. S6.

Characterization of laser-spectrum broadening. Spectrum of laser pulses taken right next to the front wall of a cuvette (A) with the ACN:water (98:2 % v/v) mixture and just behind the rear wall of the cuvette (B) at peak intensity 2.49·10^11^ W/cm^2^ (fluence 37.42 mJ/cm^2^; pulse energy 293.9 μJ).

1. Mass spectroscopy: devices, parameters, and procedures

An initial comparison of control and laser-irradiated peptide spectra (Fig. S7) demonstrated that a) the control (non-irradiated) peptide spectrum contained a prominent peak of 1620.8 Da corresponding to the unmodified protonated peptide and a less abundant peak of 1636.8 Da, +16 Da (Fig. S7A), and b) the peptide was almost completely modified by laser irradiation producing prominent peaks at 1676.9 Da (+ 56 Da) and 1692.9 Da (+ 72 Da), leaving <1% (by peak height) of the unmodified form (Fig. S7B).

**
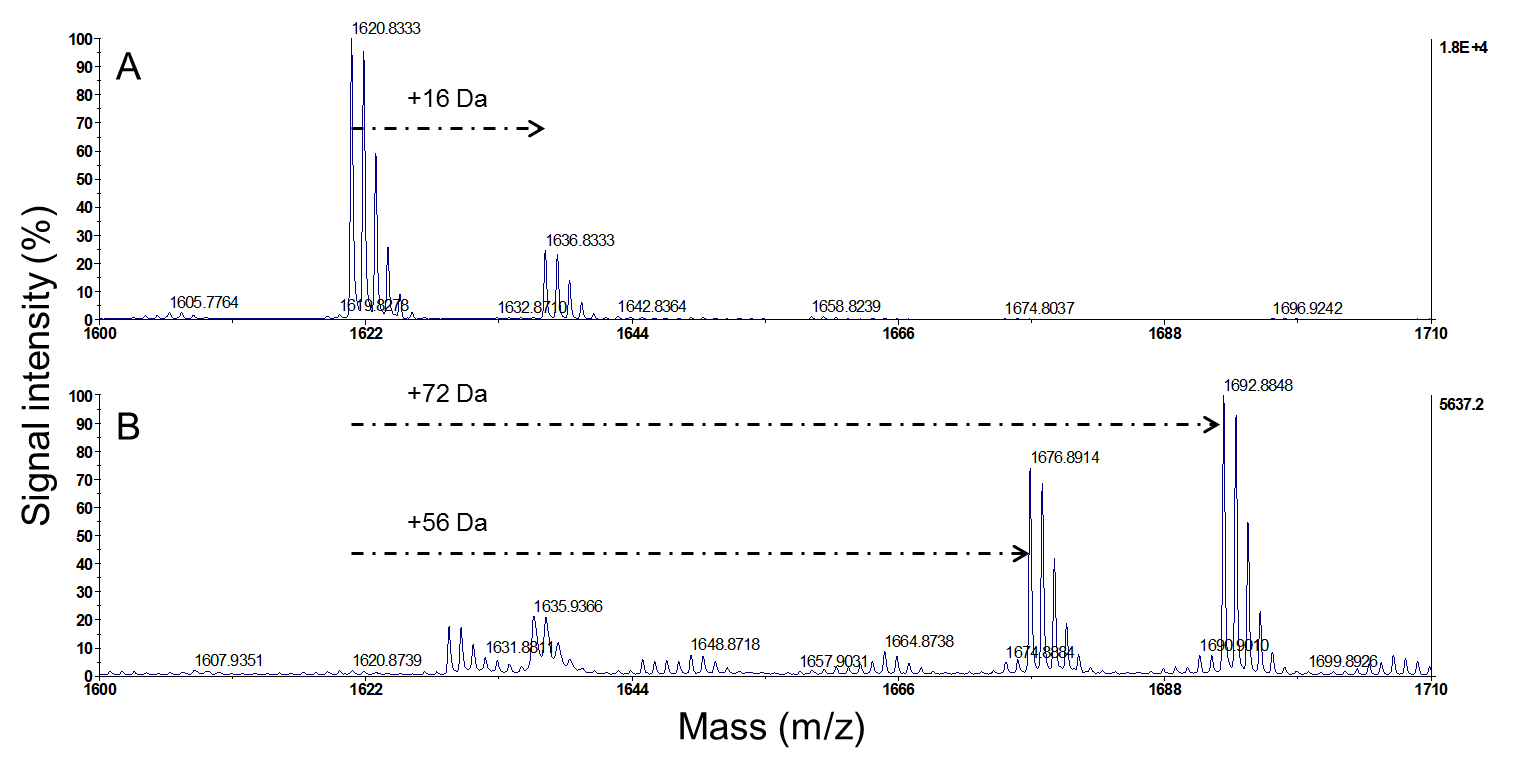
**

Fig. S7.

MS spectra of control and laser-irradiated peptide. (A) MS spectrum of the control peptide in ACN:water (98:2 % v/v) not exposed to laser irradiation. (B) MS spectrum of the laser-irradiated peptide (pulse energy 260.3 μJ; fluence 1.64 mJ/cm^2^; intensity 10.9 GW/cm^2^). Modifications (changes is mass of the peptide) in control and laser-irradiated samples are indicated by dashed arrows.

1. Extra data from the optical spectroscopy

5.1 Transmittance spectra of solvents prior to laser treatment

Transmittance spectra of each solvent were taken prior to laser treatment (Fig. S8) with an empty quartz cuvette as a reference i.e., the interior was filled with air. Some of the transmittance spectra of Figs. S8, S9, and S10 deviate from the usual spectra published in reference books (e.g., Ref. [27]). The physical reason for that deviation is attributed to the fact that the published reference-book spectra are usually taken with water as the reference substance. One can estimate variations of transmittance due to the use of air and water as two reference materials. For simplicity, we assume zero absorption at cuvette-exterior and cuvette-interior interfaces. Then, transmittance *T_i_* at interface *i* (*i* = 1; 2; 3; 4) is evaluated via reflectivity *R_i_* at the same interface:

*T_i_* = 1 – *R_i_, i =* 1, 2, 3, 4


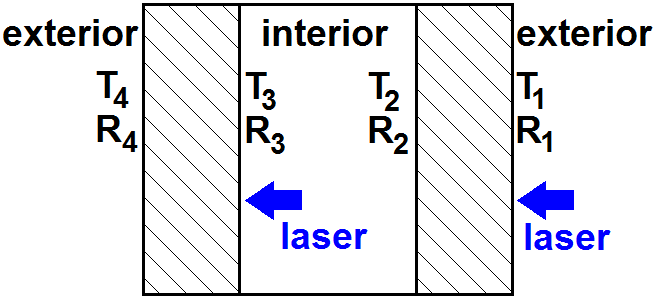


The total transmittance *T* of a cuvette (that is registered by a spectrometer – see Fig. S1) is the product of the transmittances of all the four interfaces. However, note that *T*_1_ = *T*_4_ since exterior medium is air for all experiments. Also, *T*_2_ = *T*_3_. Therefore, the total transmittance is evaluated via *R_i_* as follows

$T=\left(1-R_{1}\right)^{2}\left(1-R_{2}\right)^{2}$.

The material of the cuvette walls was fused quartz. At 386 nm wavelength, refractive index of air can be assumed to be *n_air_* = 1.00; refractive index of water is *n_w_* = 1.34 (e.g., see <https://refractiveindex.info/?shelf=main&book=H2O&page=Hale>); and refractive index of fused quartz is *n_fq_* = 1.47 (see, for example, <https://refractiveindex.info/?shelf=glass&book=fused_silica&page=Malitson>). Reflectivity at each interface can be evaluated by Fresnel formulas for normal incidence of a laser beam (that is the case for our setup) as follows:

$R_{i}=\left(\frac{n_{a}-n_{b}}{n_{a}+n_{b}}\right)^{2}$,

where *n_a_* and *n_b_* are values of refractive index of the two substances making the interface *i*. Summarizing all those relation, one can express the ratio of the total transmittance *T_w_* obtained with water as reference to the total transmittance *T_air_* obtained with empty cuvette as reference:

$\frac{T_{w}}{T_{air}}=\left(\frac{1-R_{2w}}{1-R_{2air}}\right)^{2}=\left(\frac{1-\left(\frac{n_{fq}-n_{w}}{n_{fq}+n_{w}}\right)^{2}}{1-\left(\frac{n_{fq}-n_{air}}{n_{fq}+n_{air}}\right)^{2}}\right)^{2}$.

Substituting the values of refractive index shown above, one evaluates the ratios to be 1.072, i. e., the difference between the reference-book spectra and the transmittance spectra presented below is about 7.2%. The decrease in the transmittance with increase of wavelength is due to the refractivity, i.e., dependence of refractive index of water on wavelength. Although it is possible to normalize the transmittance spectra by introducing a correction factor to compensate for the influence of refractive index of a reference substance, we avoid unnecessary normalization of the directly acquired data to keep the reported results more reproducible.

The strongest absorption band of water was associated with H-O bonds *(27)* and expanded partly beyond the working range of the spectrometer. The triple C-N bond of ACN (Fig. S8, S9) showed a narrow absorption band centered near 190 nm *(27)*. C-O bonds of methanol produced a broad absorption band centered at 200 nm (Fig. S8). The absorption band of the test peptide associated with peptide bonds of the backbone *(27)* was centered near 205 nm and was shifted to longer wavelengths and broadened compared to published spectroscopy data *(27)* due to significant peptide-solvent interactions at room temperature. Its long-wavelength edge merged with absorption bands of the side chains in the range 230-290 nm (Fig. S9A,B).

The strong absorption bands of methanol and ACN-water mixture partly overlapped with the absorption band of the test peptide (Fig. S9). Moreover, exposure to laser pulses significantly expanded the original absorption bands of the solvents (except water) towards longer wavelengths (see Figs. S9-S15). Those facts prevented attempts of directly affecting the peptide bonds by laser pulses in the solvents since laser light would be significantly absorbed by the solvents before reaching the peptide. However, the absorption bands of side chains could be affected by single-photon absorption of the laser pulses with tripled frequency, i.e., at 257 nm.

All liquids and the peptide exhibited shallow absorption bands in the range 365 - 390 nm. They were attributed to solvent-to-solvent and solvent-to-peptide H-bonds. Except pure water, those shallow bands overlapped very well with spectrum of the second harmonic of laser pulses (Fig. S8-S15). Some estimates are given in Section 7 regarding the H-bonds and their spectral line.

Direct detection of laser modifications of the test peptide by differential transmittance spectroscopy (i.e., by using non-treated sample as a reference) was not feasible in most of the solvents. Low concentration of the test peptide was not favorable for detection of its contribution to the total laser-induced variations of transmittance of the liquid samples because modifications of absorption bands of the peptide were significantly masked by laser-induced modifications of all solvents except water (Figs. S10 through S15).

5.2 Laser treatment of 100% ACN and mixture of ACN and water (98:2 % v/v)

Laser treatment of the ACN:water mixture (Fig. S10) produced the same results as pure ACN and resulted in an increase of absorption at the center of the initial absorption band (205 nm) as well as formation of broad side absorption bands. Of these, the most prominent were the bands centered near 260 nm and 305 nm (Fig. S10). The shallow absorption band centered at 375 nm (Fig. S10) received a pronounced modification by the laser treatment.

5.3 Laser treatment in 100% methanol and methanol:water (98:2 % v/v)

Laser treatment of pure methanol and methanol:water (98:2 % v/v) solvents resulted in slight modification of the original absorption band centered at 207 nm (Fig. S11). Remarkably, no change of the weak absorption band centered at 375 nm was produced by laser irradiation at 386 nm. Fig. S11 demonstrates significant accumulation of laser action with increase of number of laser pulses from 60,000 up to 900,000.

5.4 Laser treatment of pure water

Water was the most resistant to laser action and demonstrated no change of transmittance spectrum within the studied spectral range. Multiple experiments with exposure time as long as 900 seconds did not show any modification of transmittance of water beyond regular error range by laser pulses at peak intensity 2.0-200.0 GW/cm^2^ at wavelength 386 nm.

5.5. Laser treatment of the test peptide: influence of solvent

The transmittance spectrum of the test peptide taken prior to laser treatment was significantly influenced by solvents (Fig. S12). Laser action directly affected the shallow absorption bands in the range 365-390 nm, but also formed sub-bands in the range 235-285 nm (Fig. S12).

5.6 Laser treatment of ACN:water mixture: influence of laser wavelength and intensity

Laser modification of solvents was strongly influenced by laser parameters. The fundamental laser wavelength (772 nm, corresponding photon energy 1.56 eV) was far beyond any significant absorption band. Therefore, the only mechanism of solvent modification could be attributed to two-photon (equivalent wavelength 386 nm) and three-photon (equivalent wavelength 257 nm) absorption the rates of which depended on laser intensity in a nonlinear way. The transmittance spectra showed no modifications of the solvent near 386 nm while significant modifications were observed in the range 190-315 nm (Figs. S13 and S14). The most prominent laser-induced peaks of absorption at 193, 258, and 295 nm merged and formed a broad band at increased laser intensity and/or exposure time (Figs. S13, S14). The modifications exhibited a threshold-type behavior when produced by laser light at wavelength 772 nm (Fig. S14). Laser action at the second-harmonic wavelength (386 nm, photon energy 3.12 eV) altered the weak absorption band in the range 380-390 nm and also induced significant absorption at 195 and 255 nm (Fig. S13). At 772 and 386 nm, some modified absorption peaks were located at the wavelengths of one- and two-photon absorption (e.g., 386 nm and 193 nm for laser wavelength 386 nm). However, other modifications of absorption took place at wavelengths not associated with direct linear or nonlinear absorption (e.g., at 295 nm) signaling possible involvement of intra-molecular vibration-energy transfer *(14)* in the observed modifications of that solvent.

5.7 Laser modification of the test peptide in water

The UV absorption band of water did not significantly overlap with that of the peptide (Fig. S8) and was not modified by the laser at 386 and 772 nm. For that reason, water was the only solvent favorable for direct detection of laser-induced modifications of the peptide by variations of its transmittance spectrum (Fig. S15). The most prominent modification was in the range 240-285 nm associated with side chains of the peptide.


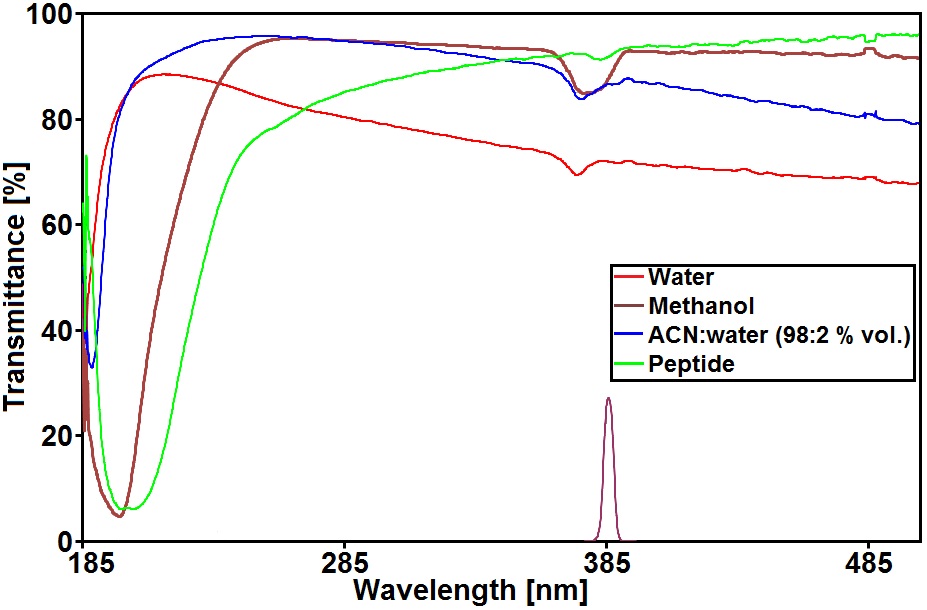


Fig. S8.

Optical transmittance spectra of pure solvents and the test peptide before laser treatment. Spectrum of the test peptide was taken in de-ionized water with water as a reference. An empty quartz cuvette was the reference for measurements of transmittance of water, methanol, and ACN:water mixture. Water was utilized as reference for measurements of transmittance of the test peptide. Spectrum of laser pulses is at the bottom.


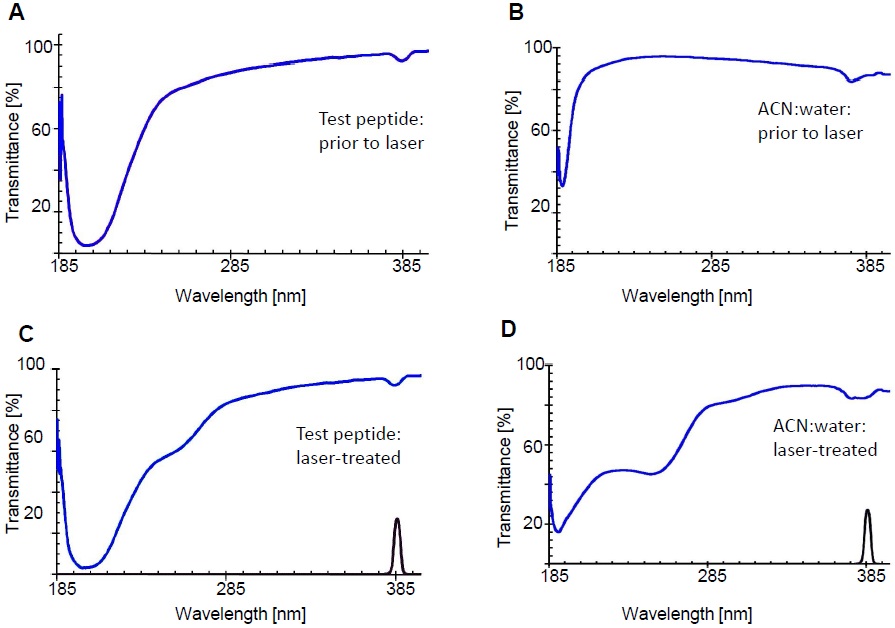


Fig. S9.

Optical transmittance spectra of peptide and ACN:water solvent. (A) Transmittance spectrum of the test peptide in the ACN:water (98:2; % v/v) solvent and (B) the solvent alone both prior to laser treatment demonstrate strong absorption band in the far-UV domain and a weak absorption band centered around 385 nm. (C) Transmittance spectrum of the peptide in the solvent and (D) of the solvent alone both after treatment with 180000 laser pulses (wavelength 386 nm; pulse energy 293.9 μJ; fluence 37.42 mJ/cm^2^; intensity 249.0 GW/cm^2^) show expanded absorption bands in the far UV domain with almost no modification of the shallow absorption band at 385 nm. The spectrum of laser pulses is shown at the bottom line of parts C and D. For the peptide spectra (A) and (C), untreated solvent was utilized as control. For the solvent spectra (B) and (D), an empty cuvette was utilized as reference.


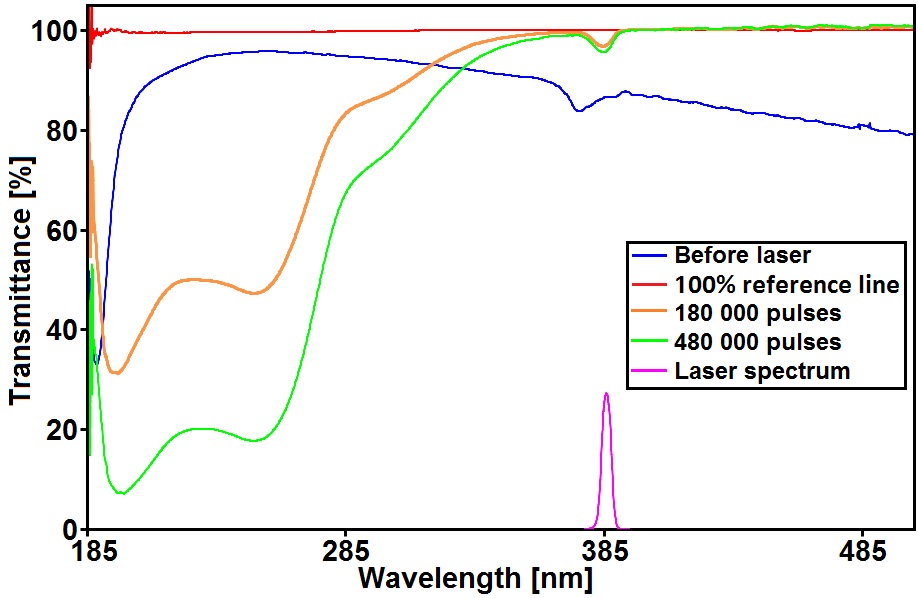


Fig. S10.

Modification of the optical transmittance spectrum of the ACN:water mixture (98:2% v/v) with different number of laser pulses. Results are shown in the range 185-505 nm after treatment with variable number of laser pulses at fixed wavelength 386 nm; pulse energy 293.9 μJ; fluence 37.42 mJ/cm^2^; and intensity 249.0 GW/cm^2^. Blue line depicts the spectrum taken prior to laser treatment with empty cuvette as reference. The other spectra are taken with untreated liquid as a reference using differential-spectroscopy approach.


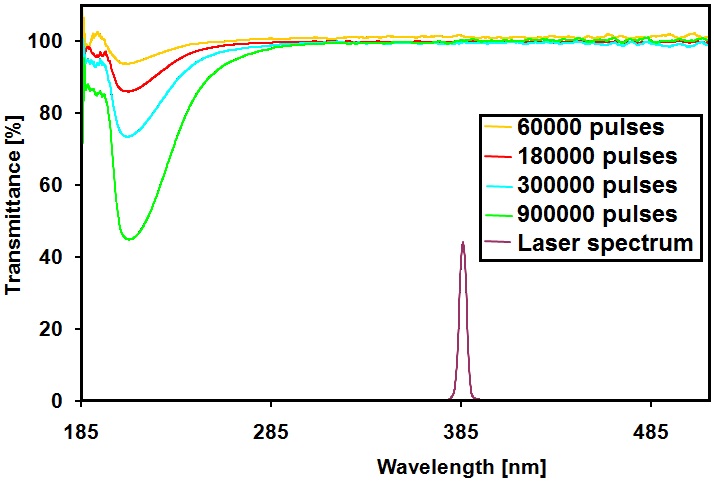


Fig. S11.

Modification of the optical transmittance spectrum of pure methanol with different number of laser pulses. The results are shown in the range 185-585 nm after treatment at laser wavelength 386 nm; pulse energy 314.4 μJ; fluence 4.45 mJ/cm^2^; and intensity 296.5 GW/cm^2^. The four transmittance spectra were taken with the untreated solvent as a reference and show laser-induced deviations from the reference 100% line. The spectrum of the laser radiation is shown with violet curve at the bottom of the figure.


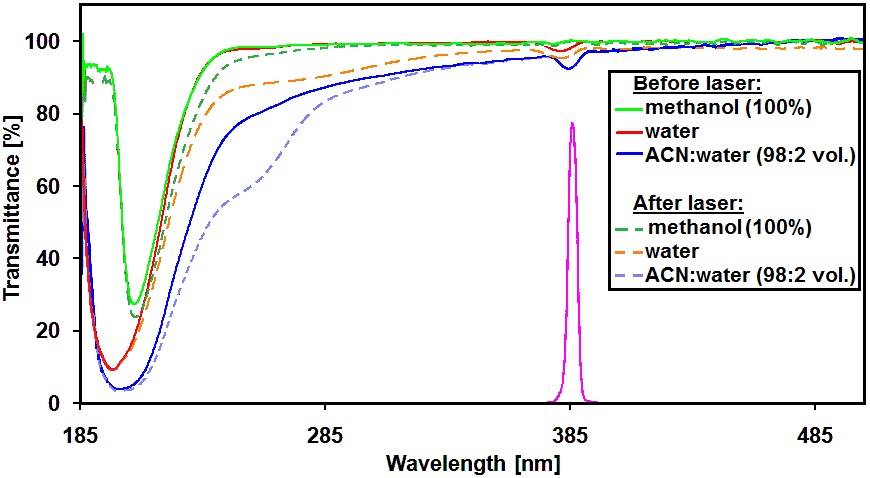


Fig. S12.

Influence of solvent on laser-induced modifications of the absorption spectrum of the test peptide. Fixed exposure to 180,000 laser pulses was done at central wavelength 386 nm. Solid lines depict initial absorption of the peptide in water (red), methanol (green); and ACN:water (98:2% v/v) mixture (blue). Dashed lines depict absorption of the peptide after modification by 360 000 laser pulses: in water (orange line; pulse energy 303.8 μJ; fluence 38.68 mJ/cm^2^; intensity 258 GW/cm^2^); methanol (light green; pulse energy 307.6 μJ; fluence 39.17 mJ/cm^2^; intensity 261 GW/cm^2^), and ACN:water mixture (light blue; pulse energy 293.9 μJ; fluence 37.42 mJ/cm^2^; intensity 249 GW/cm^2^). The spectrum of the laser pulses is depicted by the violet line at the bottom of the figure.


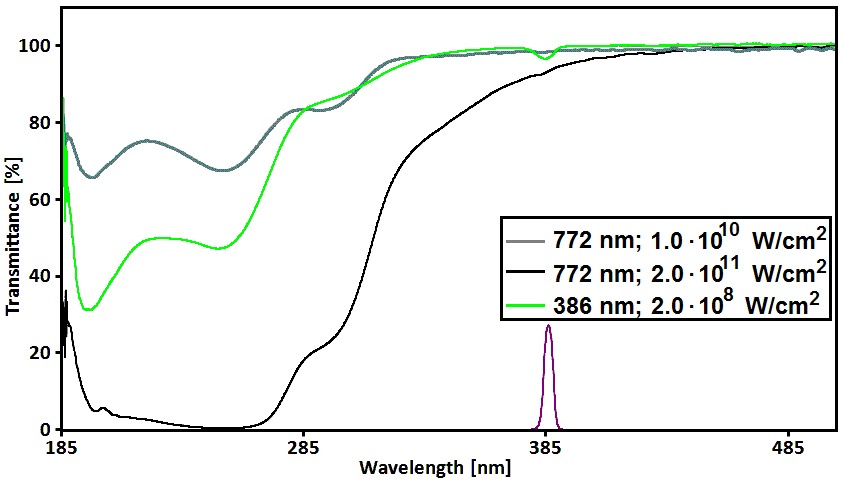


Fig. S13.

Dependence of laser-induced variations of transmittance spectrum of ACN:water mixture (98:2 % v/v) on laser wavelength. Average energy of laser pulses was 1.29 mJ at wavelength 772 nm (grey and black curves), and 0.29 mJ at wavelength 386 nm (green curve). Untreated solvent was utilized as a reference for all the depicted spectra. The spectrum of the laser pulses is depicted by the violet line at the bottom of the figure.


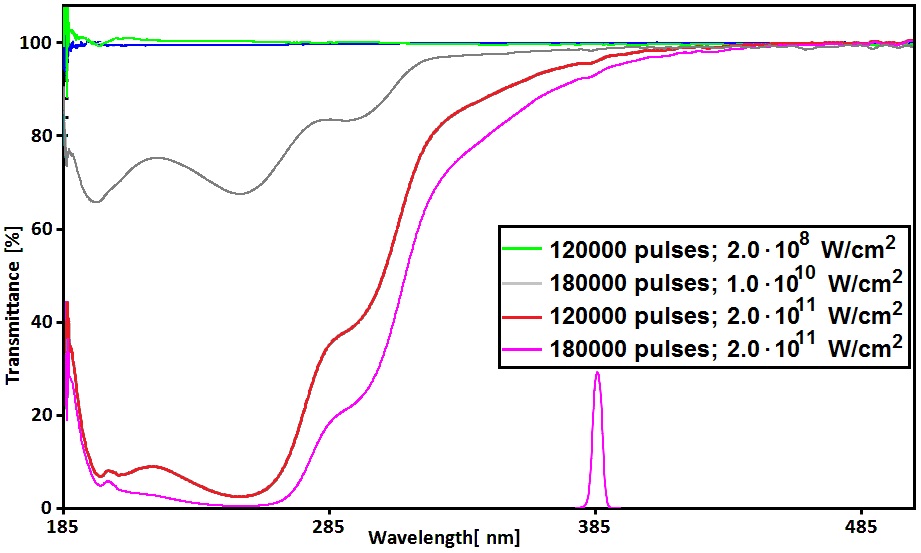


Fig. S14.

Simultaneous influence of peak laser intensity and number of laser pulses. Dependence of laser-induced variations of transmittance spectrum of ACN:water (98:2 % v/v) mixture on peak intensity and number of laser pulses is shown for laser wavelength 772 nm. Untreated solvent was utilized as a reference. The spectrum of laser pulses is shown by the violet line at the bottom of the figure.


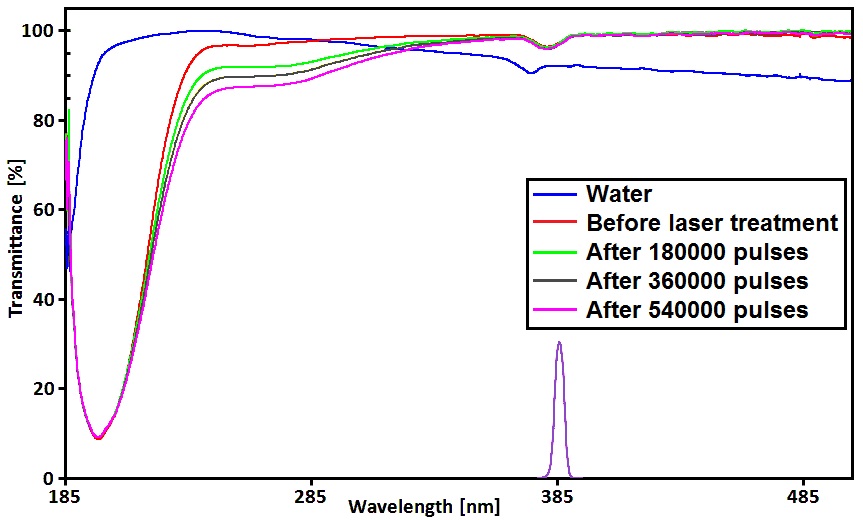


Fig. S15.

Modification of absorption band of the test peptide in water. 360,000 femtosecond laser pulses were applied at peak intensity 2.58·10^11^ W/cm^2^ (pulse energy 303.8 μJ; fluence 38.68 mJ/cm^2^) and wavelength 386 nm. Blue line depicts the transmittance spectrum of water; the other lines depict laser-induced transmittance of the peptide obtained by different exposures. The spectrum of laser pulses is shown by the violet line at the bottom of the figure.

1. Evaluation of harmonics of some vibration modes in solvents

The weak absorption bands in the range 365-390 nm observed in the optical transmittance spectra of solvents are attributed to harmonics of the fundamental stretching mode of O-H bond. This statement is supported by the following two tables compiled using the data of Refs. [14-16] for water (Table S4) and the data from Refs. [17-19] for methanol.

**Table S4.** Harmonics of O-H stretching mode of water and the fundamental scissors mode for water molecule. Wavelength of the O-H stretching mode harmonics is evaluated using equation (22) from Ref [15]: *ν_n_* = *n* (3620 – 63 *n*) [1/cm]. Wavelength of the scissor mode is 1645 1/cm following the data of Ref. [16].

| Harmonic order; O-H stretching mode | Harmonic order; scissor mode | Wave number [1/cm] according to Eq. (22) of Ref. [15] | Wavelength [nm] |
| --- | --- | --- | --- |
| 8 | 0 | 24928 | 401 |
| 8 | 1 | 26573 | 376 |
|  |  |  |  |

**Table S5.** Harmonics of O-H stretching mode of methanol. Wavelength of the O-H stretching harmonics is evaluated using the data from Refs. [17-19]. Wave number of O-H stretching harmonics is evaluated by the relation *ν_n_* = *n* (3404.6 – 38.3 (*n* + 1)) [1/cm]

| Harmonic order; O-H stretching mode [35, 36] | Harmonic order; CH_3_ rocking mode (1115 1/cm); liquid [19] | Harmonic order; C-O stretching mode (1030 1/cm); liquid; [19] | Wave number [1/cm] | Wavelength [nm] |
| --- | --- | --- | --- | --- |
| 8 | 0 | 0 | 24479.2 | 408.5 |
| 8 | 1 | 0 | 25594.2 | 390.7 |
| 8 | 2 | 0 | 26709.2 | 374.4 |
| 8 | 0 | 1 | 25509.2 | 392.0 |
| 8 | 0 | 2 | 26539.2 | 376.8 |
| 8 | 1 | 1 | 26624.2 | 375.6 |

The data of Tables S4 and S5 and the measured absorption spectra (Fig. S16) suggest that the wavelength of 8-th harmonic of O-H stretching mode and the fundamental harmonic of the scissor mode of water make the closest fit to the peak wavelength of absorption in the range 365-390 nm. The 8-th harmonic of O-H stretching mode of methanol added to the second harmonic of C-O stretching mode make the best fit for the position of the peak of absorption in the range 365-390 nm.

Note that the data inTables S4 and S5 may deviate slightly from the experimental data due to influence of temperature [(see, for example, Ref. [17].


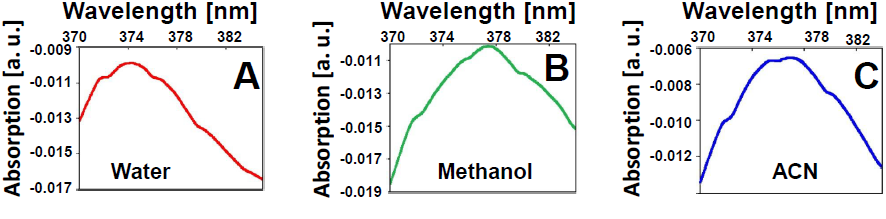


**Fig. S16.** Absorption spectra of water (A), methanol (B), and pure acetonitrile (C) taken at room temperature (23^0^ C) prior to laser treatment.

7. Supplemental data from mass spectrometry

7.1. Solvent-dependent modification of the test peptide

Mass spectroscopy confirmed that modifications of the peptide by excitation of peptide-solvent H-bonds in a room-temperature liquid were finely controlled by laser parameters and solvent.

*7.1.1 Solvent #1: water*

In water, the only laser-induced modifications were an increase in peptide mass by 16 Da (major signal) and a mass increase by 32 Da (very minor signal) associated with single and double oxidation, respectively (Fig. S17A). No significant influence of laser wavelength and peak intensity was observed except that the laser pulses converted 100% of the test peptide into the oxidized form at wavelength 386 nm and only 50% at 257 nm (Fig. S17B).

*7.1.2 Solvent #2: pure methanol (100%)*

In pure methanol, no significant modifications were observed, across a wide range of laser intensity (Fig. S18). The test peptide was subjected to laser pulses with pulse energy increasing from 59 to 230 μJ (Fig. S18B – G). A slight increase in a +16 Da modification was observed at higher laser intensities (Fig. S18F & G).

- - 1. *Solvent #3: methanol:water (98:2 % v/v)*

Laser treatment of the test peptide in methanol:water (98:2 % v/v) produced slightly elevated levels of +16 Da modification at higher intensities (Fig. S19).

- - 1. *Solvent #4: ACN:water (98:2 % v/v)*

In acetonitrile, laser irradiation caused the peptide to be almost completely converted (<1% unmodified remaining, by peak height) to modified forms (Fig. S20). The control, non-irradiated, peptide shows no modification (Fig. S20A). With low intensity laser irradiation (at 386 nm) four modified forms are visible i.e. +28 Da, +44 Da, +56 Da, and +72 Da (Fig. S20B). However, with high intensity laser irradiation, the most prominent form is +72 Da, with the other forms significantly diminished (Fig. S20C).

*7.1.5 Solvent #5: pure ACN (100%)*

In pure ACN, the major modification at all laser wavelengths and intensities was increase of peptide mass by 72 Da (Fig. S21). However, the relative abundance of minor modifications (+44 Da and +56 Da) did change with laser intensity and wavelength. Consistent with data for ACN:water (98:2 % v/v), the peptide in pure ACN was completely converted to modified forms with essentially no unmodified peptide remaining.


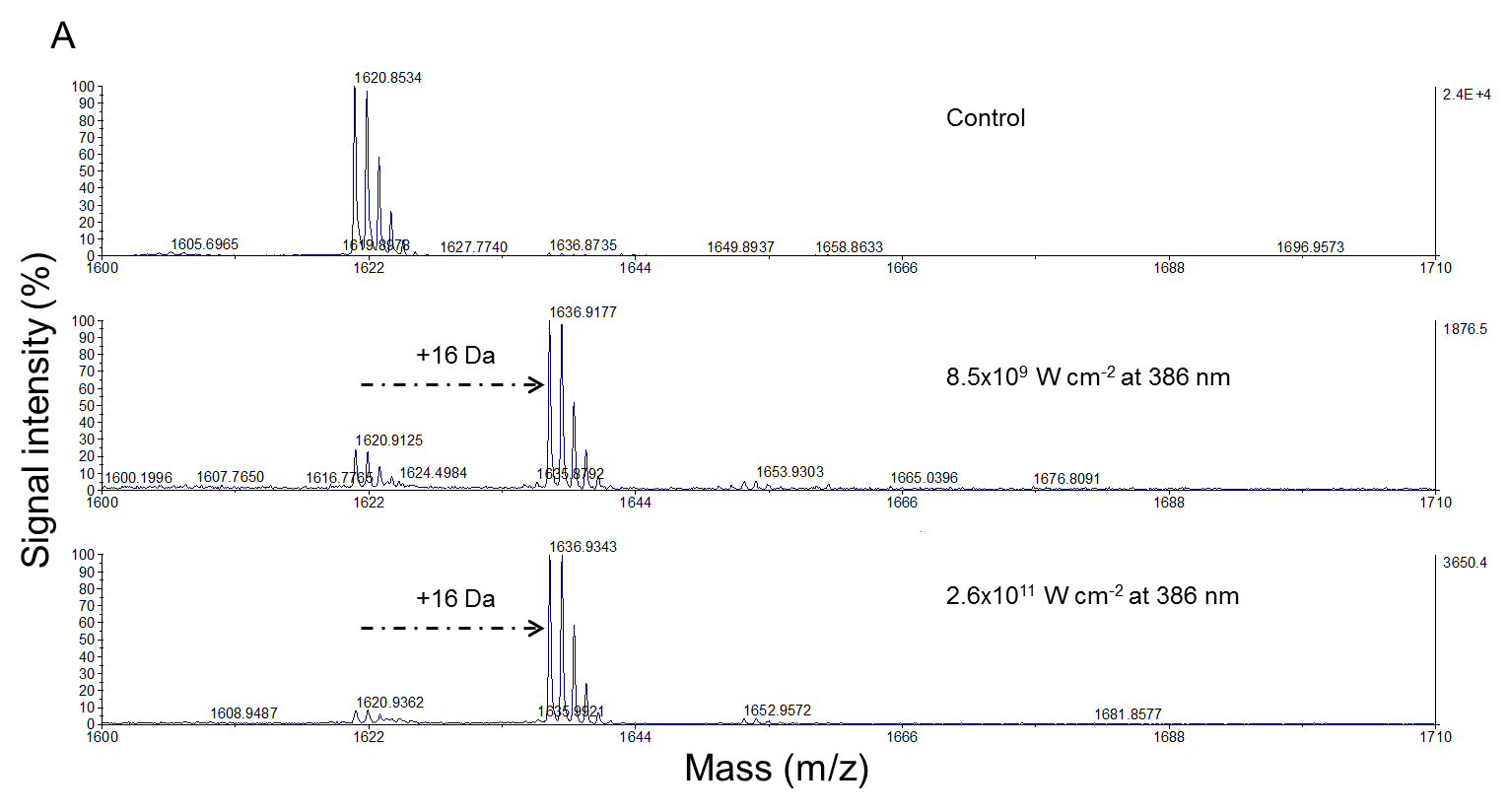

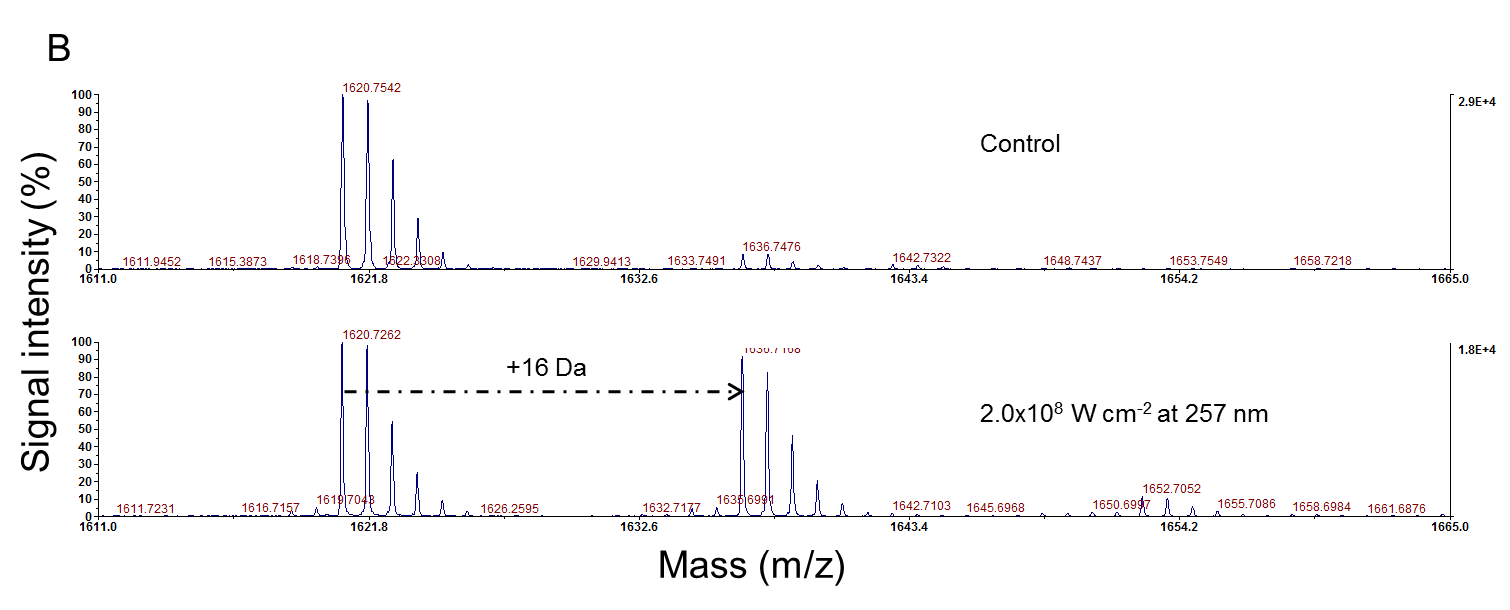


Fig. S17.

Mass spectra of the test peptide treated in water. The treatment was produced with laser pulses at 386 nm (A) (360,000 pulses; pulse energy 303.8 μJ; fluence 38.7 mJ/cm^2^) and 257 nm (B) (780,000 pulses; pulse energy 14.9 μJ: fluence 125.48 μJ/cm^2^). Modifications (Δmass) and laser irradiation parameters are indicated. Mass spectra of control (non-irradiated) peptides are shown in the top panels. Note: the mass range shown in part B is slightly different from part A.


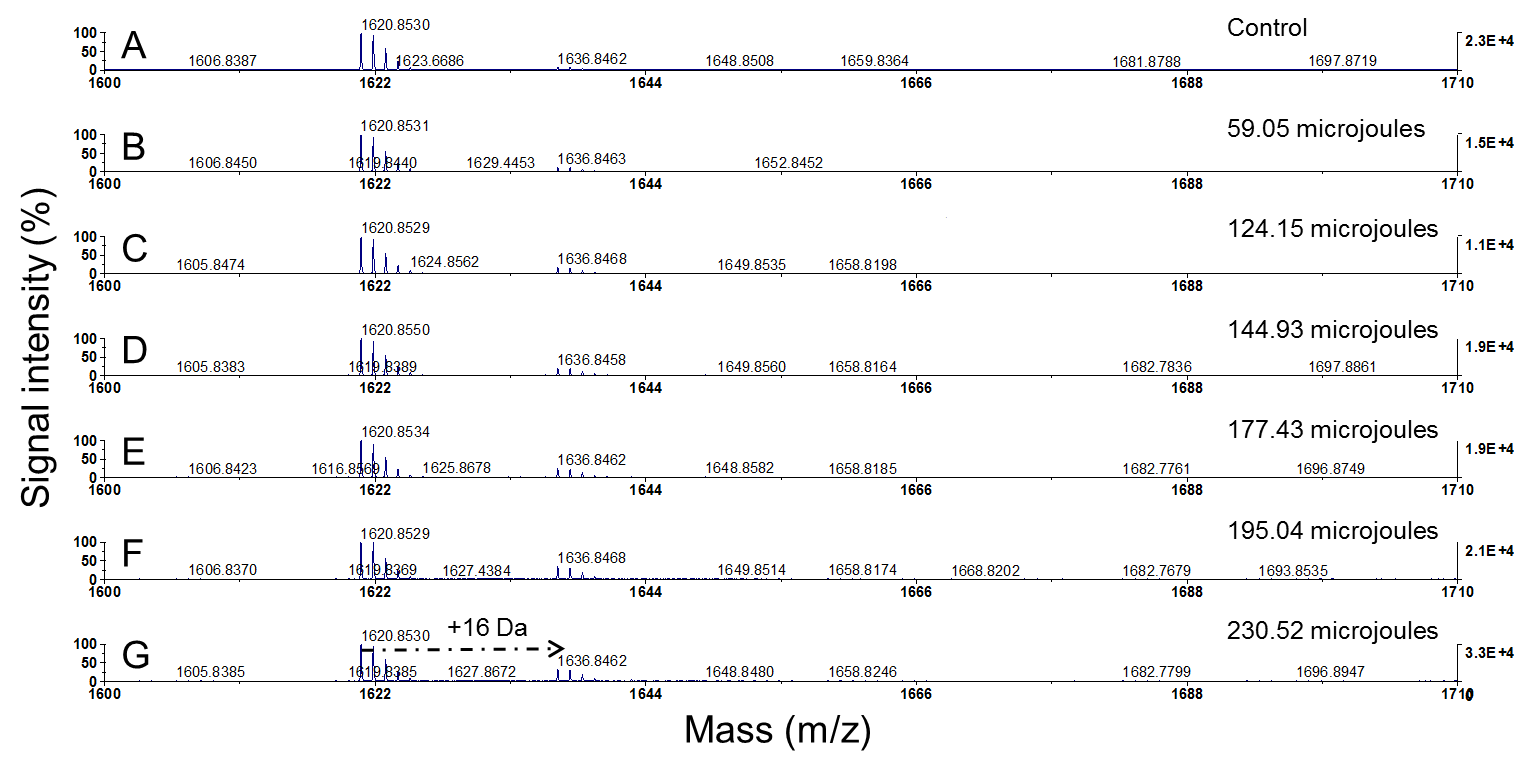


Fig. S18.

Mass spectra of the test peptide treated in pure methanol. The treatment was made by exposure to 600,000 laser pulses at several values of pulse energy (shown on the right side of each mass spectrum) at wavelength 386 nm (laser-spot diameter was 3.0 mm at front surface of cuvette). (A) Control, non-irradiated, peptide, (B) to (G) laser-irradiated peptide with increasing laser pulse energy as indicated. The only modification observed was a +16 Da mass increase (dashed arrow) that appeared to increase in relative abundance at higher intensities, compared to control.


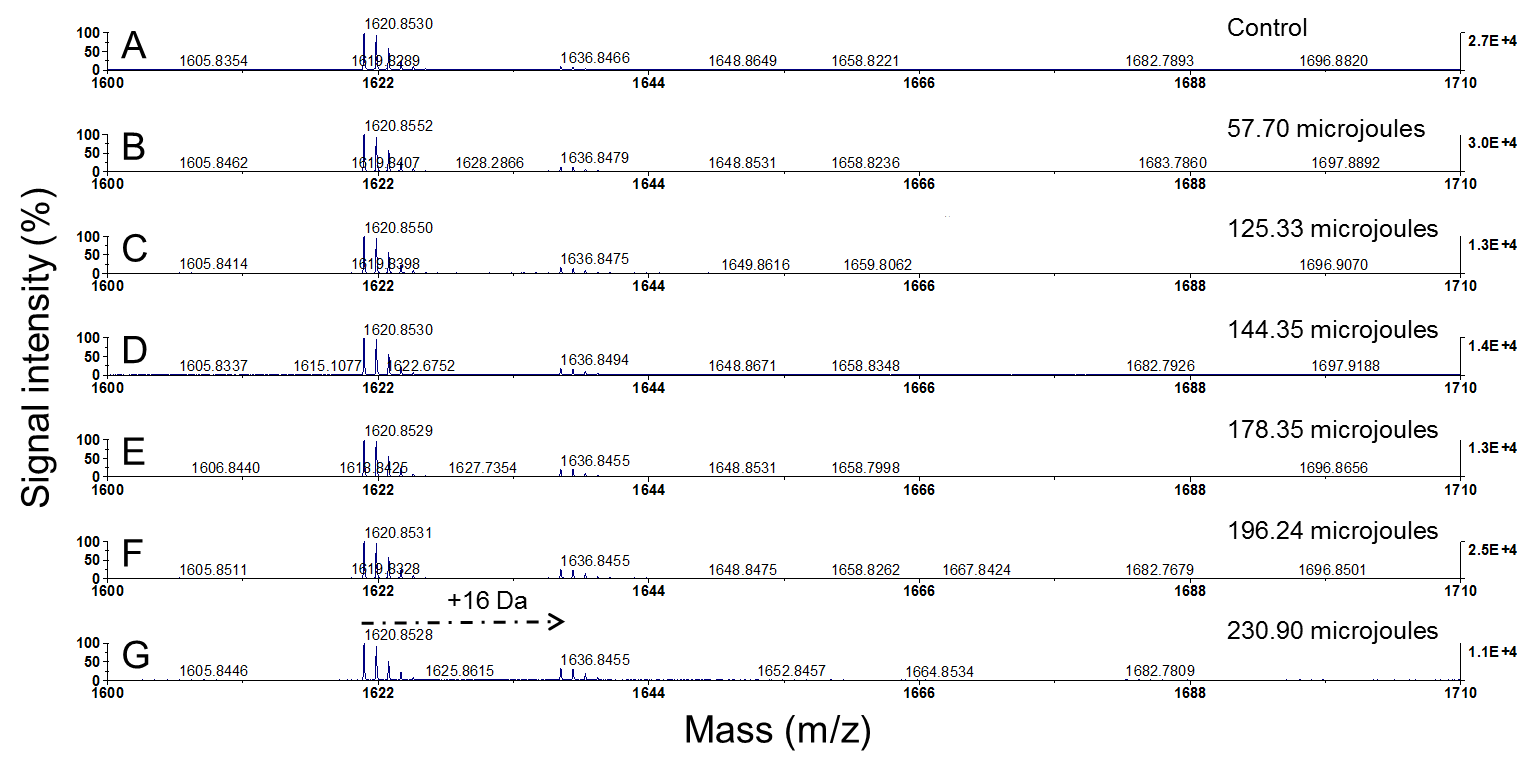


Fig. S19.

Mass spectra of the test peptide treated in a methanol:water mixture. The modification was produced by exposure to 600,000 laser pulses at several values of pulse energy (shown on the right side of each spectrum) at wavelength 386 nm, laser-spot diameter was 3.0 mm at front surface of cuvette. The test peptide was dissolved in methanol:water (98:2 % v/v). (A) Control, non-irradiated, peptide, (B) to (G) laser-irradiated peptide with increasing laser-pulse energy as indicated. The only modification observed was a +16 Da mass increase (dashed arrow) that appeared to increase in relative abundance at higher intensities.


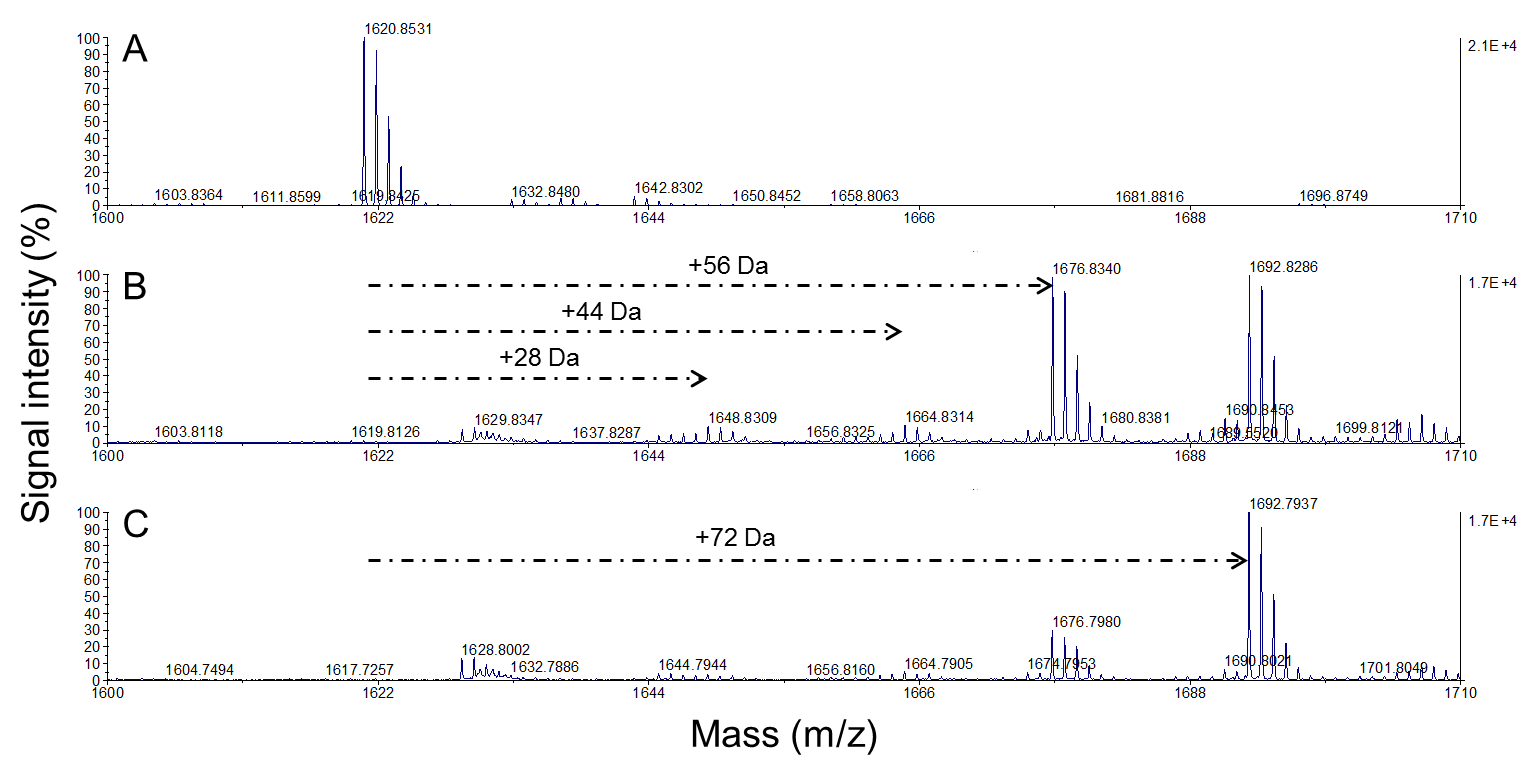


Fig. S20.

Mass spectra of the test peptide in ACN:water (98:2 % v/v). The sample was modified by 360,000 laser pulses at 386 nm with different intensity. (A) Control (non-irradiated) peptide, (B) peptide irradiated with pulses at pulse energy 260.3 μJ, fluence 1.64 mJ/cm^2^; laser intensity 10.9 GW/cm^2^, and (C) peptide irradiated at pulse energy 271.2 μJ, fluence 8.63 mJ/cm^2^, intensity 57.5 GW/cm^2^. Modifications, including change in mass, are indicated with dashed arrows.


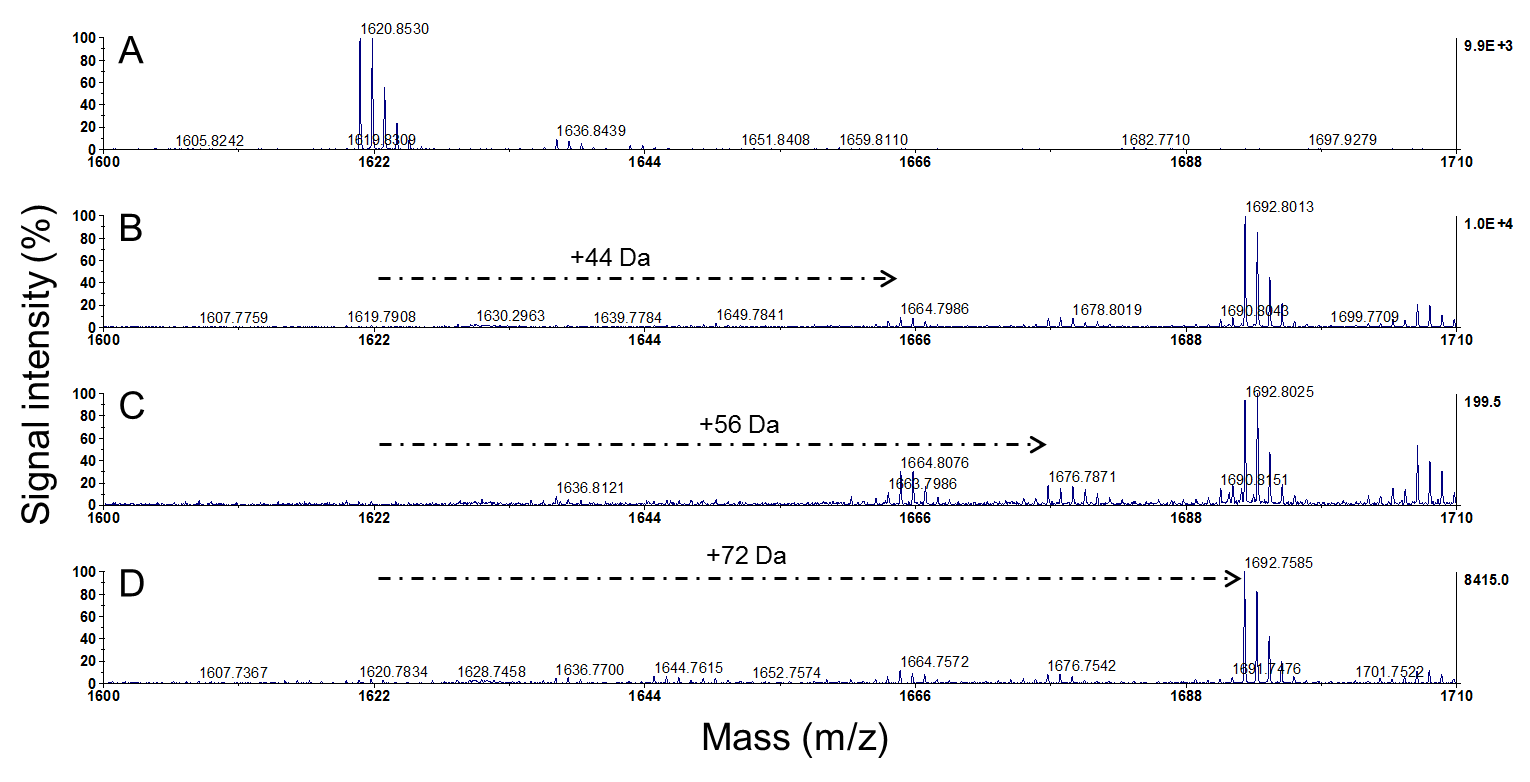


Fig. S21

Mass spectra of the test peptide in pure ACN treated by laser pulses at 386 nm and 257 nm. (A) Control (non-irradiated) peptide, (B) 600,000 pulses at pulse energy 313.96 μJ, fluence 4.44 mJ/cm^2^, intensity 29.6 GW/cm^2^ at wavelength 386 nm, (C) 600,000 pulses at pulse energy 359.7 μJ, fluence 20.36 mJ/cm^2^, intensity 135.7 GW/cm^2^ at wavelength 386 nm, and (D) 1,200,000 pulses at pulse energy 15.05 μJ, fluence 0.128 mJ/cm^2^, intensity 0.852 GW/cm^2^ at wavelength 257 nm. Modifications, including changes in mass, are indicated with dashed arrows.

7.2. Peptide modification is controlled by varying water content

To study the influence of solvent composition on peptide modification, laser wavelength and intensity was kept constant while the amount of water present was varied. Water content was varied from 1% to 90% (v/v) in ACN (Fig. S22). Laser-induced modification of the peptide in 99:1 ACN:water resulted in a prominent +72 Da modified peptide and about 10% of that peak height is the +56 Da form (Fig. S22B). This result was essentially identical to “standard conditions” of 98:2 (% v/v) ACN:water. No obvious differences in peak intensity or modified forms were observed when the water content was increased to 10% relative to ACN (Fig. S22C). However, when the water content was increased to 50%, a +44 Da form became more prominent with a concomitant reduction in the relative amount of the +72 Da form (Fig. S22D). Finally, when the water content was increased to 90% relative to ACN, the +56 Da and +72 Da forms were greatly reduced in intensity and a prominent +44 Da form was observed. A minor amount of a +16 Da form was also present (Fig. S22E). These data show precise control of peptide modification is possible by adjusting the water content, while keeping the laser intensity and wavelength constant.


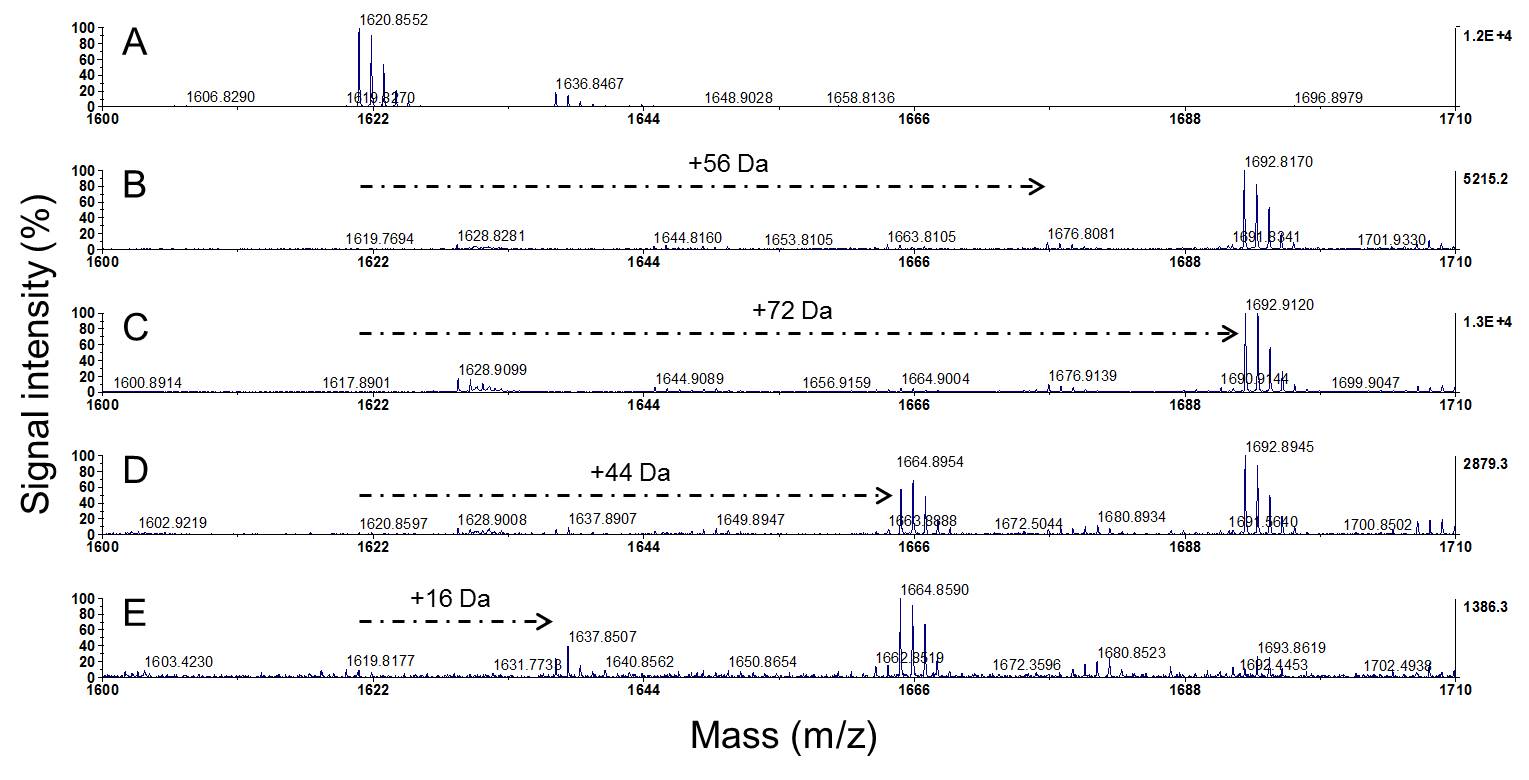


Fig. S22.

Modification of the test peptide relative to water content. Laser treatment was done by 360,000 pulses at average pulse energy 307 μJ, laser fluence 0.787 mJ/cm^2^ and intensity 52.5 GW/cm^2^ (at 386 nm). (A) Control (non-irradiated) peptide, (B) laser-irradiated peptide in 99:1 (% v/v) ACN:water, (C) laser-irradiated peptide in 90:10 (% v/v) ACN:water, (D) laser-irradiated peptide in 50:50 (% v/v) ACN:water, and (E) laser-irradiated peptide in 10:90 (% v/v) ACN:water. Modifications, including change in mass, are indicated with dashed arrows.

7.3. Observed modifications are formylation on primary amines and methionine oxidation

Tandem mass spectrometry, both MALDI TOF-TOF MS/MS and Orbitrap HCD MS/MS, was used to fragment the modified peptides. *De novo* sequencing was then used to map sites and types of modification. High-resolution, high-mass-accuracy Orbitrap FTMS was used to calculate accurate mass differences between unmodified and modified peaks. The peptide sample described in this sub-section was treated with 360,000 laser pulses at pulse energy 304.3 μJ, fluence 6.20 mJ/cm^2^, and intensity 41.3 GW/cm^2^ at wavelength 386 nm.

7.3.1 Mapping sites of modification

To define the sites of the modifications, MALDI TOF-TOF MS/MS and LTQ Orbitrap HCD MS/MS of the test peptide (GPTLKRTASTPFMNT_amide_) were utilized. An initial attempt to define the sites of modifications employed Orbitrap HCD (high energy collision induced dissociation) of the 1676 Da peptides (+56 Da). Robust fragmentation was observed and *de novo* sequencing revealed a b-ion comprising the first 5 residues (GPTLK) of the test peptide, whose mass was increased by 56 Da along with a series of 4 additional b-ions all increased in mass by 56 Da relative to the theoretical fragment masses (Fig. S23). Although sequence coverage in the MS/MS spectrum was poor, this did confirm that the first 5 residues must contain one or more modifications (b-ion data) and that the last 9 residues were unmodified (y-ion data).

To improve sequence coverage of the N-terminal region (and confirm sites of modification), a trypsin digestion of the laser-irradiated peptide preparation was conducted (Fig. S24). Trypsin digestion produced a prominent N-terminal portion (GPTLKR_+56 Da_) and a lower intensity C-terminal portion (TASTPFMNT_amide_) of the modified peptide (Fig. S24A). Complete sequence coverage for the N-terminal portion of the peptide was obtained by MALDI TOF-TOF MS/MS (Fig. S24B). It is clear from these data that there is a 28 Da modification on the first residue (Gly) as well as on the Lys residue. Both of these amino acid residues are primary amines (N-terminus) and Lys. This confirmed that the laser-induced modification of peptides adds 28 Da to primary amines. No other residues in this portion of the peptide were modified. However, TOF-TOF and Orbitrap MS/MS of the +72 Da precursor (1692.8 Da) revealed that the Met residue was converted to Met-sulfoxide, which is a relatively common modification. It does appear that laser irradiation, at 257 and 386 nm and above a certain threshold of intensity, causes Met-oxidation in all solvents tested (cf. Figs. S17A&B, S18, S19, and Fig. 2). Having established that primary amines were modified by +28 Da, high-resolution, high-mass-accuracy Orbitrap FTMS was repeated with internal calibration to accurately calculate the mass differences observed, and therefore be able to predicttheir atomic composition (see section 7.3.2).

7.3.2 Determination of the atomic composition of laser-induced modifications

To determine the most likely chemical composition of the observed modifications, high-resolution, high-mass-accuracy Orbitrap FTMS spectra were acquired. MS spectra were internally calibrated using the unmodified peptide (spiked in to the laser-irradiated sample) as a reference or “lock mass”. The MS spectrum shows the unmodified peptide as a doubly charged (z=2) molecular ion of 810.928 m/z (Fig. S25). The +44, +56, and +72 Da modified forms were also observed. The mass differences between peaks were calculated to the 5^th^ decimal place and then the elemental composition was determined using the Elemental Composition Calculator v1.0 (Table S4). The chemical composition of the mass shifts observed is summarized in Table S5.


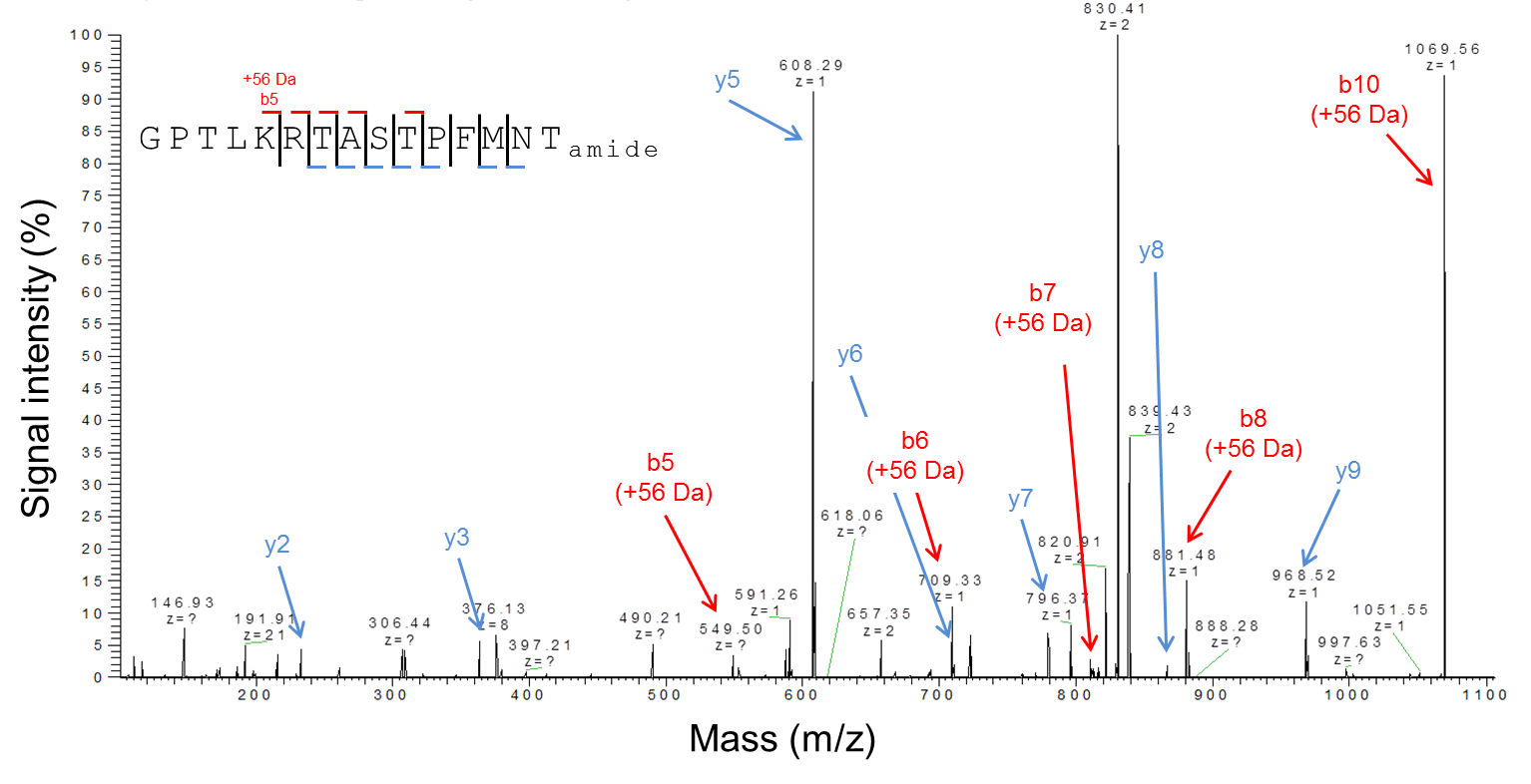


Fig. S23

Orbitrap HCD MS/MS spectrum of the +56 Da modified test peptide. The molecular ion (precursor) corresponding to the +56 Da modified peptide (838.94 m/z, 1676.8 Da) was isolated and fragmented using high energy collision-induced dissociation. N-terminal (b-ion) and C-terminal (y-ion) fragments are indicated in red and blue type, respectively. Sequence coverage for the peptide is shown on the top left.


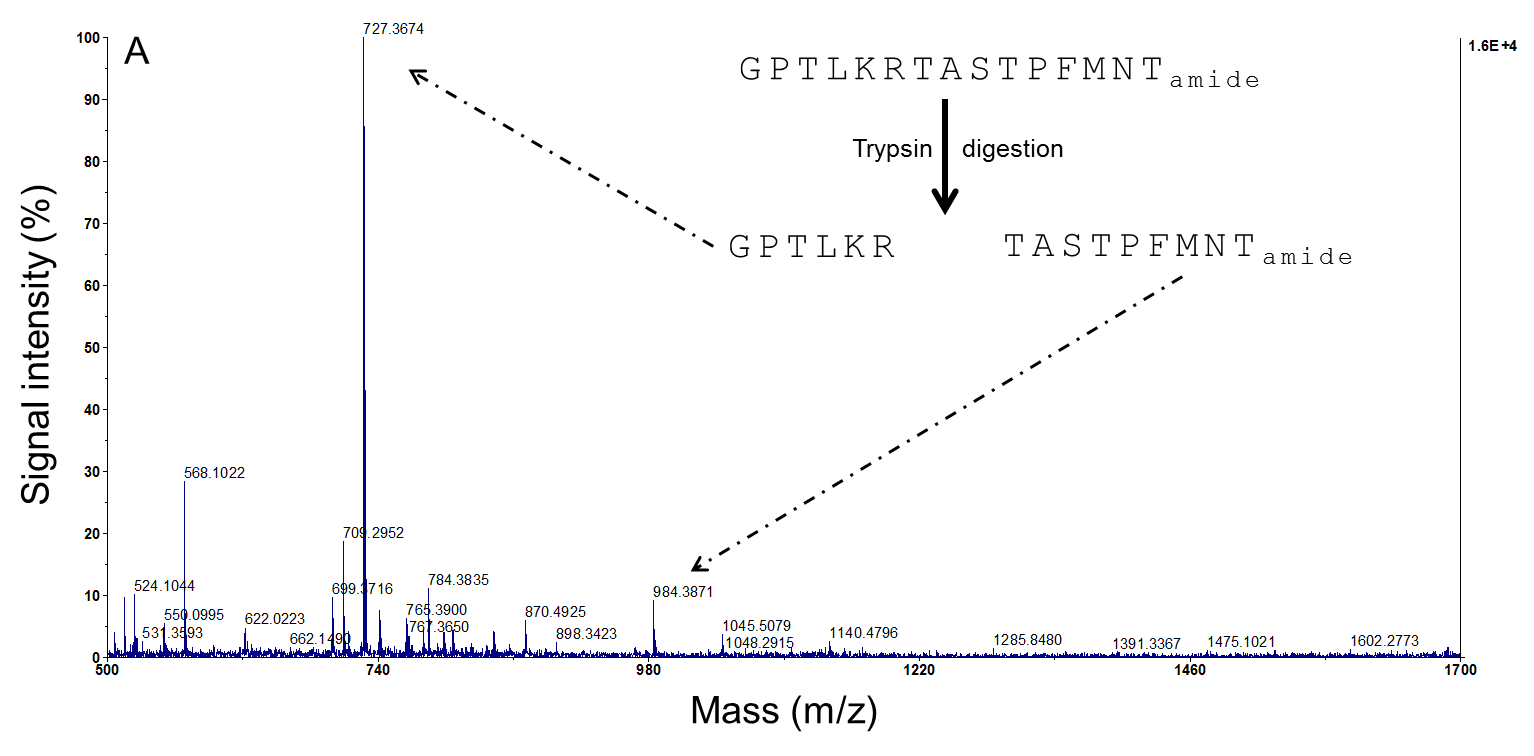


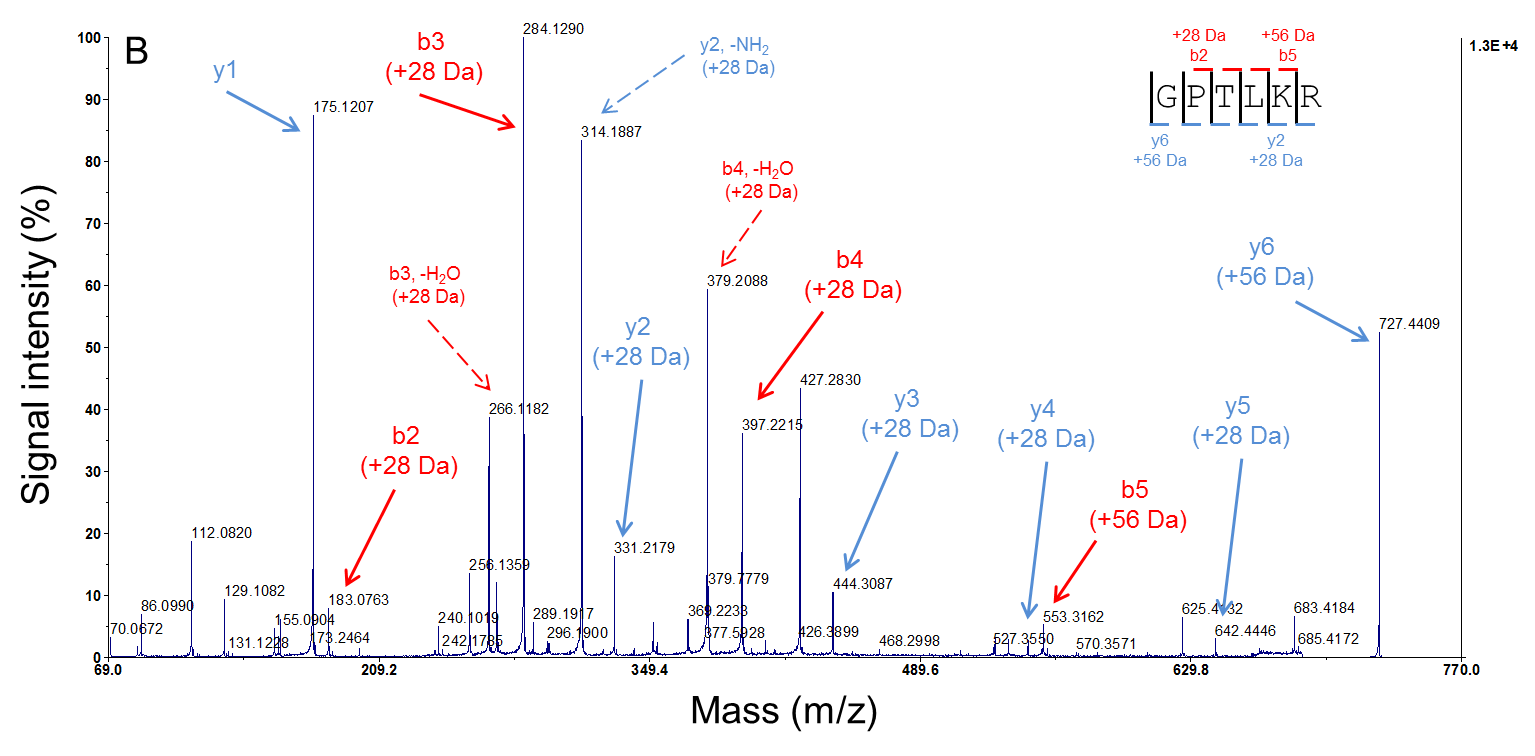


Fig. S24

MALDI TOF MS and TOF-TOF MS/MS spectra of trypsin-digested +56 Da modified test peptide. (A) Trypsin digestion of the modified peptide resulted in two fragments, an N-terminal portion of 727 Da (corresponding to GPTLKR+56Da) and a C-terminal portion of 984 Da (corresponding to TASTPFMNTamide). (B) The 727 Da molecular ion (precursor) was isolated and fragmented. N-terminal (b-ion) and C-terminal (y-ion) fragments are indicated in red and blue type, respectively. Sequence coverage for the peptide is shown on the top right. Modifications of 28 Da occur on both the N-terminal Gly and the Lys residue (primary amines).


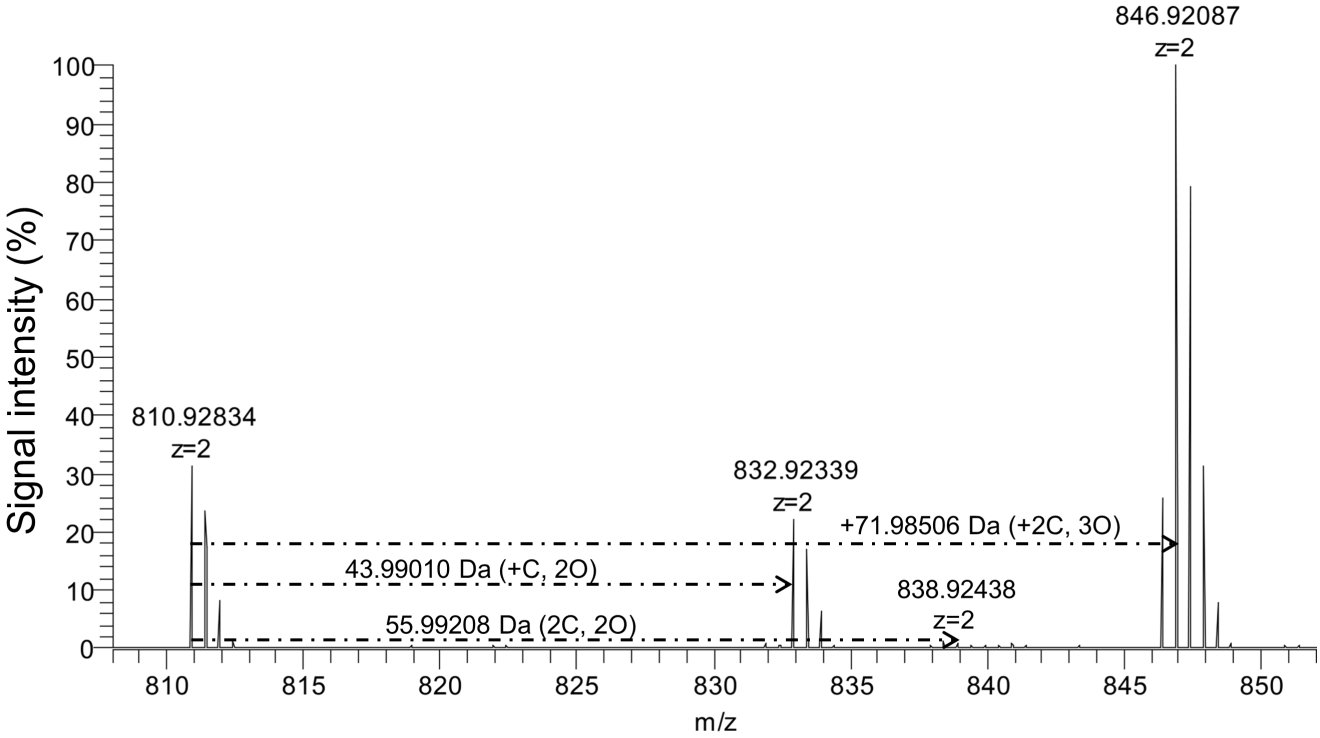


Fig. S25

High resolution ESI-Orbitrap FTMS spectrum of the test peptide in ACN:water (98:2 % v/v). The modification was produced with 360,000 laser pulses at wavelength 386 nm. The signal at 810.92834 m/z comes from the non-modified control peptide, which was spiked in to give a reference peak. See Table S6 for more information about the changes in mass.

Table S6.

Possible elemental composition of mass differences. The elemental composition was calculated from the data in Fig. S25 using Elemental Composition Calculator v1.0 (<http://mods.rna.albany.edu/masspec/Elcomp>, Jeff Rozenski) using the following masses: C: 12.000, H: 1.00782, N: 14.00307, O: 15.99492, S: 31.97200. The shown elemental compositions were found to be within 250 parts per million (ppm) mass difference. In all cases, the predicted elemental composition had significantly lower mass derivation than the others (red type).

| Observed mass differences | C | H | N | O | S | ppm |
| --- | --- | --- | --- | --- | --- | --- |
| +43.99010 | - | - | 2 | 1 | - | -249.1 |
|  | **1** | **-** | **-** | **2** | **-** | **5.9** |
| +55.99208 | **2** | **-** | **-** | **2** | **-** | **40.0** |
|  | 1 | - | 2 | 1 | - | -160.3 |
| +71.98506 | 1 | - | 2 | 2 | - | -151.6 |
|  | **2** | **-** | **-** | **3** | **-** | **4.1** |
|  | 2 | 2 | 1 | - | 1 | -78.4 |
|  | 1 | - | 2 | - | 1 | 96.1 |

Table S7.

Atomic groups that are attached to the test peptide by the laser treatment. Corresponding mass changes for the laser-induced modification of the peptide in water-acetonitrile solvent (Fig. S25) are shown in the third column.

| Attached atomic group(s) | Type of laser-induced modification | Mass change, Da |
| --- | --- | --- |
| CO | Single formylation; no oxidation | + 28 |
| CO + O | Single formylation; single oxidation | + 44 |
| CO + 2 O | Single formylation; double oxidation | + 60 |
| 2 CO | Double formylation; no oxidation | + 56 |
| 2 CO + O | Double formylation; single oxidation | + 72 |
| 2 CO + 2 O | Double formylation; double oxidation | + 88 |
| O | Single oxidation | + 16 |
| 2 O | Double oxidation | + 32 |

7.4. Solvent contributions to formylation and oxidation: sources of carbon and oxygen

Isotope-labelled water H_2_^18^O and acetonitrile ^13^CH_3_CN were utilized as solvents to clarify the possible mechanisms of the laser-induced modifications of the test peptide. In methanol:H_2_^18^O (98:2 % v/v) and pure water, no evident laser modification except slightly increased oxidation was observed (Fig. S26). No evidence for ^18^O incorporation was observed in any mass spectra obtained for laser-modified peptide in pure H_2_^18^O (Fig. S26B) or in methanol:H_2_^18^O (Fig. S26C).

In ^13^CH_3_CN:H_2_O^18^ (98:2 % v/v), laser-induced peptide modifications were observed (Fig. S27). Instead of the “standard” +28/+56 Da forms, a prominent +29 Da form was observed along with a lesser amount of a +58 Da form (Fig. S27B). The MALDI-TOF mass spectrum for the laser-irradiated peptide was quite complex, with multiple overlapping isotope envelopes from 1647 Da through 1703 Da observed. In an attempt to clarify the observed modifications, high-resolution, high-mass-accuracy Orbitrap FTMS spectra were acquired (Fig. S28). The data show a clear doubly-charged peak at +14.4989 m/z corresponding to a Δmass of 28.998 Da, i.e. ^13^CO. No O^18^ incorporation was observed.

To further elucidate the contribution of water as a source of oxygen in modifying the peptide, unlabeled ACN mixed with H_2_^18^O was used for as a solvent (Fig. S29). No incorporation of ^18^O atoms into modifications was observed. The usual +28 Da, +56 Da, and +72 Da modifications were observed suggesting that dissolved oxygen modifies the peptides during laser irradiation.


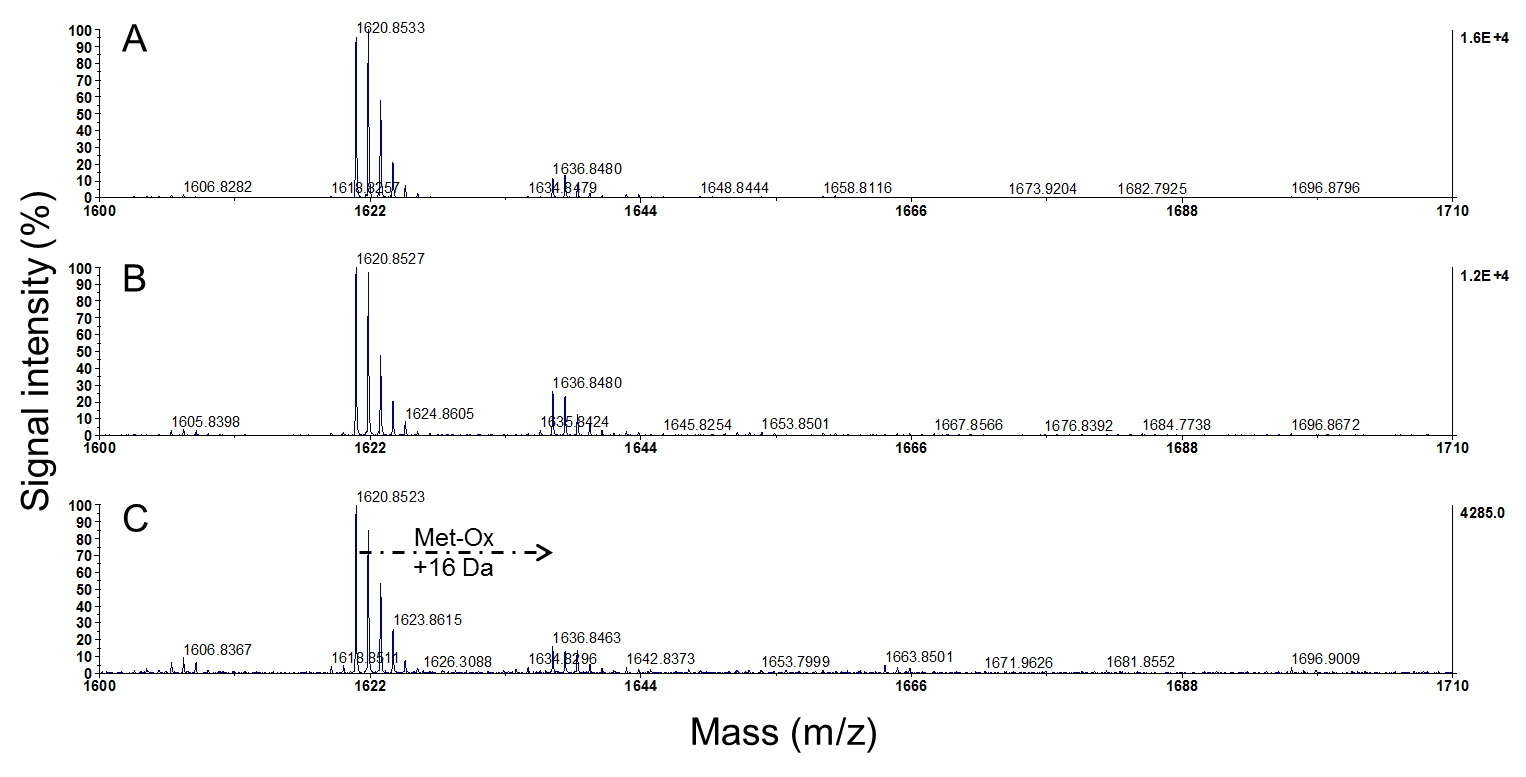


Fig. S26

Mass spectra of the test peptide treated in isotope-labeled methanol:water. (A) Control (non-irradiated) peptide mass spectrum, (B) peptide treated with 240,000 laser pulses at wavelength 386 nm and pulse energy 301.53 μJ, fluence 1.07 mJ/cm^2^, intensity 7.11 GW/cm^2^ in methanol:H_2_^18^O (98:2 % v/v), and (C) peptide treated with 240,000 pulses at wavelength 386 nm and pulse energy 299.89 μJ, fluence 1.06 mJ/cm^2^, intensity 7.07 GW/cm^2^ in pure H_2_^18^O. Modifications, including change in mass, are indicated with dashed arrows.


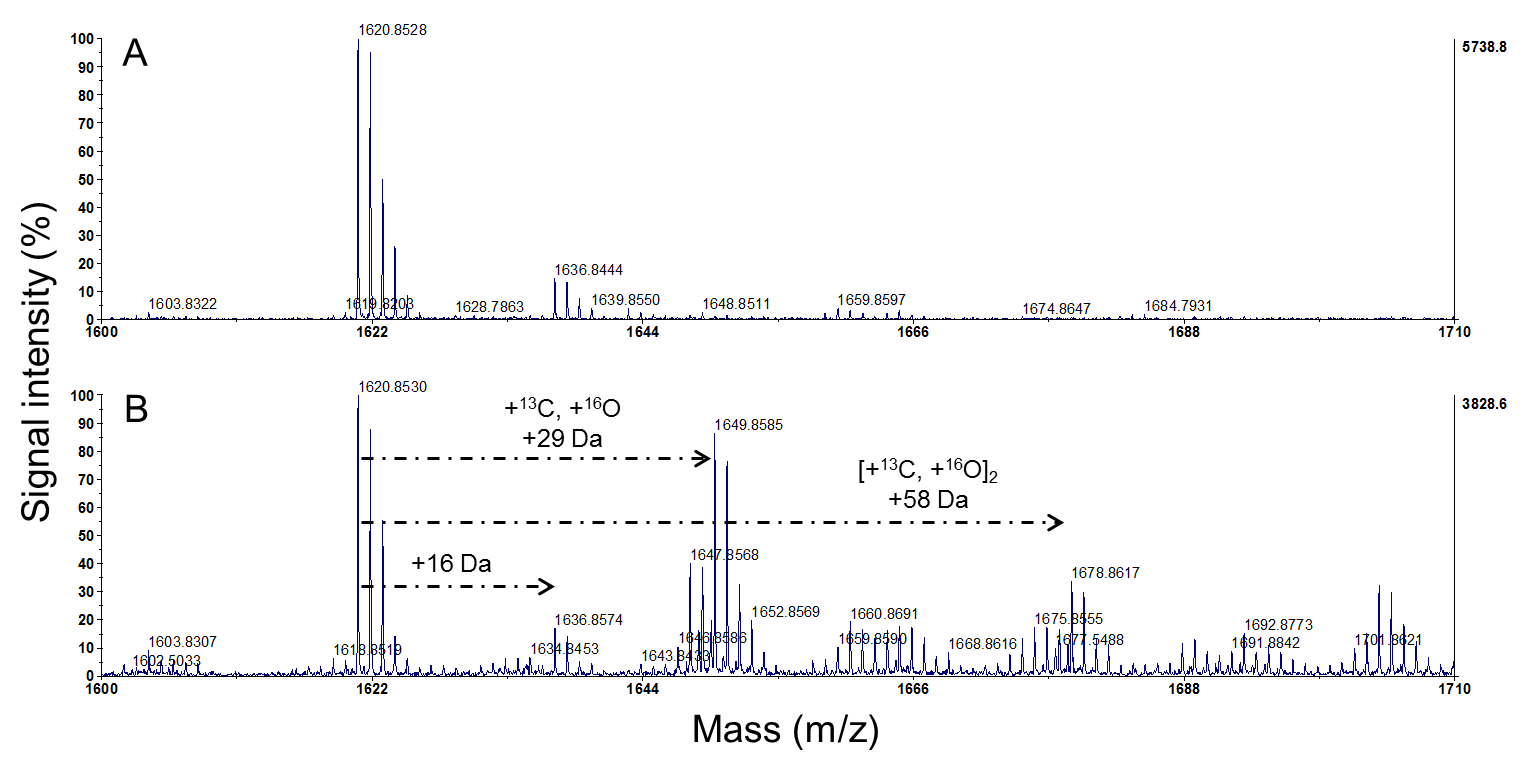


Fig. S27

Mass spectra of the test peptide treated in isotope-labeled ACN:water. The treatment was done by 240,000 laser pulses at wavelength 386 nm and pulse energy 299.28 μJ, fluence 1.06 mJ/cm^2^, intensity 7.06 GW/cm^2^ in a mixture of isotope-labelled acetonitrile ^13^CH_3_CN (98 % v/v) and isotope-labeled water H_2_^18^O (2% v/v). (A) Control (non-irradiated) peptide in ^13^CH_3_CN:H_2_^18^O (98:2 % v/v), (B) laser-irradiated peptide in ^13^CH_3_CN:H_2_^18^O. Modifications, including change in mass, are indicated with dashed arrows.


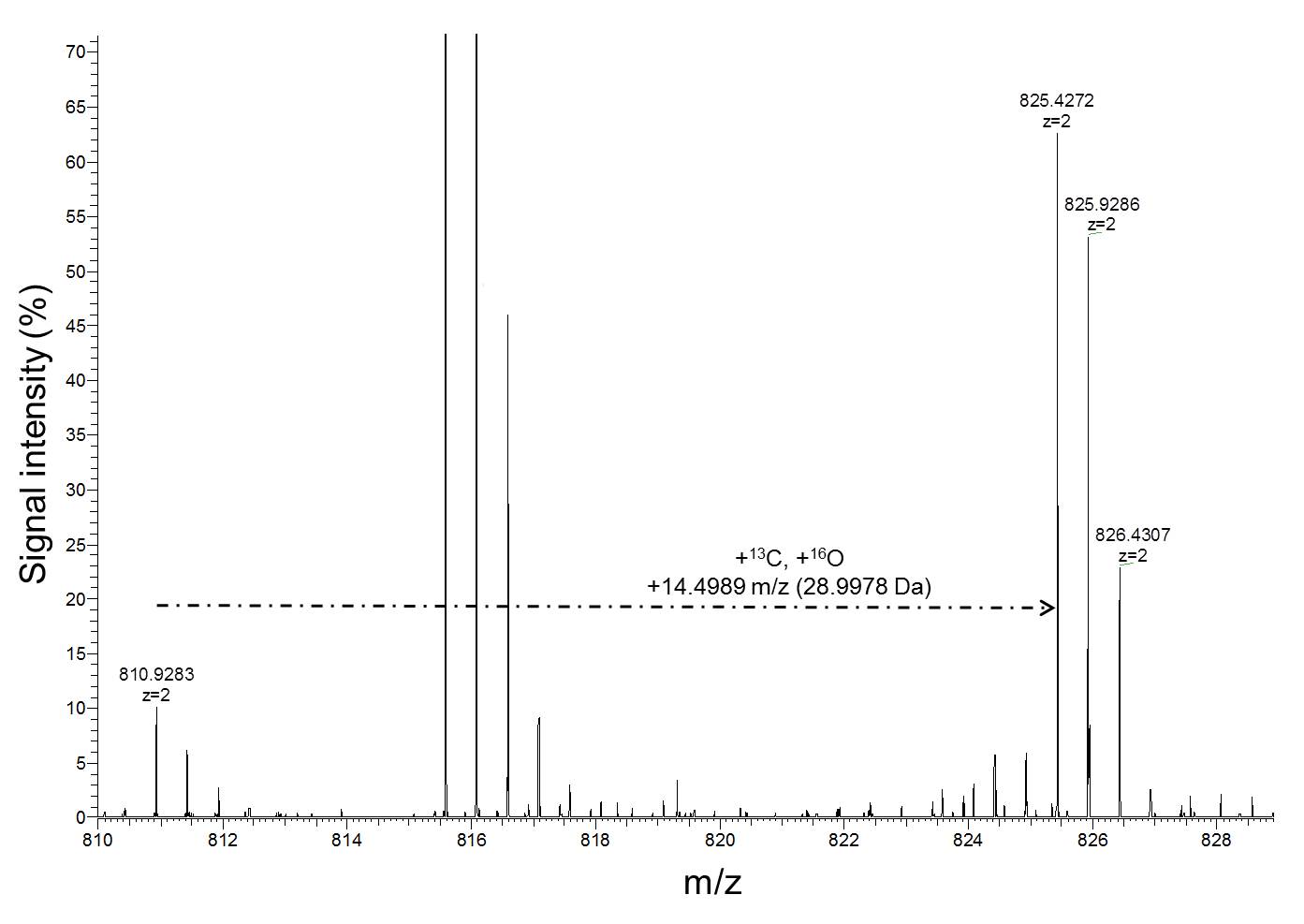


Fig. S28

High-resolution mass spectrum of the test peptide treated in isotope-labeled ACN:water. The exposure to 240,000 laser pulses was done in ACN:water, ^13^CH_3_CN:H_2_^18^O, (98:2 % v/v). FTMS spectrum for laser-irradiated peptide sample, m/z range 800-828, showing unlabeled and +29 Da peaks (dashed arrow). Laser-treatment parameters are as in Fig. S27.


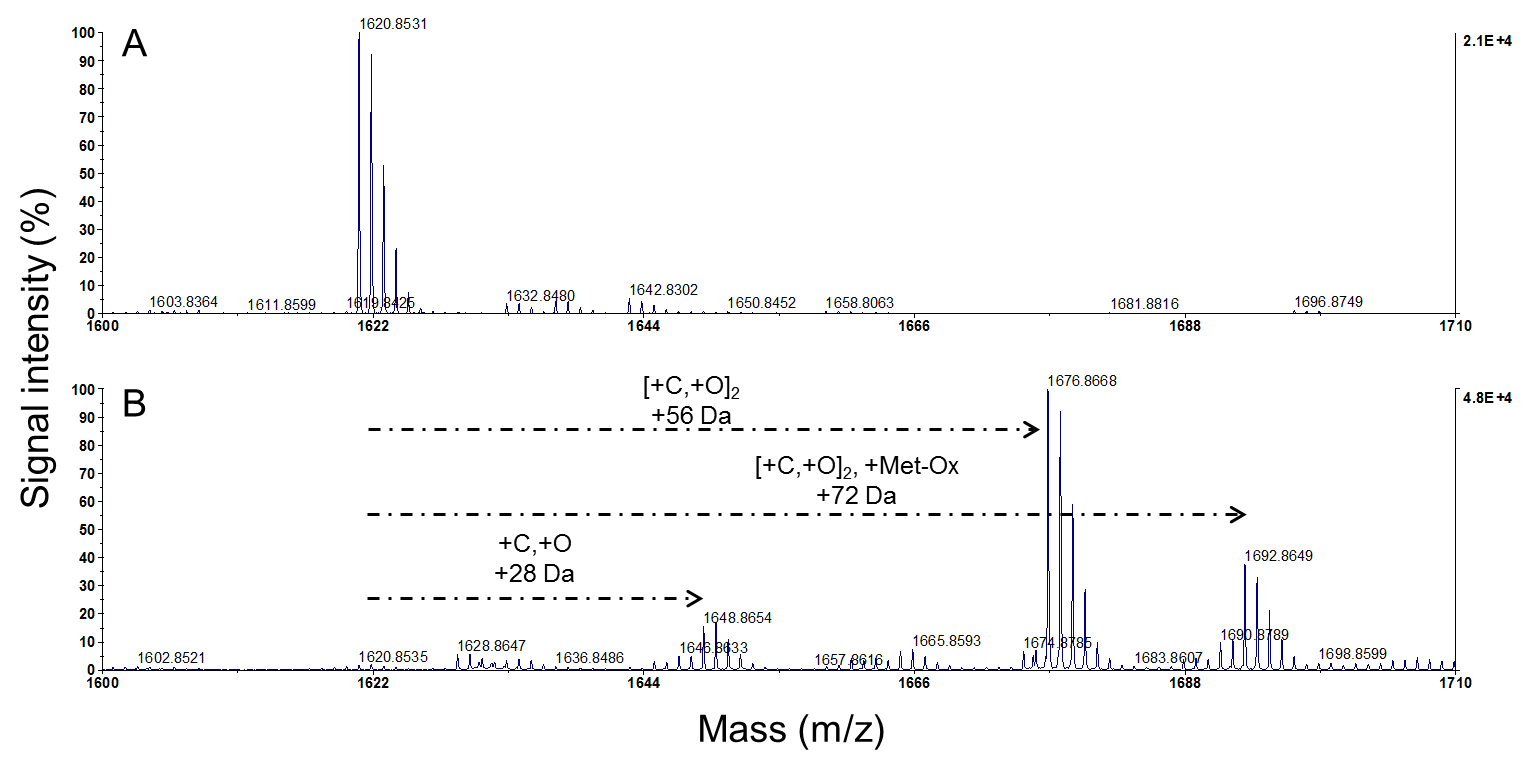


Fig. S29

Mass spectrum of the test peptide modified in a mixture of unlabeled ACN:H_2_^18^O (98:2 % v/v). (A) Control (non-irradiated) peptide in ACN:H_2_^18^O (98:2 % v/v), (B) laser-irradiated peptide in ACN:H_2_^18^O (360,000 pulses; pulse energy 128.45 μJ, fluence 387.11 μJ/cm^2^, intensity 2.59 GW/cm^2^; wavelength 386 nm). Modifications, including change in mass, are indicated with dashed arrows.

7.5. Influence of air on formylation can be suppressed by bubbling argon through solution

To further check if dissolved oxygen and carbon dioxide contributed to peptide modification, an argon sparging experiment was conducted. The test peptide was re-suspended in aqueous solvent (ACN:water, 10:90 % v/v) and argon gas was bubbled through the solution for 15 minutes prior to laser irradiation at 386 nm (Fig. S30). Under control conditions (i.e. no sparging), a prominent +44 Da peak (single formylation + Met-oxidation) was observed (Fig. S30A). Following argon sparging, no formylation was observed, and only a portion of the peptide was Met-oxidized, with a significant amount of unmodified peptide remaining (Fig. S30B). These data show that modification of ambient atmosphere significantly affects products of laser treatment and confirms the influence of dissolved gases on the peptide modification.


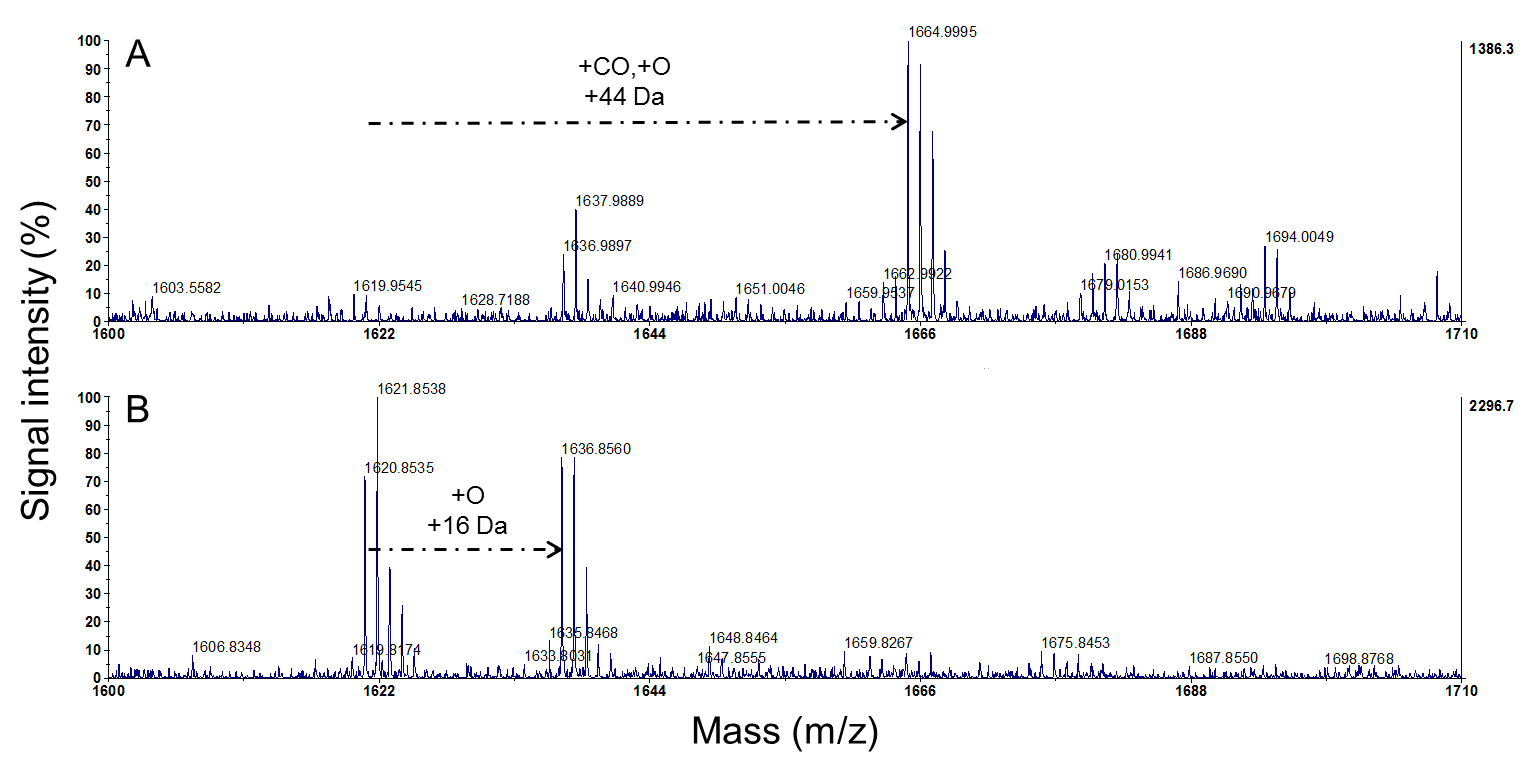


Fig. S30

Modification of the test peptide following argon sparging. The test peptide was resuspended in aqueous solvent (10:90 ACN:water, % v/v) and subjected to laser treatment at 386 nm (360,000 pulses; pulse energy 307.06 μJ, fluence 797.90 μJ/cm^2^, intensity 5.32 GW/cm^2^ at wavelength 386 nm). (A) Laser irradiated peptide without sparging argon, and (B) argon sparging followed by laser irradiation. Modifications are indicated with dashed arrows.

7.6. Free lysine (Lys) is not modified upon laser irradiation

To determine if free Lys could be modified by laser irradiation, a series of experiments was conducted in which solvent and laser conditions were altered to mimic those conditions shown to facilitate peptide modification (Fig. S31). However, no modification of free Lys was observed, irrespective of solvent, laser wavelength, or laser intensity (Fig. S31 A-F). This suggests that the local solvent environment of residues in a polypeptide chain is critical for the laser modification.


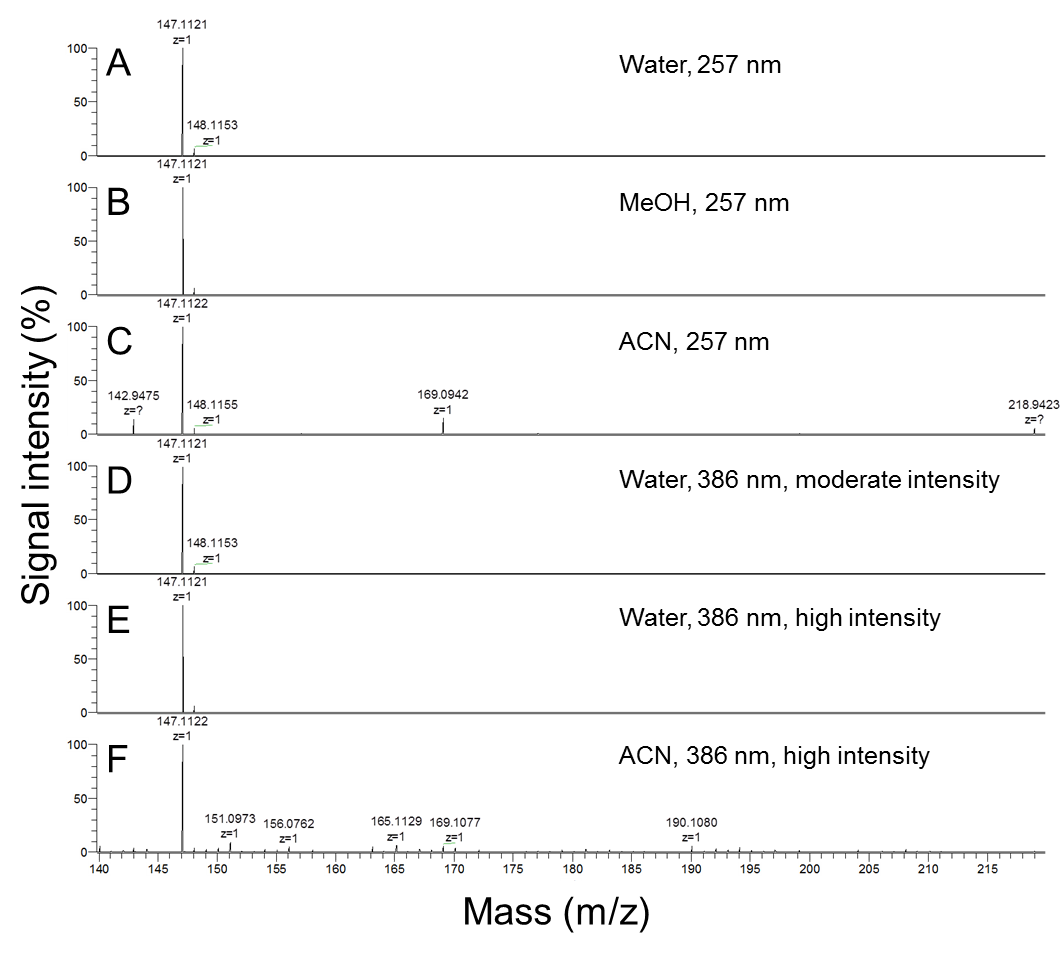


Fig. S31

Mass spectra of laser-irradiated free Lys. Solvent and laser conditions were altered as indicated in the figure. (A) water, wavelength 257 nm, 540,000 pulses, pulse energy 12.87 μJ, fluence 182.1 μJ/cm^2^, intensity 1.21 GW/cm^2^; (B) 100% methanol, wavelength 257 nm, 600,000 pulses, pulse energy 11.93 μJ, fluence 168.8 μJ/cm^2^, intensity 1.13 GW/cm^2^; (C) 100% ACN, wavelength 257 nm, 180,000 pulses, pulse energy 11.92 μJ, fluence 168.6 μJ/cm^2^, intensity 1.12 GW/cm^2^; (D) water, wavelength 386 nm, 540,000 pulses, pulse energy 307.7 μJ, fluence 4.35 mJ/cm^2^, intensity 29.02 GW/cm^2^; (E) water, wavelength 386 nm, 540,000 pulses, pulse energy 306.8 μJ, fluence 17.36 μJ/cm^2^, intensity 115.8 GW/cm^2^; and (F) 100% ACN, wavelength 386 nm, 360,000 pulses, pulse energy 307.7 μJ, fluence 17.41 mJ/cm^2^, intensity 116.1 GW/cm^2^;. No modification of free Lys was observed.

7.7. Laser-induced formylation of human serum albumin (HSA) peptides

To confirm that laser-induced modification was not an artifact of our test peptide, experiments were done on a HSA seven-peptide standard. HSA peptides were re-suspended in ACN:water (98:2 % v/v) and irradiated at a wavelength of 386 nm. Intensity was kept close to the lowest level proven to modify the test peptide. Following laser irradiation, peptides were diluted 1:1 with acidified ACN (ACN:formic acid:water, 70:29:1 % v/v) and analyzed by Orbitrap FTMS.

The results demonstrated a universal character of the laser modifications produced on the test peptide. In particular, many HSA peptides showed an increase in their mass by 28 Da (Fig. S32). All peptides were formylated by laser irradiation (Fig S32 A-G). The only peptide containing a Met residue was present exclusively as the Met-oxidized or Met-oxidized Lys-formylated forms i.e. no un-oxidized peptide was observed (Fig. S32B). The only peptide without a Lys residue (YLYEIAR) was formylated, presumably at the terminal primary amine, though at a much reduced conversion rate (Fig S32D). The conversion rate for most peptides was 40-50% (by peak height); however, the conversion rate of the HPYFYAPELLFFAK peptide was about 90%, similar to the test peptide (Fig. S32F). Additionally, this was the only peptide to exhibit detectable +56 Da double formylation (Fig. S32F, inset).


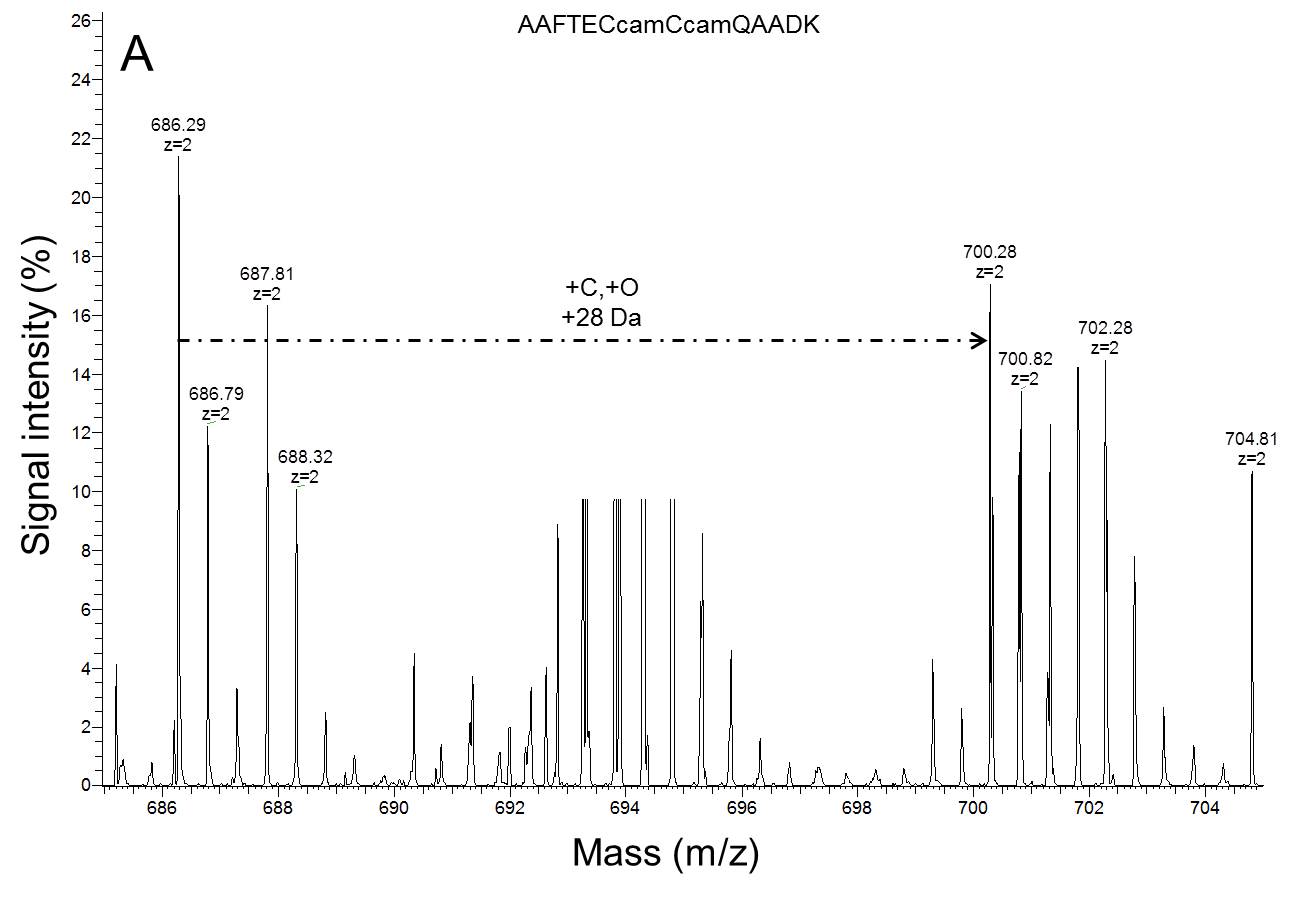


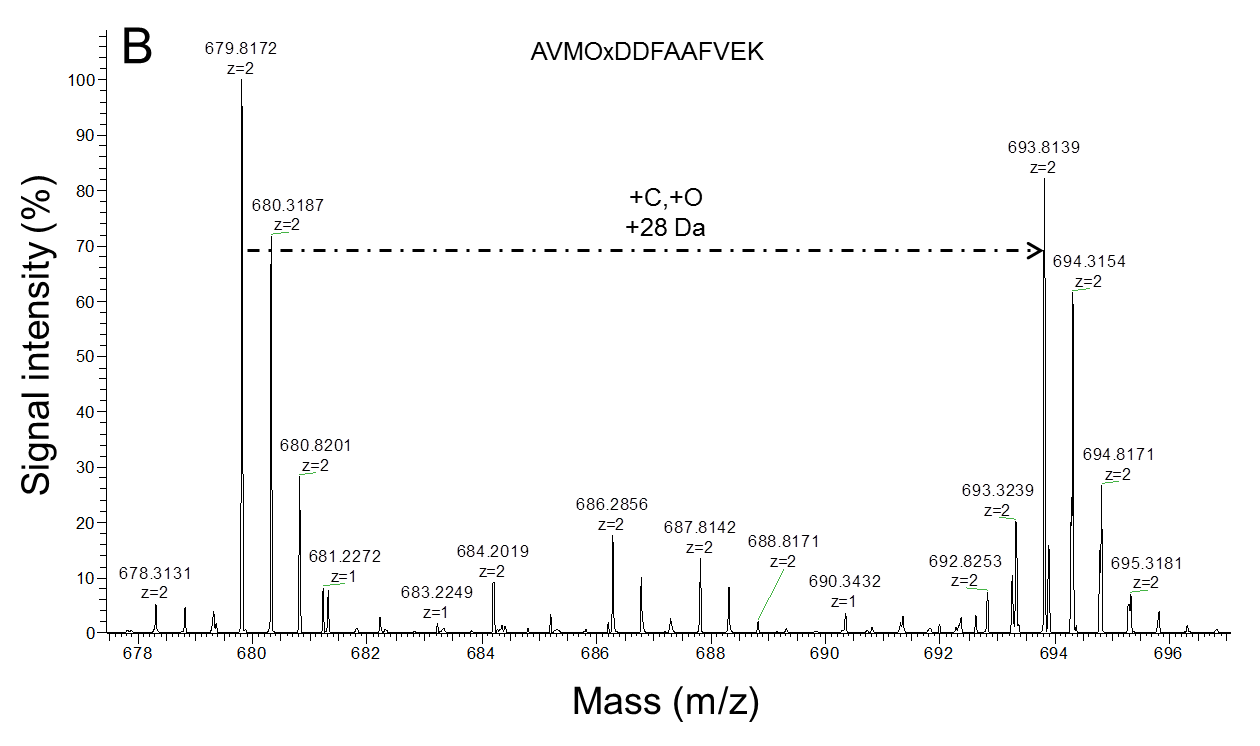


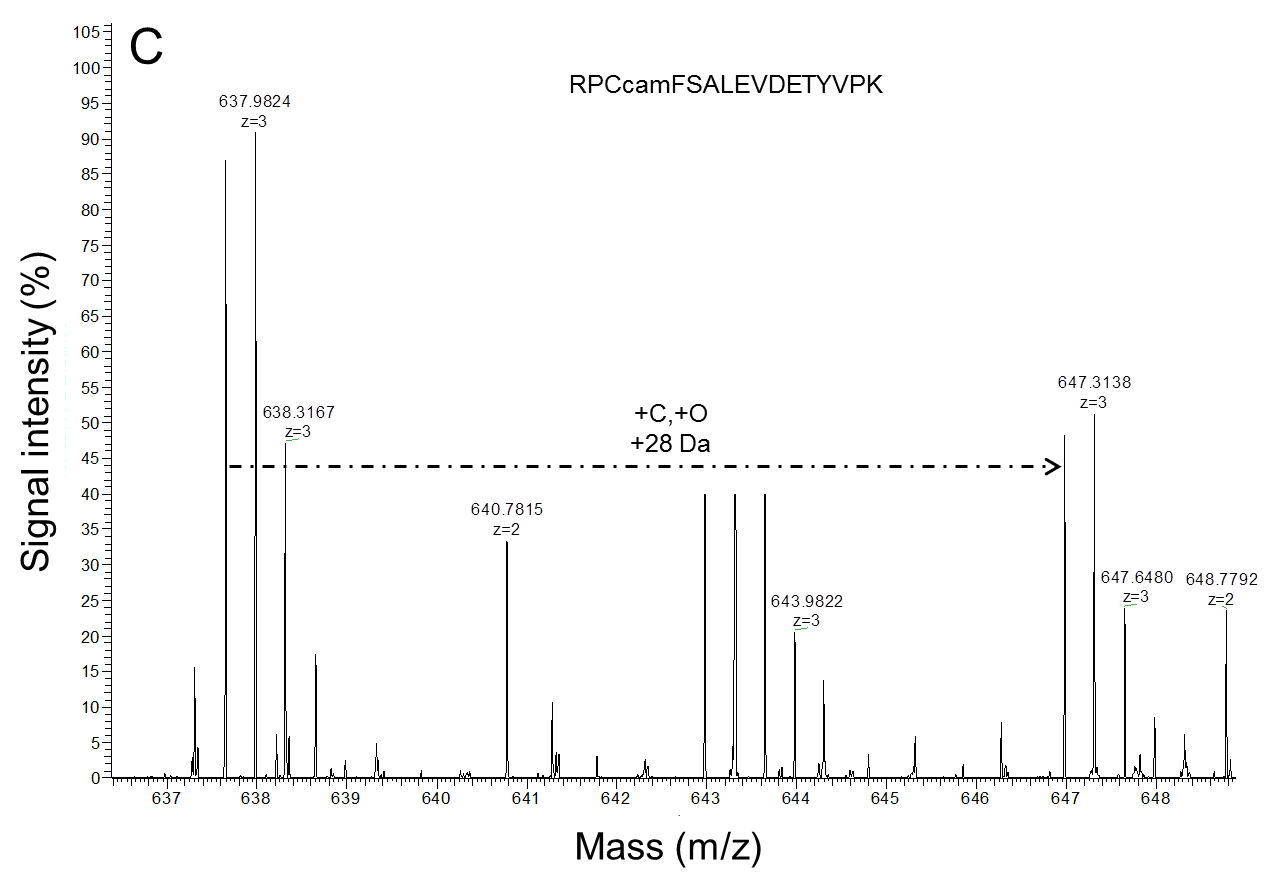


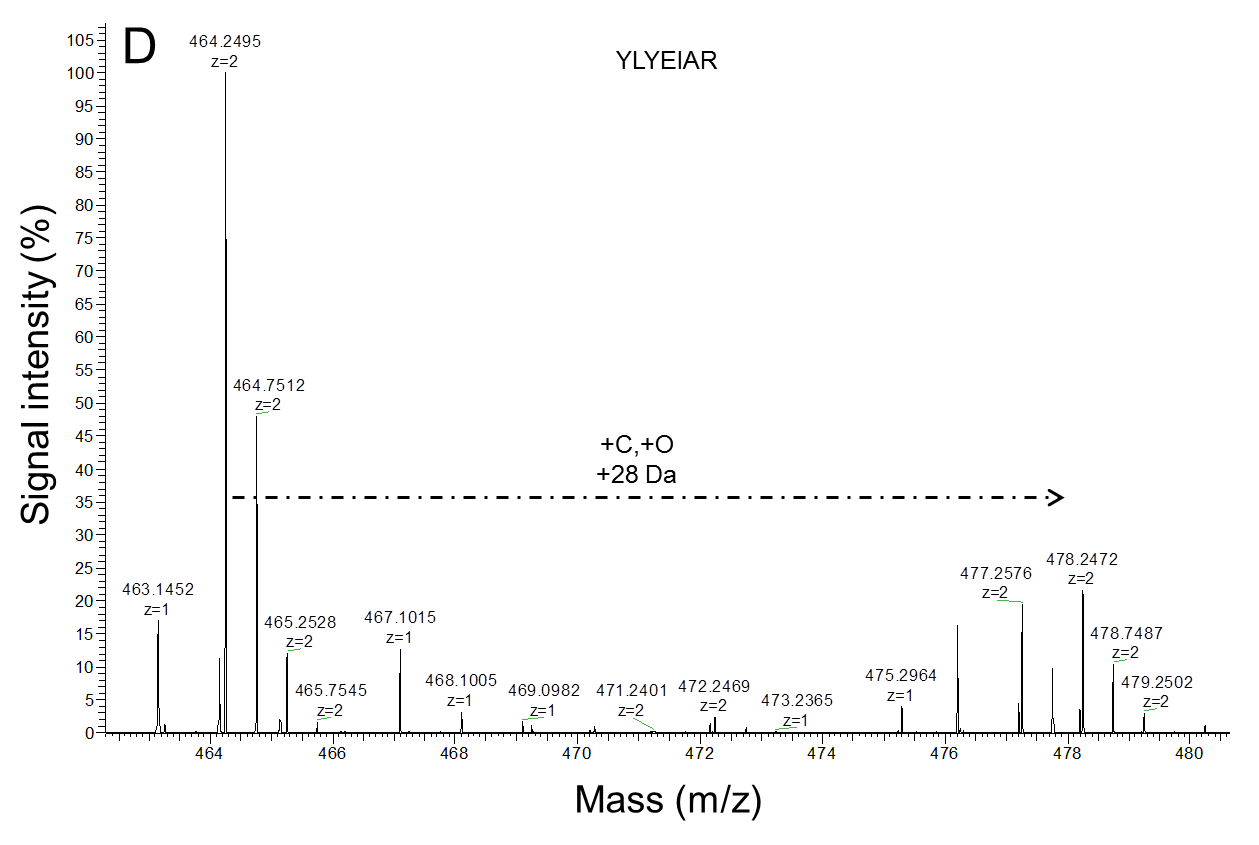


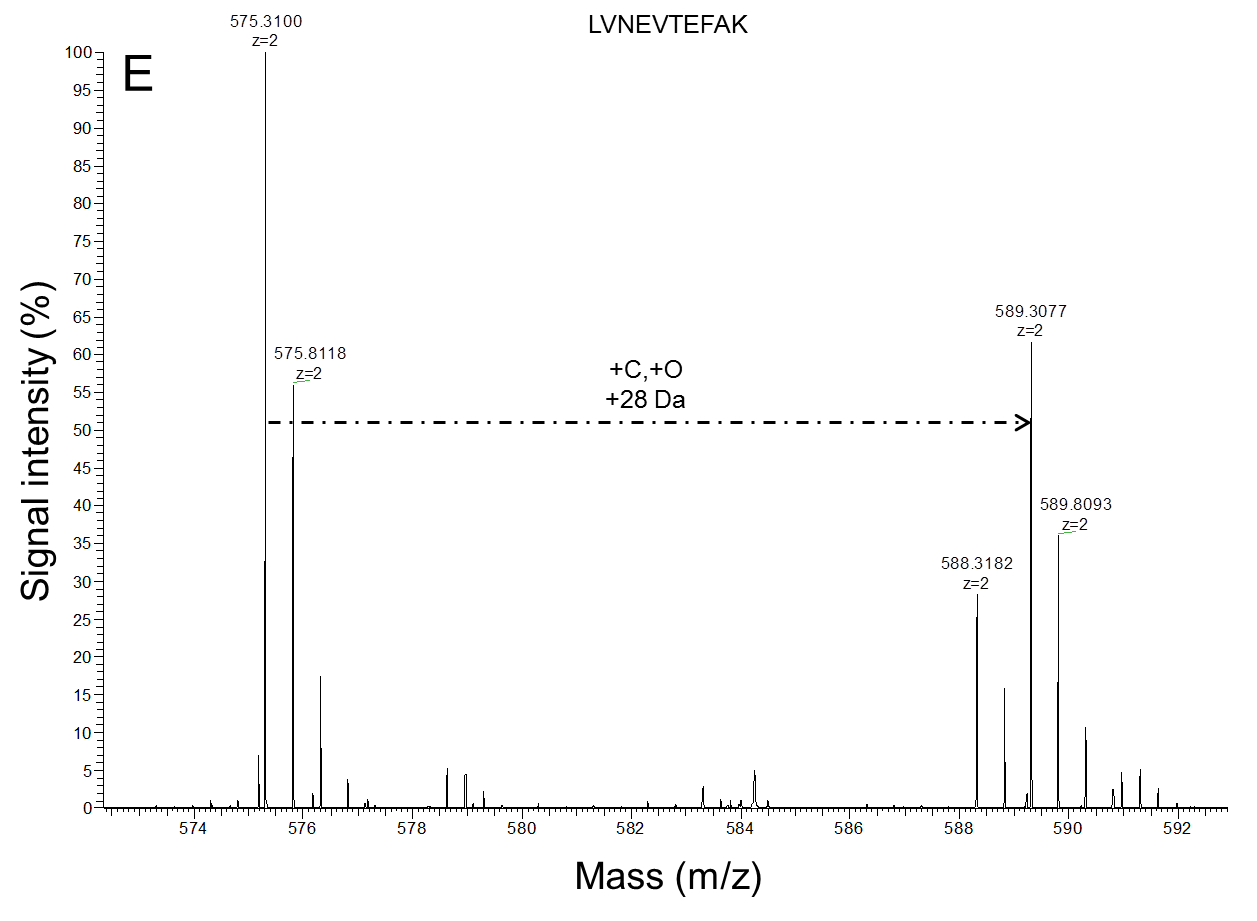


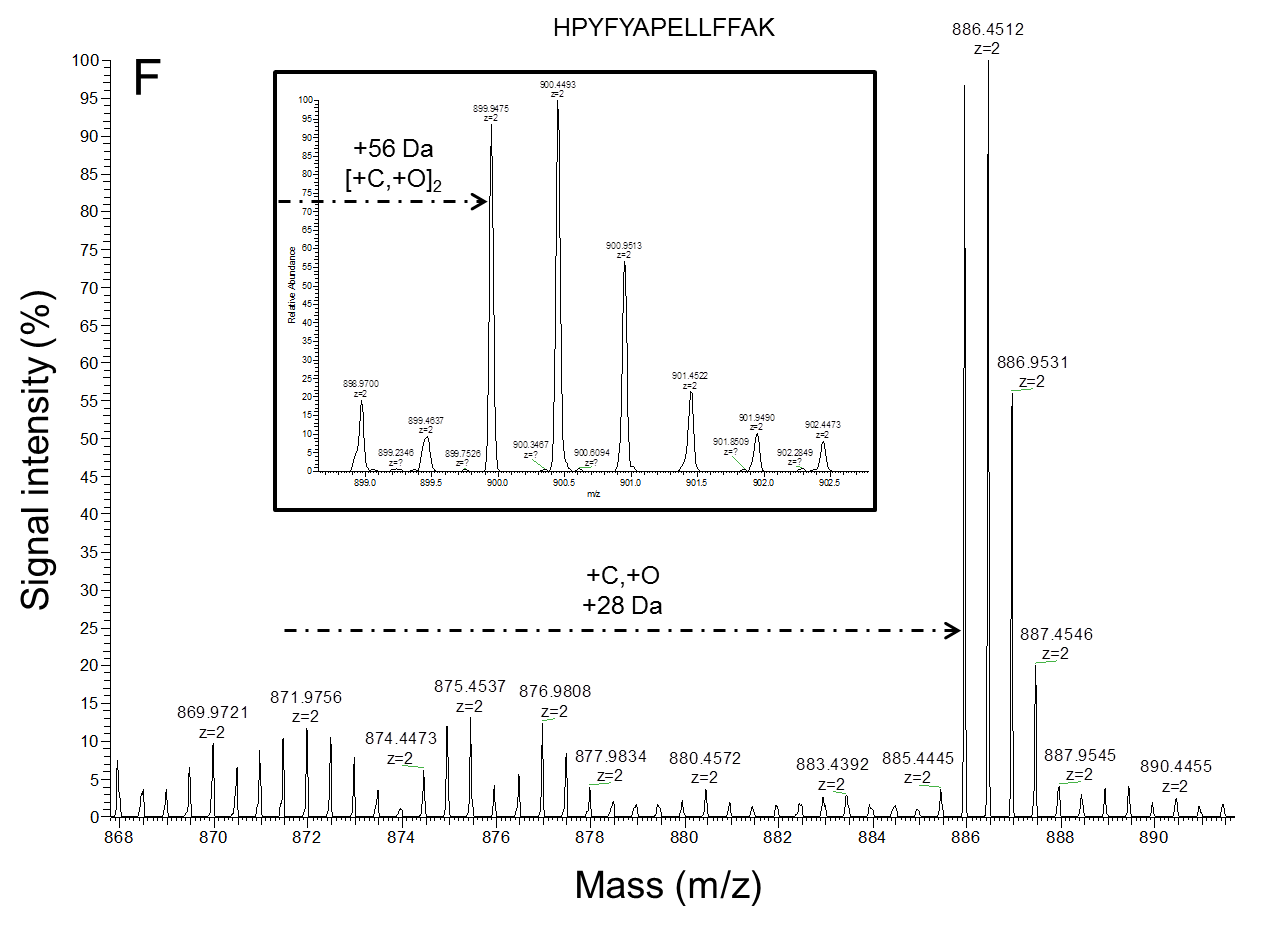


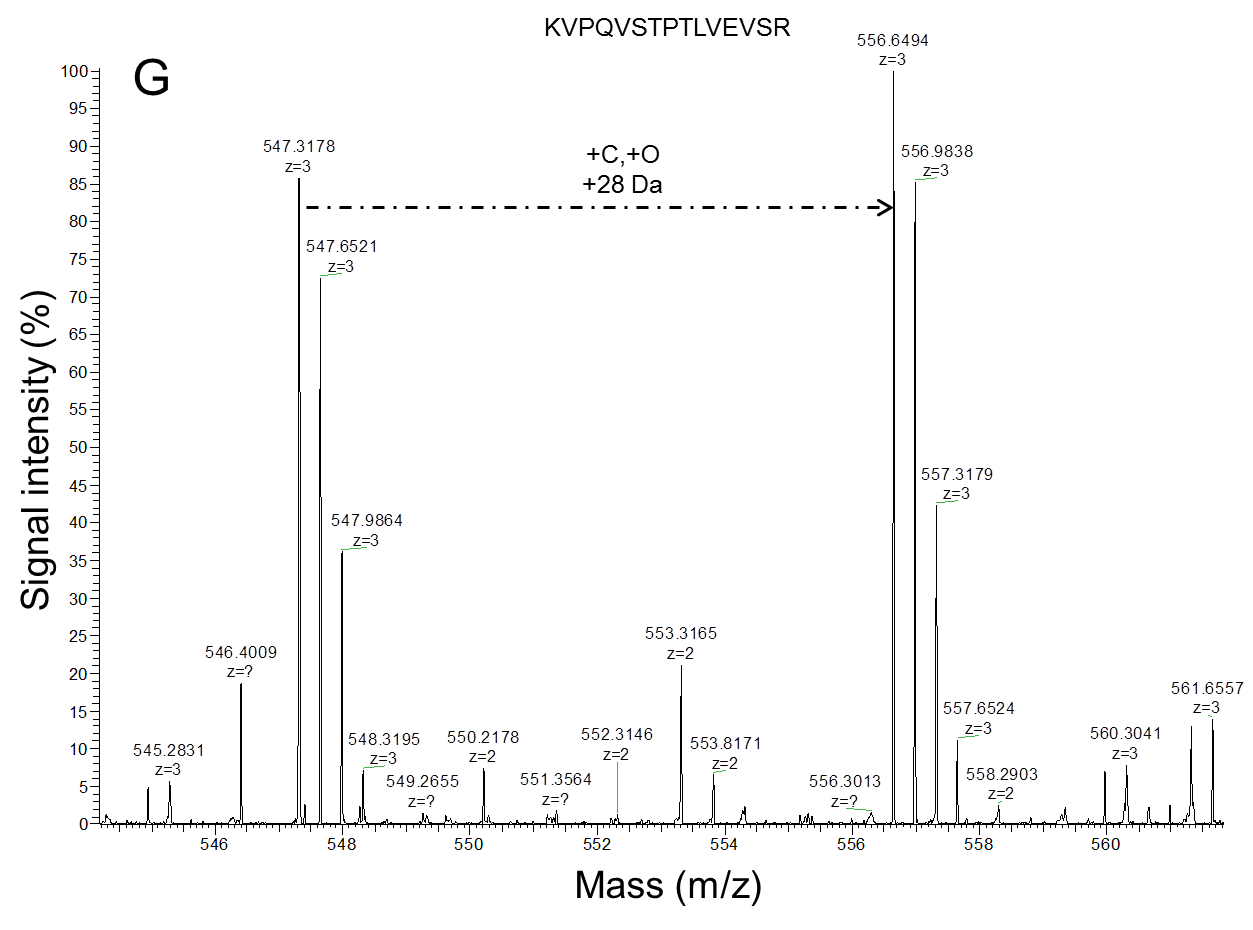


Fig. S32

Mass spectra of the laser-modified peptides of HSA seven-peptide standard. The modification was done with 300,000 laser pulses at wavelength 386 nm (pulse energy 269.54 μJ, fluence 7.78 mJ/cm^2^, intensity 51.9 GW/cm^2^. (A) AAFTECcamCcamQAADK, double carbamidomethylation on Cys, single formylation, (B) AVMoxDDFAAFVEK, single formylation, (C) RPCcamFSALEVDETYVPK carbamidomethylation on Cys, single formylation, (D) YLYEIAR, single (weak) formylation, (E) LVNEVTEFAK, single formylation, (F) HPYFYAPELLFFAK, prominent single formylation and weak double formylation (inset), (G) KVPQVSTPTLVEVSR, single formylation. Modifications, including mass changes, are indicated by dashed arrows.

7.8. Laser-induced formylation of insulin

Lastly, laser-induced modification of a large peptide, human insulin (~6 kDa), was examined. Insulin was poorly soluble in the standard ACN:water solvent (98:2 % v/v) but a solution of suitable concentration was obtained. Insulin was irradiated with laser pulses at 386 nm with conditions proven to produce formylation. Orbitrap FTMS spectra showed robust signal for the 5+ charge-state envelope for insulin (Fig. S33). No obvious modification was observed in control sample, although there is a small Na-adduct peak around 1167 m/z (Fig. S33A). Formylation (+28 Da) was observed following laser irradiation (Fig. S33B) with an apparent conversion rate of about 25-30%. No evidence for modifications of higher mass +44, +56, or +72 Da was found, but this is partially due to very poor signal/noise in these regions of the mass spectrum.

Laser-induced modification of proteins should be considered as a separate and more complicated challenge since protein structure significantly changes conditions for laser modification. However, results with insulin suggest that intact proteins can also be modified by the proposed approach.


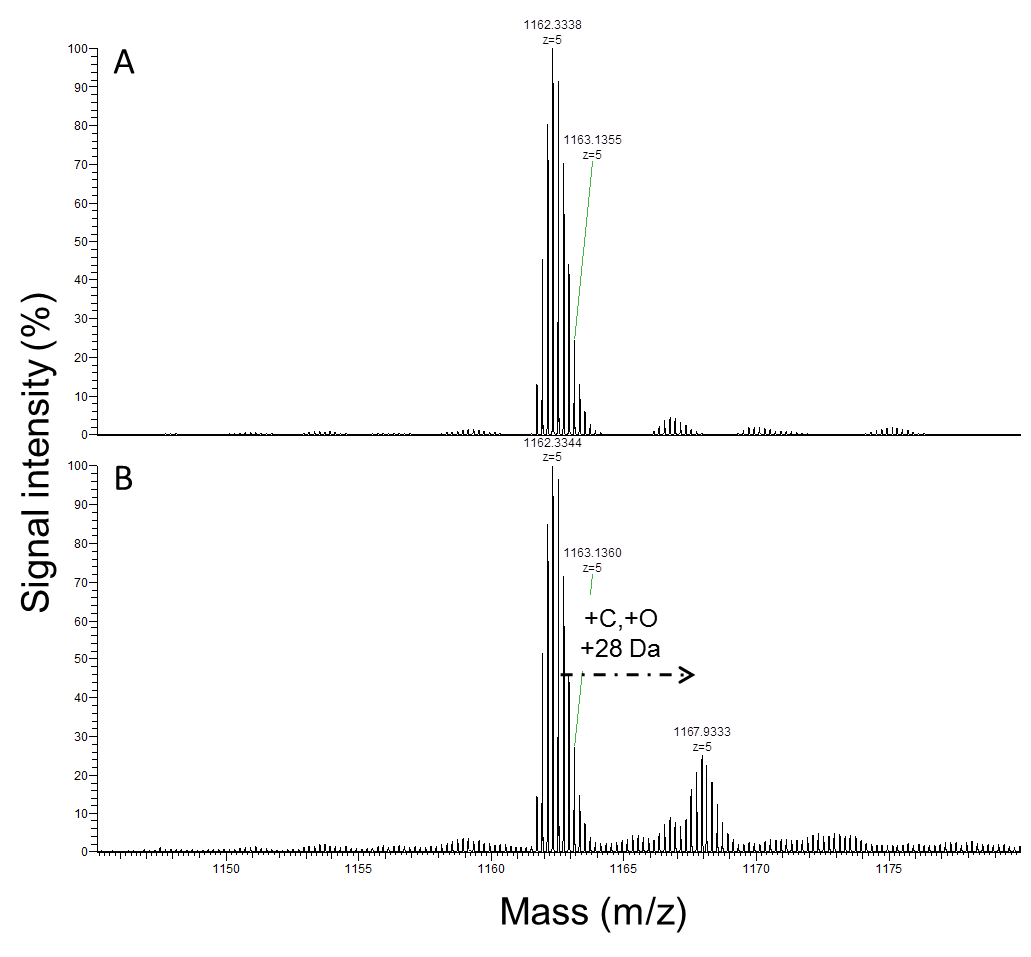


Fig. S33

Mass-spectrum of insulin modified in ACN:water (98:2 % v/v). The treatment was done by 480,000 laser pulses (pulse energy 17.66 μJ, fluence 878.4 μJ/cm^2^, intensity 5.86 GW/cm^2^). The multi-charge state (5+) isotope envelope of insulin was observed in FTMS spectra. (A) Control, non-irradiated, insulin, (B) laser-irradiated insulin. Formylation, with approximately 25-30% conversion rate, was observed (indicated by dashed arrow).


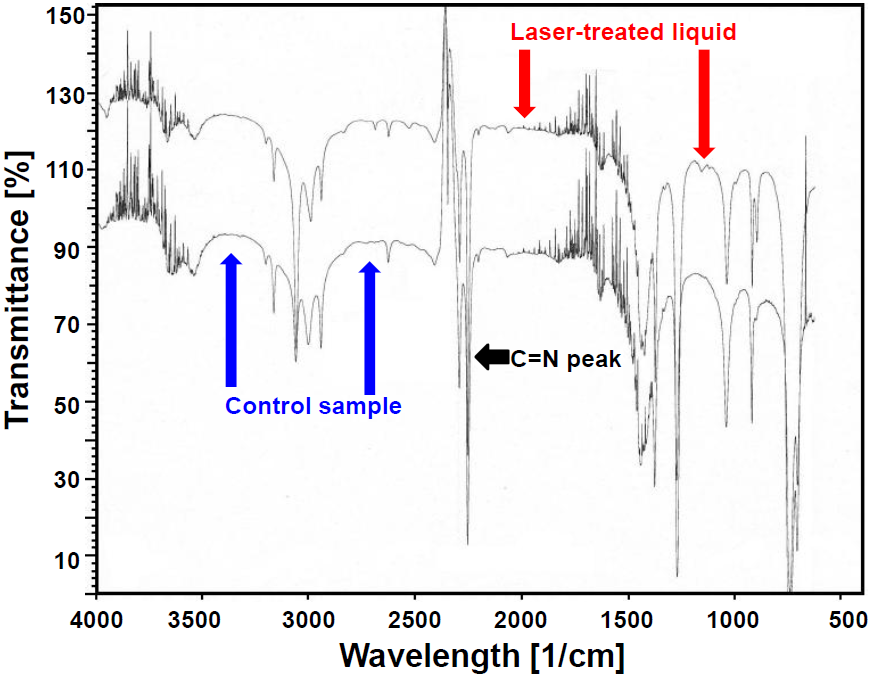


Fig. S 34. FTIR spectra of laser-treated mixture of acetonitrile and water (98:2 %% vol.). The laser treatment was done at wavelength 386 nm by 480000 pulses at fixed pulse energy 285 μJ per pulse; laser-beam diameter 6.5 mm.


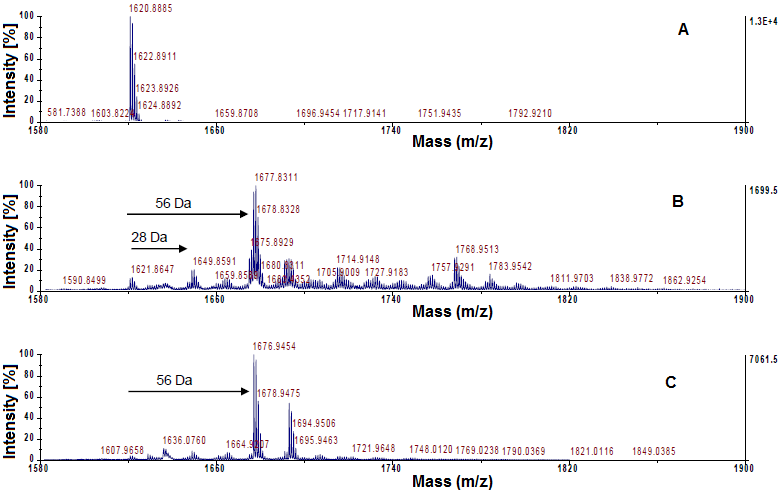


Fig. S 35. Mass-spectra of the test peptide modified at laser wavelength 772 nm (panel B; intensity 1.126·10^12^ W/cm^2^) compared to mass spectrum of the peptide modification at wavelength 386 nm (panel C; intensity 2.49·10^12^ W/cm^2^). Control mass spectrum of the untreated peptide is presented in panel A.
